# Exploring mechanisms of scar-free skin wound healing in adult zebrafish in comparison to mouse

**DOI:** 10.1371/journal.pgen.1012200

**Published:** 2026-06-24

**Authors:** İsmail Küçükaylak, Kai Halwas, Francisco Javier Martínez Morcillo, Nils Reiche, Manuel Metzger, Petra Comelli, Birgit Voigt, Jürgen Brinckmann, Sabine Eming, Matthias Hammerschmidt

**Affiliations:** 1 Institute of Zoology - Developmental Biology, University of Cologne, Cologne, Germany; 2 Department of Dermatology, University of Cologne, Cologne, Germany; 3 Department Biopolymers and Biological Interactions, FILK Freiberg Institute gGmbH, Freiberg, Germany; 4 Department of Dermatology and Institute of Virology and Cell Biology, University of Lübeck, Lübeck, Germany; 5 Center for Molecular Medicine Cologne (CMMC), Cologne, Germany; 6 Cologne Excellence Cluster on Cellular Stress Responses in Aging-Associated Diseases (CECAD), University of Cologne, Cologne, Germany; University of Pennsylvania School of Medicine, UNITED STATES OF AMERICA

## Abstract

Adult zebrafish have the ability to perfectly regenerate their skin after injury without leaving a scar behind. Yet, they intermediately form a collagen-rich granulation tissue that later fully regresses. In contrast, adult mammals lose this ability, resulting in persistent tissue fibrosis and scarring. We performed single-cell RNA sequencing and first HCR-based spatial transcriptomics to characterize the dynamics and heterogeneity of involved cell types during different stages of zebrafish cutaneous wound healing, focusing on macrophages and fibroblasts. Macrophage subclusters display pro-inflammatory and/or anti-inflammatory/pro-repair characteristics, and fibroblast subclusters characteristics of extracellular matrix formation and degradation, which largely co-exist during all stages of wound healing. Some wound-specific cells have a signature similar to that of myofibroblasts implicated in fibrotic healing in mammals. However, in contrast to mammalian myofibroblasts, they lack collagen expression, suggesting that they might only share the beneficial, but not the detrimental roles of their mammalian counterparts. Strikingly, zebrafish fibroblasts, in addition to expressing anti-fibrotic genes, express multiple genes with described pro-fibrotic effects in mammalian models. One of them is *plod2*, which encodes lysylhydroxylase 2. In cutaneous mouse wounds, *Plod2* is induced in fibroblasts by the macrophage-released Resistin-like molecule RELMα encoded by the *Retlna* gene, promoting the formation of DHLNL collagen crosslinks and thereby less resolvable fibrotic tissue. *retln* genes are absent from the zebrafish genome; nevertheless, *plod2* expression is initiated in zebrafish dermal fibroblasts upon wounding, in this case via TGFβ signaling, accompanied by increased collagen DHLNL crosslinking. Yet, both transgenic overexpression and genetic knock-out of *plod2* do not interfere with granulation tissue formation and regression, pointing to additional pathways assuring the resolution of temporary fibrosis in zebrafish skin wounds even in the presence of strong collagen crosslinking.

## Author summary

Wounds of humans and other mammals usually leave a scar behind, which can be both a cosmetic and a medical burden. These scars consist of fibrotic collagen-rich connective tissue that is formed by fibroblasts instructed accordingly by immune cells. In contrast, wounds of lower vertebrates like zebrafish can heal perfectly, without any scars. Even though their wounds temporarily form similar fibrotic tissue, it later disappears completely.

To understand what allows zebrafish to heal this way, we analyzed individual cells from their wounds at different healing stages. We were surprised to find that zebrafish still activate many of the same “fibrosis-promoting” genes seen in mammals. We looked closely at one of these, an enzyme called Lysylhydroxylase 2 (LH2) that is made by fibroblasts and strengthens collagen fibers. In mammals, LH2 expression is triggered by particular signals from immune cells, while in fish, the genes encoding these signals are missing. Yet, zebrafish use a different signal (TGFβ) to induce LH2 production and the same type of collagen fiber strengthening. Nevertheless, their tissue still returns to normal, even when excessive LH2 is supplied. Our findings suggest that zebrafish have natural ways to reverse fibrosis and restore healthy skin, offering clues that could help to reduce or even prevent scarring in humans.

## Introduction

Regeneration is defined by the almost perfect restoration of the lost or injured structure while repair is defined by an inflammatory response, connective tissue and finally scar formation, which is also observed in adult mammalian skin wound healing [1]. The healing of wounded skin upon injury is critical and skin integrity should be restored again swiftly since it constitutes the barrier to the outside world. Interestingly, adult zebrafish are able to fully regenerate their skin upon external insults, whereas mammalian scar-free regeneration is lost during embryogenesis [2–4]. Therefore, it is important to understand the mechanisms underlying this process in order to treat wound healing-related diseases properly.

The injury response initiates the formation of a new stroma underneath the neo-epidermis called the granulation tissue, where fibroblasts, innate immune cells and blood vessels invade the wounded area [5]. Macrophages, one of the main players during cutaneous wound healing, have essential roles by determining regeneration or scar tissue formation. Shortly after injury, tissue-resident macrophages are complemented by inflammatory monocytes that enter the damaged tissue to give rise to macrophages which clear cell debris and damaged extracellular matrix (ECM), while attracting other immune cells through the secretion of pro-inflammatory cytokines such as tumor necrosis factor α (TNF-α) and interleukin-1 (IL-1β). Later, they also play a critical role in resolving inflammation and to promote ECM tissue reconstruction, cell proliferation, and angiogenesis. These later macrophages are characterized by the secretion of anti-inflammatory factors such as IL-10 and factors like transforming growth factor β (TGFβ) that promote tissue repair [6–8]. These early and late functions are most likely carried out by distinct macrophage subpopulations [9,10], with an underlying macrophage plasticity and heterogeneity that turned out as much more complex than the initial concept of early pro-inflammatory, classically activated M1 macrophages and late alternatively activated M2 macrophages reducing inflammation and promoting tissue repair [11–13].

Similar to macrophages, fibroblasts of the granulation tissue of mammalian wounds have been reported to stem from different sources [14–17]. Fibroblasts residing in the adjacent dermis proliferate and migrate into the wound bed. Furthermore, monocytes entering the wound from the blood can differentiate into fibroblast-like cells called fibrocytes [18] – as also present in lesions associated with multiple fibrosing diseases [19,20]. Within the granulation tissue, dermal fibroblasts are stimulated by growth factors like TGFβ-1 and mechanical stress to synthesize and secrete collagens, which are the main constituents of the wound ECM, as well as other ECM molecules such as fibronectin, proteoglycans, glycosaminoglycans and hyaluronic acid [21]. Some fibroblasts are further stimulated to differentiate into myofibroblasts that in addition to ECM production form intracellular stress fibers and muscle proteins in order to establish tissue contraction [22]. The differentiation of fibroblasts to myofibroblasts during mammalian wound healing has also been described as a key feature of fibrosis; while they are beneficial for physiological tissue remodeling, they have adverse effects when becoming excessive, such as during hypertrophic scarring of wounded tissue [22,23]. However, not all, but particular fibroblast lineages/ subclusters of the granulation tissue are supposed to develop such adverse features [17,24–28], dependent on corresponding inflammatory priming which dictates on regenerative versus fibrotic repair [15,29].

The general consequence of fibrosis is an aberrant accumulation of ECM components and the loss of a normal tissue architecture [30–32]. In mammalian wounds, fibrosis denotes persistent excessive scar tissue, whereas in zebrafish wounds, cutaneous fibrosis is only transient and the original tissue structure is fully restored. An important factor contributing to collagen stability/persistence within the granulation tissue is the mode of collagen crosslinking via lysine residues that are already accordingly modified within the ER of fibroblasts. Lysylhydroxylase 2 (LH2) encoded by the *PLOD2* gene specifically hydroxylates lysine residues in the telopeptides of collagens. Two of such hydroxylysine residues – after subsequent lysyloxidase (LOX)-mediated oxidation of the amino group of one of them to an aldehyde group – can form divalent dihydroxy lysinonorleucine (DHLNL) crosslinks that can spontaneously and irreversibly condensate further to trivalent ring-shaped (desoxy)-pyridinoline crosslinks [33–36]. DHLNL crosslinks and their pyridinoline derivatives are supposed to make collagen fibers less susceptible to degradation and are a typical feature of mechanically stiff tissues such as bone and cartilage, whereas soft connective tissues such as normal skin are characterized by LH2-independent, lysine aldehyde-derived collagen crosslinks (hydroxylysinonorleucine, HLNL) [37,38]. Mouse skin wounds, on the other side, have been reported to display increased LH2 levels as well as increased DHLNL collagen cross-linking, while an alleviation of wound LH2 levels by genetic ablation of RELMα, a fibroblast-stimulating factor made by wound macrophages, reduced DHLNL levels as well as scarring [39]. Together, this points to an essential pro-fibrotic effect of LH2 during the scarring of mammalian skin wounds.

Here, we have investigated the cellular and molecular dynamics involved in scar-free cutaneous wound healing in adult zebrafish. We have formerly shown that full-thickness wounds in the skin of adult zebrafish introduced by a dermatology laser removing the epidermis, dermis, scales and subcutaneous adipocytes, fully close again within 10 hours after wounding. This closure is purely driven by morphogenetic movements within the epidermis, with keratinocytes starting to re-epithelialize the injured area even before innate immune cells have entered the wound bed [4,40]. However, inflammation by neutrophils and macrophages is required for the later recruitment of fibroblasts and the formation of a collagen-rich granulation tissue underneath the neo-epidermis [4]. We further showed that wound inflammation is maximal at two days post wounding (dpw), when granulation tissue starts to form, that granulation tissue is maximal at 4 dpw, and that granulation tissue is almost completely resolved at 8 dpw, coinciding with the regeneration of the subcutaneous adipose tissue and followed by the regeneration of skin appendages like scales [4], in sum constituting perfect and scar-free skin healing with only temporary fibrosis.

Utilizing scRNA sequencing, we have now compared the cellular compositions of unwounded skin and skin wounds of different stages of healing, as well as the transcriptional signatures of the different cell types, with particular focus on the roles and potential interactions of macrophages and fibroblasts. Our findings highlight the heterogeneity within the different cell types, with zebrafish dermal fibroblast subclusters actually expressing multiple genes associated with fibrosis in mammalian systems, including *plod2*, but also genes with no annotated function yet. Interestingly, neither gain nor loss of function of *plod2* altered the regenerative ability of zebrafish, suggesting that alternative factors are involved that make the difference to scarring in mammals. Collectively, our data set the base for systematic comparative analysis between perfectly regenerating and imperfectly healing model organisms as a promising approach to identify new factors that determine the quality of the reparative outcome.

## Results

### scRNA-seq-based identification and quantification of different cell types during different stages of cutaneous wound healing in adult zebrafish

**Fig 1:**
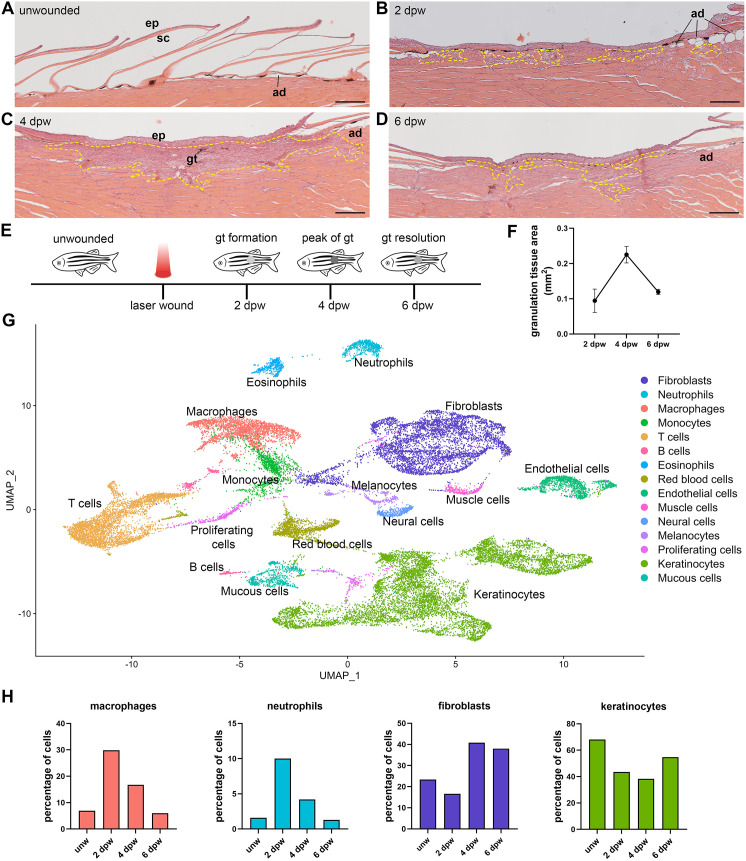
Single cell transcriptome analysis reveals the cellular composition of zebrafish cutaneous wounds. **(A-D)** Hematoxylin and eosin (H&E) staining of longitudinal skin sections showing the formation of granulation tissue (gt) beneath the wound, which reaches the maximum size in volume at 4 days post wounding (dpw) and then regresses (yellow dashed lines mark the granulation tissue). **(E)** Cartoon for the experimental design. Unwounded skin tissue and biopsies of wounds from 2, 4 and 6 dpw were collected for single cell RNA sequencing. **(F)** Quantification of granulation tissues at 2, 4 and 6 hpf, as shown in panels **(A-D)**. See also Fig 2j of Ref. [4]. **(G)** UMAP representation of single cell RNA sequencing data after integration of four datasets and cell clustering results. **(H)** Percentages of macrophages, neutrophils, fibroblasts and keratinocytes relative to the total numbers of the four cell types across the time points according to single cell RNA sequencing data set. Numerical values for panels F and H can be found in S2 Data. Scale bars: A-D = 200 µm. ep: ad: adipocytes; ep: epidermis; gt: granulation tissue; sc: scale.

To better understand the cellular dynamics underlying the reversible fibrosis that occurs during scar-free wound healing in adult zebrafish, we performed comparative single-cell RNA-sequencing (scRNA-seq) of skin biopsies from unwounded skin and from skin wounds at 2 dpw, when granulation tissue has started to form, at 4 dpw, when granulation tissue size peaks and at 6 dpw, when granulation tissue regresses (Fig 1A-1F). scRNA-seq analysis and Uniform Manifold Approximation and Projection (UMAP) representation of all time points integrated revealed the different major cell types present during zebrafish cutaneous wound healing (Figs 1G and S1). The top 10 differentially enriched genes in the specific clusters were used to identify the cell types at the four investigated time points (S2 Fig). The majority of the wound cells were comprised of keratinocytes, fibroblasts and immune cells. In line with former histological data [4], scRNA-seq revealed a significant increase in numbers of wound macrophages (5.6 fold) and neutrophils (8.4 fold) at 2 dpw compared to unwounded skin, with numbers progressively dropping again at 4 dpw and 6 dpw, although remaining higher than in unwounded skin (Fig 1H). In contrast, fibroblasts only started to display increased numbers compared to unwounded skin at 4 dpw, and numbers remained comparably high even at 6 dpw (Fig 1H), when the size of the granulation tissue has dropped back to its size at 2 dpw (Fig 1F). We can only speculate about the reasons for the latter. However, according to our former *col1a2* in situ hybridizations [4] and TUNEL-stainings [41], fibroblasts are primarily lost from the regressing granulation via emigration, rather than cell death - and such wound-adjacent emigrating fibroblasts might still have been contained in our scRNA-seq samples.

### Cellular heterogeneity of wound macrophages

**Fig 2:**
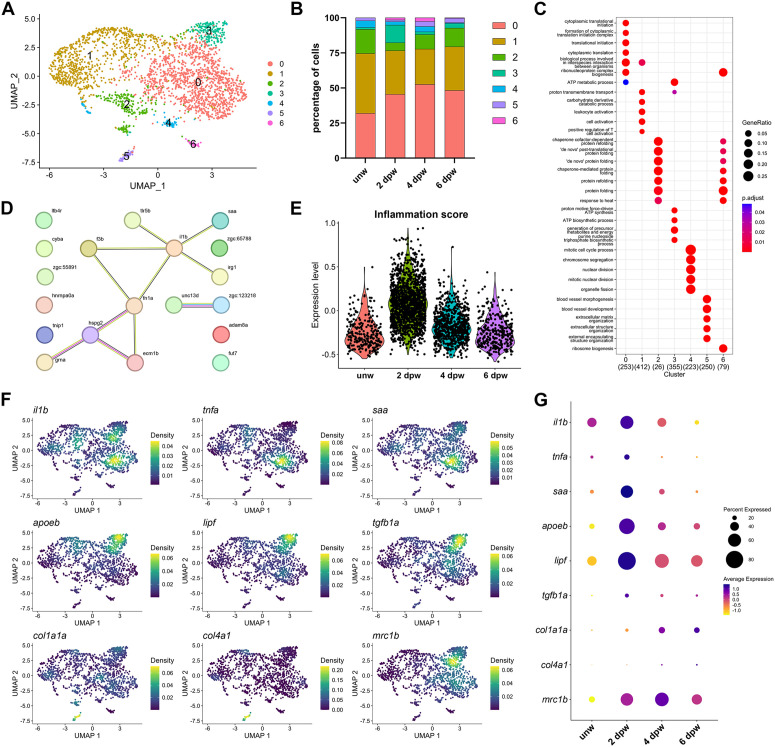
Characterization of distinct macrophage subpopulations. **(A)** UMAP representation of macrophage subclusters of four datasets. **(B)** Percentage of macrophage subcluster numbers across the time points. Numerical values for panel B can be found in S2 Data. **(C)** Gene ontology (GO) analysis of biological processes of macrophage subclusters at 2 days post wounding (dpw). The GeneRatios, indicated by the diameter of dots, represent the proportion of input genes associated with a given GO term, calculated as the number of genes annotated to the term divided by the total number of input genes used in the analysis; colors represent the significance of the enrichment (p.adjust). **(D)** STRING analysis [128] of genes up-regulated in macrophages at 2 dpw in comparison with unwounded samples, yielding the shown pathway “GO:0006954-Inflammatory response”. **(E)** Violin plot of inflammation scores of macrophages in unwounded skin and at 2 dpw, 4 dpw and 6 dpw, calculated via module scoring using Seurat’s AddModuleScore function with the genes shown in panel **D. (F)** UMAP and density plot of the expression of some selected genes at 2 dpw for pro- and anti-inflammatory phenotypes of macrophages. **(G)** Dot plot showing the expression level of selected genes from different macrophage subclusters across the time points. Circle size represents the percentage of all macrophages expressing the genes and the color represents the average abundance of gene transcripts within the positive macrophages at the indicated stage relative to the other three stages (blue/ + 1 = stage with highest average expression; yellow/-1 = stage with lowest average expression).

Data obtained for cutaneous wounds in mouse [9,10,24,42–44] as well as wounds of other tissues in zebrafish [45–49] point to a large degree of cellular heterogeneity among both macrophages and fibroblasts. We therefore next analyzed macrophage and fibroblast clusters of our zebrafish skin wounds in more detail. For macrophages, we identified seven different subclusters (Figs 2A and S3). Subclusters 0–5 were present at all four investigated stages, including unwounded skin. In contrast, subcluster 6 was wound-specific, but only found with comparably low cell numbers at 2 dpw and 4 dpw, suggesting that it might play a minor executive role during granulation tissue dynamics (Fig 2B).

**Fig 3:**
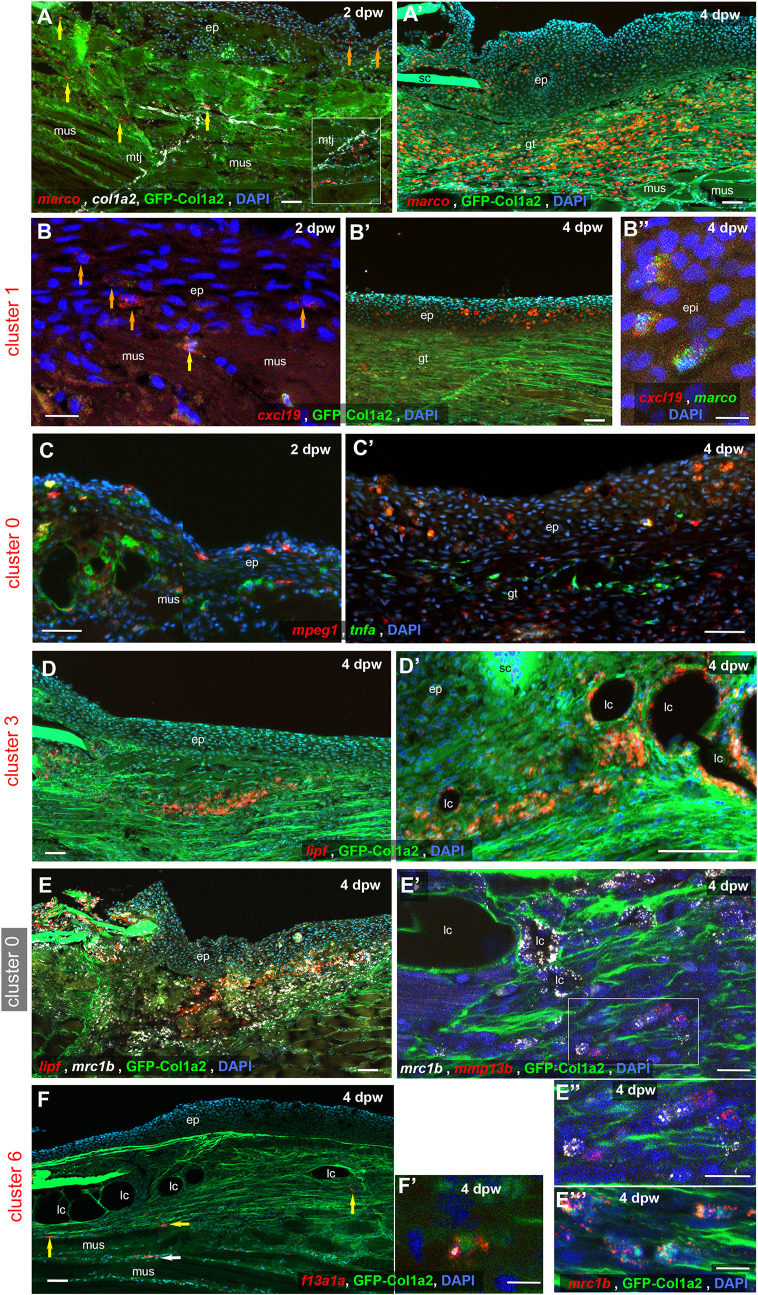
Spatial expression patterns of selected macrophage genes, relative to GFP-labelled Col1a2 protein. Sections through wounds of indicated stages (2 dpw, 4 dpw), stained via HCR with indicated probes (A,B,D,E,F) or labelling of cells with *Tg(mpeg1:mCherry)* and *Tg(tnfa:GFP)* transgenes **(C)**, counterstained with DAPI for nuclear DNA. (A,B,D,E,F) were further co-labeled for type I collagen protein N-terminally fused with GFP, encoded by the recombined *ki(col1a2:GFP-col1a2)* locus (see Materials and Methods; fish were heterozygous for the knock-in). Overviews shown in (A,A’,B’,D,F) show approximately one half of the wound, with the wound edge on the left side, and the center of the wound on the right side. In (A) and **(B)**, orange arrowheads point to positive cells in the neo-epidermis, orange arrows to positive cells in underlying regions where the granulation tissue is formed. In **(F)**, yellow arrows point to positive cells in granulation tissue regions with lower collagen densities where collagen-free lacunae are formed, the white arrow to a positive cell in the endomysium. In **(B)**, inset framed in white shows proximal continuation of shown myotendinous junction (mtj). (E”) shows magnified view of the part of E’ boxed in white. For details see text. Abbreviations: bk: basal keratinocytes; ep: epidermis; gt: granulation tissue; lc: lacuna; mtj: myotendinous junction; mus: skeletal muscle fibers; sc: scale. Scale bars: 50 μm (A,A’,B,C,D,D’,E,F), 20 μm (C,E’), 10 μm (B,”E,”E”’,F’).

The different subclusters were further characterized via gene ontology (GO) enrichment analyses (Fig 2C), based on significantly upregulated genes (S3 Fig), by comparing the expression levels of genes (even if they were not among the top 10) with formerly reported pro-inflammatory, anti-inflammatory and/or tissue repair functions (S4 Fig). In addition, we performed first studies of their spatial distribution within the wound, applying either imaging with transgenic marker lines or single and double Hybridization Chain Reaction (HCR) analyses for transcripts of selected marker genes (Fig 3). Subcluster-specific markers were selected based on their subcluster specificity, their enrichment upon wounding, their GO annotation and their mentioning in recently published characterizations of different macrophage subtypes [49–51].

HCR analyses with the broad macrophage marker *marco* (Macrophage receptor with collagenous structure) [52] (Figs 3 and S17 for UMAP) revealed that at 2 dpf, macrophages were mainly present in the neo-epidermis (which with approximately 10 cell layers is much thicker than the regular unwounded epidermis (4–5 cell layers) [4]). Underneath the neo-epidermis *marco*-positive macrophages were comparably sparse and mainly localized at the lateral edges of the wound and, together with *col1a2*-positive fibroblasts, along myotendinous junctions of the underlying skeletal muscle (Fig 3A). At 4 dpf, in contrast, high numbers of *marco*-positive macrophages were found within the granulation tissue (Fig 3A), suggesting that they, together with fibroblasts, have been recruited to the wound from adjacent tissues (skin and muscle) between 2 dpw and 4 dpw.

**Fig 4:**
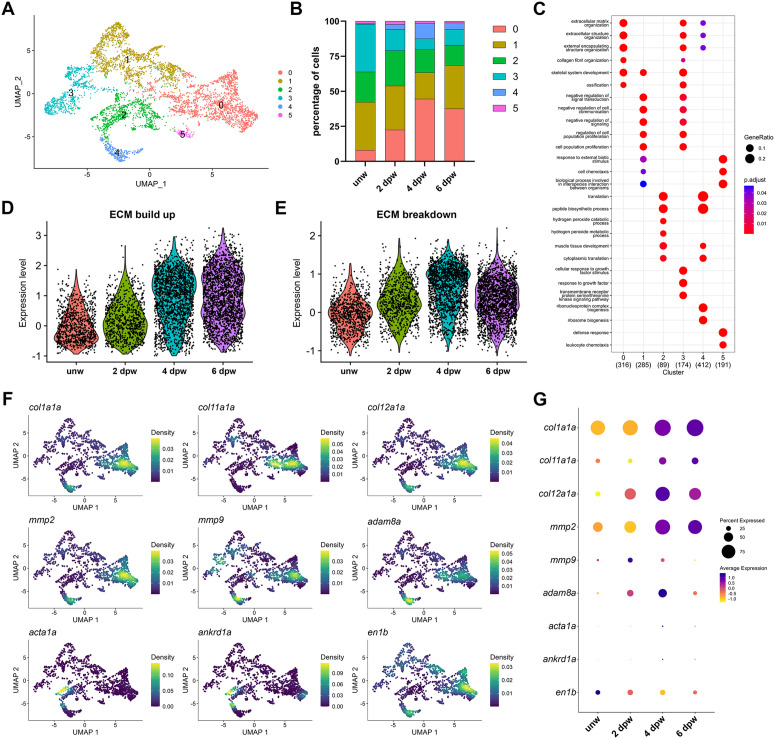
Characterization of distinct fibroblast subpopulations. **(A)** UMAP representation of fibroblast subclusters of four datasets. **(B)** Percentage of fibroblast subcluster numbers across the time points. Numerical values for panel B can be found in S2 Data. **(C)** Gene ontology (GO) analysis of fibroblast subclusters at 4 days post wounding (dpw). **(D, E)** Violin plots showing the expression level of ECM build up and ECM break down related genes in fibroblasts across the time points. **(F)** UMAP and density plot of the expression of some selected genes at 4 dpw, associated with ECM regulation or fibrosis. **(G)** Dot plot showing the expression level of the selected genes across the time points. Circle size represents the percentage of fibroblasts expressing the genes and the color represents the average abundance of gene transcripts within the positive fibroblasts at the indicated stage relative to the other three stages (blue/ + 1 = stage with highest average expression; yellow/-1 = stage with lowest average expression).

Inflammation of wounded skin is initially triggered by keratinocytes that upon reception of pathogens or damage-associated molecular patterns (DAMPs) release different cytokines or chemokines, including different CCL and CXCL chemokines that can attract immune cells [53,54]. Such recruited immune cells can then elicit pro-inflammatory effects themselves, attracting more immune cells and/or stimulating immune cell proliferation. According to our scRNA-seq analyses, macrophage cluster 1 was the only subcluster with a high score for GO terms “leukocyte activation” and “cell activation” (Fig 2C), and *cxcl19* was the most strongly enriched gene of this cluster, while it was hardly expressed in other macrophage subclusters or in keratinocytes (S3, S5 Figs). It encodes a chemoattractant which in other physiological contexts has been reported to signal via CXCR4 receptors [55], while CXCR4 has been shown to be involved in multiple pathological mechanisms of fibrosis [56]. Indeed, according to our scRNA-seq data, the two zebrafish homologs, *cxcr4a* and *cxcr4b* were strongly expressed in macrophages of all subclusters and in all other immune cell types (S5 Fig), while our HCR analyses showed that both at 2 dpw (Fig 3B) and 4 dpw (Fig 3B’), *cxcl19* was almost exclusively expressed by (also *marco*-positive; Fig 3B”) macrophages localized in deeper layers of the neo-epidermis. Together, this suggests that cluster 1 macrophages located in the neo-epidermis might send Cxcl19 signals to the adjacent un-damaged skin and the underlying tissues to attract other immune cells, thereby triggering further wound inflammation and granulation tissue formation.

Yet, genes with more established pro-inflammatory roles, such as *il1b*, *tnfa* and *saa*, encoding interleukin 1, tumor necrosis factor α, and serum amyloid A, respectively [57–61], were hardly expressed in cluster 1, but mainly in cluster 0 (Figs 2F and S5) - although in an only partially overlapping manner (S6 Fig), pointing to rather high heterogeneity even within annotated subclusters themselves (see also below). Imaging *tnfa*-positive cells with an established *Tg(tnfa:GFP)* transgene [62], we found them to display a different localization than *cxcl19*-expressing macrophages (see above) and co-labelled *mpeg*-expressing cells (most likely co-expressing *cxcl19*; S6 Fig). Thus, *tnfa*-positive cells largely remained below the epidermis in the forming (2 dpw) and formed granulation tissue (4 dpw) (Fig 3C), suggesting that they promote further wound inflammation primarily via signals emanating from the granulation tissue itself.

In comparison, *apoeb*, *lipf*, *tgfb1a* genes, encoding apolipoprotein E, lipase F, and transforming growth factor β1, respectively, for which rather anti-inflammatory and repair/fibrosis-stimulating roles have been reported [10,45,58,61], were mainly expressed in cluster 3 (Figs 2F and S5) - although again not in fully overlapping manner, with *tgfb1a* expressed only in a few cells, but *lipf* in almost all cells of the cluster (S6 Fig) Spatially, even more so than *tnfa*-positive cells of cluster 0, *lipf*-positive cells of cluster 3 remained strictly within the granulation tissue, where they, however, displayed a much more restricted spatial distribution therein. Thus, they were only found in upper and more marginal regions of the 4 dpw granulation tissue (Fig 3D), where type I collagen density is lower and where collagen-free lacunae have started to form that – based on their shape - most likely accommodate regenerating adipocytes (Fig 3D’; compare shape of lacunae with shape of subcutaneous adipocytes in Fig 1).

Of note, the expression of both *tnfa* in cluster 0 and *tgfb1a* in cluster 3 dropped between 2 dpw and 4 dpw (Figs 2G and S5). For TNFα, this is in line with its potential early proinflammatory role, while for TGFβ, its potential later anti-inflammatory/pro-repair task might be taken over by the concurrent up-regulation of *tgfb1a* expression in fibroblasts (see below). Expression of *il10*, a strong anti-inflammatory marker and regulator in mammalian wounds, was only detected in unwounded skin, but not in macrophages of any subcluster and at any stage of our zebrafish wounds (S5 Fig).

Pro-inflammatory cluster 0 (Fig 2B) and expression of selected pro-inflammatory cluster 0 marker genes, alongside with anti-inflammatory marker genes of other subclusters (Fig 2G), were also present at 4 dpw and 6 dpw, although the overall inflammation score was maximal at 2 dpw and dropped back to unwounded skin levels during later stages (Fig 2E; see legends of panels D and E for more details). Interestingly, cluster 0, in addition to its pro-inflammatory role might also play a (later?) anti-fibrotic, ECM-degrading role. This is particularly suggested by the strong and continuous expression of *mrc1b* (Figs 2G and S3 and S5), encoding a mannose receptor formerly shown to be required for collagen phagocytosis and degradation by M2-like macrophages in the mouse skin [63]. Indeed, in our HCR analyses, these *mrc1b*-positive cells were localized rather broadly within the 4 dpw granulation tissue (Fig 3E), forming tight cell associations at the circumference of developing early-stage collagen-free lacunae that will accommodate regenerating subcutaneous adipocytes (Fig 3E’). In addition, some of them contained intracellular non-fibrillar GFP-labelled Col1a2 (Fig 3E”’), while others were associated with *mmp13a*-expressing cells (Fig 3E”). Of note, Mmp13a most likely is THE major collagen type I collagenase of zebrafish - the zebrafish lacks MMP1 and MMP8, the two other primary collagen type I collagenases in human [64]. Strikingly, according to our scRNA-seq data, *mmp13a* was primarily expressed by neutrophils (S7 Fig). Together, this points to a tight cooperation of neutrophils and macrophages of subcluster 0 to degrade and remove collagens from the granulation tissue, thereby generating space for the regenerating subcutaneous adipose tissue (see also Discussion).

Cluster 5 was particularly characterized by its high score for GO term “extracellular structure organization” (Figs 2C and S4). Cluster 5 macrophages expressed for instance *col1a1a* (Figs 2F,2G and S5) encoding fibrillar collagen type I, which is usually and mainly produced by fibroblasts [4,15,65] (see below), as well as *col4a1, col4a2* and *lama4* (S3, S5 Figs), encoding integral components of the skin basement membrane - which also has to regenerate at the epidermal-dermal junction, a function mainly performed by keratinocytes and fibroblasts. Interestingly, laminins and collagen IV have also been implicated with fibroblast activation and fibrosis in different mammalian systems [66]. Furthermore, macrophages have been formerly reported to directly contribute to collagen and ECM production upon heart injury in zebrafish and mouse [67]. Together, this suggests that macrophage subcluster 5 might, in addition to stimulating fibroblasts, directly contribute to ECM production during granulation tissue formation in zebrafish skin wounds.

Cluster 6, the only wound-specific of the seven macrophage subclusters (Fig 2B), was characterized by high expression levels of genes like *f13a1a*, *esr2a* and *cpn1* (S3, S5 Figs). *f13a1a* encodes transglutaminase factor XIII-A, which is mainly involved in blood clotting – in contrast to mammals, fish skin wounds lack blood clots [4] – but which in mammals has also been described at the surface of alternatively activated M2 macrophages to terminate inflammation and to promote wound healing and phagocytosis [68]. Consistent with the comparably low cell numbers obtained for cluster 6 in our scRNA-seq analyses (Fig 2B), HCR only detected comparably few *f13a1a-*positive cells in the 4 dpw zebrafish granulation tissue. In contrast to *mrc1*-positive macrophages, they did not associate with each other, but usually occurred as single cells, preferentially close to the regions where the aforementioned collagen-free lacunae are formed, but also within the endomysium of wound-adjacent skeletal muscle (Fig 3F). Of note, similar to *mcr1b* cells, at least some of them, although most likely not expressing *mrc1b* (S6 Fig), contained intracellular GPF-Col1a2 (Fig 3F’), suggesting that they might also be involved in collagen removal. *esr2a* encodes estrogen receptor 2. In mice, Esr2 has been described to be required in keratinocytes [69] and Esr1 in macrophages [70] to allow proper cutaneous wound healing, while in zebrafish, it has been shown to accelerate heart regeneration [71]. Finally, *cpn1* encodes carboxypeptidase N, which to our knowledge had not been reported to be made by macrophages thus far, which, however, is known to inactivate bradykinin and anaphylatoxins such as C3a and C5a, thereby eliciting anti-inflammatory effects [72]. Furthermore, GO analysis demonstrated an enrichment of cluster 6 for de novo protein folding and distinctively for ribosome biogenesis (Fig 2C), pointing to strong protein-synthesizing activity. In sum, this points to cluster 6 as a wound healing-specific macrophage subpopulation with particular activation modes and a unique combination of anti-inflammatory and pro-regenerative effects.

Together, our macrophage subcluster analyses indicate that, in line with revised concepts in mammals [12], macrophages of zebrafish skin wounds do not follow the concept of stage-specific pro- and anti-inflammatory polarization, but that the different (and to some extent opposing) tasks fulfilled by macrophages are distributed among a whole spectrum of subpopulations that largely co-exist during the different stages of wound healing.

### Cellular heterogeneity of wound fibroblasts

Analysis of fibroblasts during the different stages of cutaneous wound healing revealed at total of six different subclusters (Fig 4A). Of all subclusters, cluster 0, which is for instance characterized by the cluster-specific co-expression of *ogna, cilp, col2a1b and fn1* (see below for details), showed the highest increase in cell numbers induced by wounding (from 8% of all fibroblasts in unwounded skin to 45% at 4 dpw; Fig 4B). In addition, one new wound-specific fibroblast subcluster not present in uninjured skin showed up, cluster 4, characterized for instance by the cluster-specific expression of *col18a1a* and *plaub* (see below for details) and comprising 11% of all fibroblasts at 4 dpw (Fig 4B).

**Fig 5:**
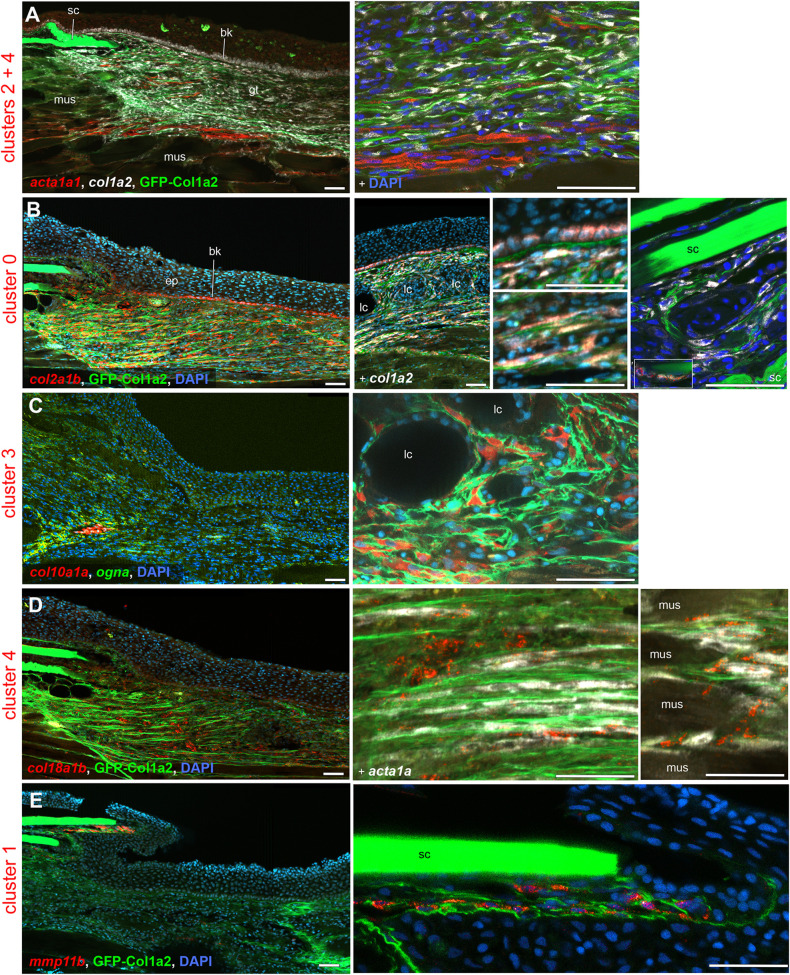
Spatial expression patterns of selected fibroblast genes, relative to GFP-labelled Col1a2 protein. Sections through wounds at 4 dpw, stained via HCR with indicated probes, counterstained with DAPI for nuclear DNA, and co-labeled for type I collagen protein N-terminally fused with GFP, encoded by the recombined *ki(col1a2:GFP-col1a2)* locus (see Materials and Methods; fish were heterozygous for the knock-in). Overviews shown in left-most images of each panel show approximately one half of the wound, with the wound edge on the left side, and the center of the wound on the right side. Inset in right-most image of (B) shows, as positive control, a *col2a1b*-positive (red) cell from the same section. For details, see text. Abbreviations: bk: basal keratinocytes; ep: epidermis; gt: granulation tissue; lc: lacuna; mus: skeletal muscle fibers; sc: scale. Scale bars in all panels: 50 μm.

Interestingly, parts of clusters 2 and 4 displayed a transcriptional signature pointing to myofibroblast-like cell functions, with specific expression of genes encoding muscle actin (*acta1a1*; Figs 4F,4G and S10), a marker of myofibroblasts in burned mammalian wounds [73], the muscle-specific intermediate filament desmin (*desma*; S10 Fig), the muscle/myofibroblast-specific cell-adhesion molecule M-cadherin [74] (*cdh15*; S10 Fig), or the Ankyrin repeat domain-containing protein 1 (*ankrd1a*; Figs 4F,4G and S10), also known as Cardiac ankyrin repeat protein (CARP), a stress-inducible myofibrillar protein and known target of TGFβ signaling, which, although mainly implicated in heart pathologies and carcinogenesis, was also reported to be elevated during hypertrophic skin scarring [75], while gene knockout led to reduced granulation tissue formation during cutaneous wound healing [76]. In mammalian skin wounds, myofibroblasts are considered as a crucial executer of fibrosis and scarring [23]. Of note, however, in zebrafish, we found these myofibroblast-like cells to display a strongly restricted spatial distribution within the granulation tissue, primarily located in deeper and marginal regions, close to skeletal muscle fibers adjacent to the wounds (Fig 5A,5D). In addition, and most strikingly, they, in contrast to myofibroblasts in mammalian wounds, lacked expression of fibrillar collagen- and FACIT-encoding genes (see below) (Figs 4F and 5A and S13) and did not show co-expression with other cluster 2 and cluster 4 marker genes (see below and S13 Fig), identifying them as a distinct cell population not participating in collagenous ECM production.

Yet, other fibroblasts of zebrafish wounds did display expression of genes marking fibroblast cell types implicated with mammalian fibrosis, for instance the Engrailed transcription factor gene *En1*. In mouse, En1 has been identified as a crucial pro-fibrotic factor that is highly up-regulated in a subset of wound fibroblasts upon skin injury, while ablation of these fibroblasts or blockage of En1 activation in them alleviated ECM production and prevented scarring [27,28]. In fish skin, the En1 ortholog *en1b* was expressed rather broadly throughout the fibroblast cluster, while in contrast to mouse, expression levels did not increase, but even slightly dropped upon wounding and during the different phases of wound healing (Figs 4G and S10), suggesting that this downregulation might possibly contribute to the different fibrotic response seen in zebrafish. However, an even broader expression within the fibroblast cluster, which actually was strongly up-regulated upon wounding, was also observed for multiple other pro-fibrotic factors, for instance the two paralogs of mammalian CTGF (Connective tissue growth factor; *ccn2a*, *ccn2b*; S10 Fig), a described central mediator of mammalian fibrosis required for the pro-fibrotic activity of TGFβ1 [77]; as well as for the two paralogs of mammalian Periostin (*postna*, *postnb*; S10 Fig), a secreted protein that has for instance been shown to be strongly up-regulated in myofibroblasts of the injured mouse heart, while deletion of periostin-positive myofibroblasts reduced collagen production and scar formation [78].

GO enrichment analysis of the different zebrafish fibroblast subclusters at 4 dpw revealed “ECM organization“ as a primary cellular function of clusters 0 and 3 and the wound-specific cluster 4 (Figs 4C, S9). Of note, the three clusters differed in the collagen types [79] they primarily generate. *col1a1* and c*ol1a2*, encoding collagen I, the main dermal fibril-forming collagen, was produced by all three clusters (Figs 4F and 5A,5B and S8 and S11), while *col5a2b*, encoding collagen V, another fibrillar collagen that serves as a nucleator of collagen I fibril formation, was primarily produced by cluster 3 (S8, S11 Figs), and *col12a1b*, encoding ColXII, a FACIT associated to collagen I-containing fibrils that has been implicated with pro-regenerative functions during zebrafish heart and spinal cord regeneration [47,80], by clusters 0 and 4 (Figs 4F and S8 and S11). Interestingly, cluster 0 also generated fibrillar collagen II (*col2a1b*) and fibrillar collagen XI (*col11a1b*) that serves as a nucleator of collagen II fibril formation (Figs 4F and S11). *col2a1b*-positive cells were rather uniformly distributed - and co-expressed with *col1a2* - throughout the granulation tissue, only sparing the upper marginal regions close to regenerating scales (Figs 5B and S13), where cluster 1 fibroblasts are located (see below). Such collagen II fibrils are normally mainly present in cartilage and have, at least to our knowledge, not been described in the context of mammalian cutaneous wound healing [42,81]; yet, administration of collagen type II isolated from sturgeon cartilage has been reported to improve the healing of cutaneous mouse wounds [82].

Cluster 3 was the only cluster that also displayed strong expression of *col10a1a* (S8, S11 Figs), encoding a network-forming collagen X that is mainly involved in hypertrophic cartilage and bone formation, endochondral ossification (involving the trans-differentiation of collagen type X-positive hypertrophic chondrocytes into osteoblasts), osteoarthritis and, together with type I and type II collagens, in bone fracture healing [83–85]. However, it has not been described in the context of skin wound healing. Yet, collagen X has been reported to serve as a ligand of the discoidin domain receptor DDR2 [86], and DDR2 has been shown to promote ECM remodeling and dermal wound healing in the mouse skin [87]. Strikingly, its expression is downregulated during stages of granulation tissue growth (2 dpw, 4 dpw; S11 Fig), pointing to a role during dermal homeostasis and/or regression, rather than growth. Consistently, *col10a1a*-positive cells were restricted to regions close to such collagen-free lacunae that most likely will be filled by subcutaneous adipose tissue (Figs 5C; compare with 3 and 5B)

Wound-specific cluster 4 was the only cluster also expressing *col18a1b* (S8, S11 Figs). It encodes collagen XVIII, a basement membrane proteoglycan that upon proteolytic cleavage for instance by matrix metalloproteases (MMPs) can give rise to endostatin, a matricryptin with multiple activities, including the blockage of MMP activation [88]. Mainly described in the context of angiogenesis and tumorigenesis, collagen XVIII and Endostatin have also been shown to have a negative impact on skin wound healing in mice [89], again in seeming contradiction to the increase of *col18a1b* expression levels during skin wound healing in zebrafish (S11 Fig). Within the zebrafish granulation tissue, *col18a1b*-positive cells were primarily found in deeper and more marginal regions, thus, not close to the regenerating epidermal-dermal basement membrane, but often associated with *acta1a*-positive myofibroblast-like cells in the vicinity of wound-adjacent skeletal muscle fibers (Figs 5D and S13). Finally, *col4a1/2*, encoding network-forming basement membrane collagen IV, was primarily expressed in clusters 0 and 4 (S11 Fig).

Similar to collagens, other ECM proteins were also generated by fibroblasts in a rather subcluster-specific manner. Genes encoding fibronectins (*fn1a/b*), which also form fibrils and like collagens can be cleaved by MMPs [90], were primarily expressed in clusters 0 and 4 (S12 Fig). In addition, clusters 0 and 4 displayed strong expression of Cartilage Intermediate Layer Protein (*cilp*; S12 Fig), a secreted ECM protein normally associated with bone and cartilage development, which also has been shown to bind TGFβ1, thereby suppressing TGFβ1-induced differentiation of fibroblasts to myofibroblasts during heart fibrosis [91]. Furthermore, cluster 0 was the only cluster displaying – in addition to *col2a1b* (see above; Fig 5B) - expression of osteoglycin (*ogna*; S12 Fig), a small leucine-rich proteoglycan (SLRP) that has been shown to be negatively regulated by TGFβ and to block myofibroblast differentiation and fibrosis in different mammalian tissues, including the heart [92], thus, like *cilp*, displaying clear anti-fibrotic effects. Consistently, *ogna*-positive cells were concentrated, together with *col10a1a*-positive cells of cluster 3, in the vicinity of the developing collagen-free lacunae (Fig 5C). Similarly, wound-specific cluster 4 specifically expressed plasminogen activator, urokinase (uPA) (*plaub*; S12 Fig), a secreted serine protease which, although widely upregulated in fibrotic diseases, has been shown to alleviate skin fibrosis [93].

ECM-degrading proteases displayed both subcluster-specific as well as more global fibroblast expression patterns. For example, the respectively secreted and membrane-tagged gelatinases *mmp2* and *mmp14b* [94], were broadly expressed in all fibroblast clusters, with higher levels at stages of granulation tissue regression (6 dpw) than at stages of granulation tissue formation, while the membrane-tagged disintegrin and metalloprotease 8 encoded by *adam8a* [95] was primarily expressed in clusters 0 and 4 (Figs 4F and S12). *mmp11b*, encoding a member of the stromelysin subfamily of MMPs, the mammalian ortholog of which is mainly implicated with carcinogenesis [96], but also with lung fibrosis [97] and foot ulcer [98], was expressed in parts of cluster 1 only and up-regulated at 4 dpw and 6 dpw (S10 Fig). Interestingly, these *mmp11b*-positive cells were exclusively localized at the base of re-growing scales, thus in cells that show *col1a2*, but lack *col2a1* expression (Figs 5E, S13), suggesting that they might represent scale-forming fibroblasts and might be involved in the regeneration of scales (which only contain type I, but not type II collagen [99]). In line with this notion, such scale-forming fibroblasts have been recently shown to express the transcription factor Twist2, and that *twist2* expression is up-regulated during and required for proper scale regeneration [100], while in other contexts (human osteoarthritis) TWIST1 has been shown to regulate the expression of MMP3, another member of the stromelysin subfamily [101].

Of the fibroblast subclusters with primary cellular functions other than “ECM organization“, cluster 2 was the only cluster with enriched “hydrogen peroxide (H_2_O_2_) catabolic/metabolic processes”. H_2_O_2_ has several and dose-dependent roles during wound healing. While disinfecting the wound tissue at relatively high concentrations, it promotes secretion of cytokines to improve dermal wound healing at lower concentrations [102,103]. Cluster 2 fibroblasts expressed high levels of *gstp1*, a member of the family of glutathione transferases which in other instances have been demonstrated to protect cells for example from lipid peroxidation [104], as well as *hbba1* (S12 Fig), a subunit of hemoglobin, which is known to attenuate H_2_O_2_-induced oxidative stress, intracellular glutathione depletion and cell death [105], thereby possibly alleviating negative effects of H_2_O_2_.

### The dynamics of ECM build up and breakdown

From our histology studies, we know that between 2 dpw and 4 dpw, the granulation tissue has in net grown, whereas between 4 dpw and 6 dpw, it has in net shrunk again (Fig 1B-1F). To analyze the impact of fibroblast-expressed genes on ECM build up and breakdown in a systematic and integrative manner, we used the Matrisome AnalyzeR tool [106] to annotate genes encoding ECM molecules, quantifying overall expression levels of selected genes in selected cell populations. We created two main categories: ECM build up, which comprises the categories of ECM-affiliated proteins, secreted factors, proteoglycans, collagens, and ECM glycoproteins; and ECM breakdown, which comprises ECM regulators (see Materials & Methods). Both ECM build up and ECM breakdown-related genes were upregulated upon wounding. However, in contrast to the actual increase of granulation tissue size from 2 dpw to 4 dpw, and its decrease from 4 dpw to 6 dpw, expression of build-up genes, if at all, continued to increase across all later time points, while expression of ECM breakdown even started to decrease after 4 dpw. This was not only so when integrating over the entire fibroblast population (Fig 4D,4E), but also for the individual fibroblast subclusters, with cluster 0 expressing most of these genes (S14 Fig).

That the dynamics of ECM build-up and breakdown scores of fibroblasts do not, in fact, match with the actual dynamics of granulation tissue formation and regression might indicate that other cell types such as innate immune cells have a major impact. For example, as detailed above, *mmp13a*, the major collagen type I collagenase, was strongly and almost exclusively expressed in neutrophils, while *timp2b*, encoding the major MMP13 tissue inhibitor [107], was strongly expressed in fibroblasts, neutrophils and macrophages, with macrophage levels dropping during phases of granulation tissue regression (4 dpw, 6 dpw; S7 Fig). In net, this might allow stronger extracellular collagen I degradation. In addition, as described above, *mcr1b*-expressing macrophages of subcluster 0 and *f13a1a*-expressing macrophages of subcluster 6 might be implicated in the phagocytosis and further intracellular degradation of MMP-predigested collagens.

Yet, Matrisome AnalyzeR analyses integrating fibroblast, macrophage and neutrophil clusters yielded results similar to those for the fibroblasts only, with ECM degradation/ ECM build-up ratios during granulation tissue regression (6 dpw) not being higher than during granulation tissue formation (2 dpw) (S14 Fig). Together, this suggests that ECM-forming and ECM-degrading processes largely co-exist during all stages of wound healing and that comparably moderate changes in the balance between the two - with little impact on the overall program assessed by Matrisome AnalyzeR – might in net gradually push the granulation tissue from growth to regression. However, we also want to point out that the results of our matrisome analyses need to be taken with some caution, as they have been acquired with mRNA data, and since in contrast to their mRNAs, the different ECM proteins themselves most likely have very different stabilities, with usually very high half-lives of collagens.

### Molecular interactions between macrophages and fibroblasts

In addition to directly contributing to ECM formation and degradation, innate immune cells mainly affect these processes by instructing fibroblasts accordingly. During the descriptions of the macrophage and fibroblast subclusters (see above), we have already referred to some examples of potential macrophage-fibroblast, macrophage-macrophage and/or fibroblast-fibroblast signaling processes, e.g., via IL1, TNFα and CXCL19/CXCR4. Other prominent signals known for decades to be involved in macrophage – fibroblast signaling during cutaneous wound healing in mammals are Fibroblast Growth Factors (FGFs), Platelet-Derived Growth Factor (PDGF), and Transforming Growth Factor beta (TGFβ) [108].

**Fig 6:**
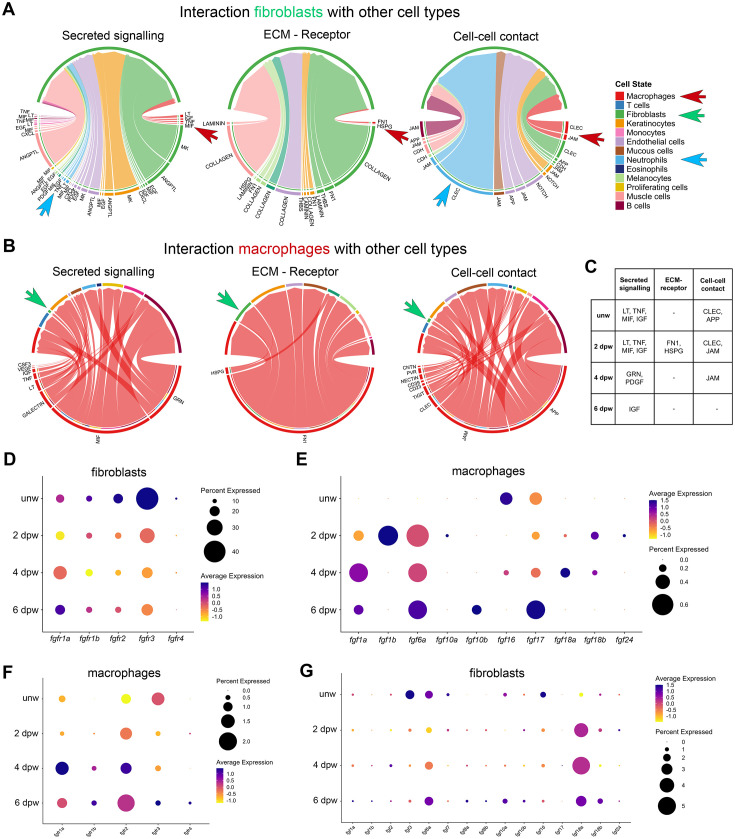
Ligand – receptor analysis between macrophages and fibroblasts. **(A)** Circos plots summarizing the active pathways of all cell clusters to fibroblasts during all investigated time points. **(B)** Circos plots summarizing the active pathways from macrophages to all cell clusters during all investigated time points. For corresponding Circos plots of specific time points, see S18 Fig. **(C)** Summary table of the interactions between macrophages and fibroblasts across the different time points. **(D-G)** Dot plots showing expression level of Fgf receptors and secreted Fgf ligands in macrophages and fibroblasts, as found in the scRNA-seq data. Transcripts of Fgf ligands not shown in (E) or (G) were not detected in macrophages or fibroblasts at any analyzed stage (0 dpw, 2 dpw, 4 dpw, 6 dpw).

Previous studies in our laboratory showed that global blockage of FGF signaling leads to failed granulation tissue formation similar to the effects of immune suppression, suggesting that innate immune cells might promote granulation tissue formation by secreting FGFs to stimulate fibroblasts [4]. To confirm these findings also in our scRNA-seq data, we checked the expression of Fgf ligands and Fgf receptors in fibroblasts and macrophages. All five receptors were expressed by dermal fibroblasts at different levels during wound healing (Fig 6D), while some of the secreted Fgfs were expressed by macrophages (Fig 6E). This indicates that FGF signaling from macrophages to fibroblasts might indeed lead to fibroblast activation and granulation tissue formation, as proposed [4]. However, FGF receptors were also expressed by macrophages (Fig 6F), and Fgf ligands by fibroblasts (Fig 6G), suggesting that fibroblasts might also stimulate each other and might talk back to macrophages via FGF signaling.

Similar expression patterns pointing to cross talk between multiple cells types were also observed for PDGF and TGFβ signaling. Thus, expression of the PDGF ligands (*pdgfab*, *pdgfba*) was not only initiated in macrophages upon wounding, but also in neutrophils and in fibroblasts themselves, while PDGF receptors (*pdgfra*, *pdgfrb*) were primarily and constitutively expressed in fibroblasts (S15 Fig). This suggests that PDGF signaling also occurs during wound healing in adult zebrafish, and that the described mitogenic as well as chemotactic effects on fibroblasts [109] might originate from different innate immune cells and might involve autocrine signaling among fibroblasts.

Similarly, the zebrafish TGFβ1 ligand *tgfb1a*, in addition to being induced in macrophages, was also constitutively expressed in fibroblasts themselves, while expression of its paralog *tgfb1b* was primarily induced in neutrophils. Furthermore, at least one of the TGFβ type 1 receptors (*tgfbr1a*, *tgfbr1b*) and the type 2 receptors (*tgfbr2a*, *tgfbr2b*) were induced or constitutively expressed in fibroblasts (and in macrophages and neutrophils) (S15 Fig), suggesting that the repair/fibrosis-stimulating effect of TGFβ signaling might in addition to the well-established instruction of fibroblasts by macrophages also involve instructions by neutrophils and by fibroblasts or among different fibroblast subclusters themselves.

To explore the potential interactions between macrophages and fibroblasts more systematically, we also performed CellChat analysis of our scRNA-seq data. This ligand receptor analysis was divided into three categories: secreted signaling, ECM – receptor and cell – cell contact, and analyzed for the interaction between all cell types and fibroblasts (Figs 6A and S18) and between macrophages and all cell types (Figs 6B and S18). Surprisingly, according to these analyses, compared to the interactions with other cell types, there is very little interaction of fibroblasts with neutrophils as well as with macrophages (Figs 6A and S16) and vice versa (Figs 6B and S16) via secreted signaling. Among such (low-level) secreted signaling was signaling via Lymphotoxins (LT), widely implicated in mammalian fibrosis [110], via TNF (see above) [111], via Macrophage migration Inhibitory Factor (MIF), long known for its central regulatory role during mammalian wound healing [112], via Insulin Growth Factor (IGF) signaling, known to be up-regulated in many mammalian fibrotic diseases, promoting fibroblast proliferation as well as ECM production [113], as well as via PDGF (see above) and progranulins (GRN), known mammalian inflammatory regulators with described pro- as well as anti-fibrotic effects [114] (Fig 6C).

Yet, relatively more interactions of macrophages with fibroblasts were predicted to occur via ECM-receptor contacts (Figs 6B and S16), and more interactions of fibroblasts with macrophages as well as with neutrophils via potential cell-cell contacts (Figs 6A and S16), the latter mainly via C-type lectin receptors (CLECs) and junction adhesion molecules (JAMs), both described with positive and negative implications in cell phagocytosis [115,116] (Fig 6C). Consistently, co-labeling of fibroblast-markers with macrophage or neutrophil markers, either using corresponding transgenic lines or HCR (see above), revealed close spatial associations between these cell types on sections through granulation tissues of 4 dpw wounds (S17 Fig).

### *plod2* is expressed by wound fibroblasts

We have also carried first functional studies of a factor found in our scRNA-seq studies which we had thought might make a difference between fibrotic wound healing in mammals and scar-free wound healing in zebrafish. A formerly reported factor mediating crucial interactions between macrophages and fibroblasts and thereby imperfect healing of cutaneous wounds in mice is the resistin-like molecule RELMα. It is encoded by the *Retnla* gene, produced and secreted by macrophages, and instructs fibroblasts to generate Lysylhydroxylase 2 (LH2) encoded by the *Plod2* gene. LH2 function in turn leads to tight DHLNL crosslinking of collagens and scar formation [39]. Interestingly, according to phylogenetic analyses *retnl* genes first show up at the level of Coelacanthiformes (Latimeria) and are absent in teleosts like the zebrafish [117], suggesting that during evolution they were first invented during the aquatic-terrestrial translocation of the ancestors of land-based vertebrates.

**Fig 7:**
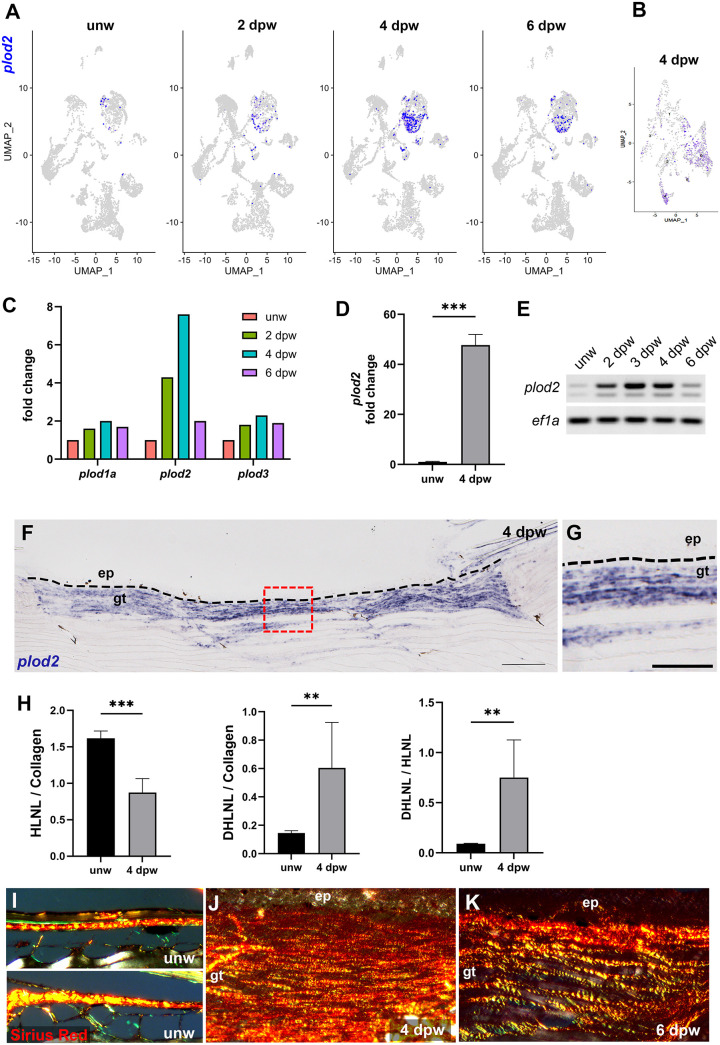
*plod2* expression is upregulated in cutaneous wounds of zebrafish. (A) UMAP representation of *plod2* expression in all cells and across all time points. (B) UMAP representation of *plod2* expression in fibroblasts at 4 dpw. (C) Fold change expression levels of *plod1a*, *plod2* and *plod3* across the time points compared to unwounded skin in scRNA-seq data. (D, E) Quantitative (D) and semi-quantitative (E) RT-PCR results demonstrate that *plod2* expression is upregulated upon wounding. Data in (D) represent mean  ±  SD, n  =  3 independent biological replicates. (F, G) In situ hybridization results showing *plod2* expression in the granulation tissue at 4 dpw. (H) Ratios of HLNL to collagen, DHLNL to collagen and DHLNL to HLNL crosslinks at 4 dpw. Data represent mean  ±  SD; n  =  6 for unw; n= = 2 for 4 dpw, with 3 pooled biopsies each. (I-K) Picosirius Red staining of sections through unwounded skin (I) and skin wounds at 4 dpw (J) and 6 dpw (K), visualized under linearly polarized light and after appropriate sample rotation/ orientation. Red (versus yellow or green) color indicates higher maturation/ crosslinking of collagen fibrils. Similar results were obtained in multiple sections of at least 3 independent wounds per condition. Numerical values for panels C, D, H, I and J can be found in S2 Data. Significances were determined with a two-tailed Student’s t test, *, P  <  0.05; **, P  <  0.01; ***, P  <  0.001. Abbreviations: ep, epidermis; gt, granulation tissues. Scale bars: 200 μm in F; 100 µm in G; 100 µm in K.

Nevertheless, we found *plod2* in our scRNA-seq analyses to be expressed in fibroblasts of unwounded skin, and to be strongly up-regulated upon wounding (Fig 7A), in particular in fibroblast subclusters 0 and 4 (Fig 7B), and at stages of granulation tissue formation (2 dpw, 4 dpw), while numbers of expressing cells and expression levels, in contrast to many other fibroblast-specific genes (S11, S12 Figs), dropped again during granulation tissue regression (6 dpw) (Fig 7A). Of note, zebrafish in addition to *plod2* has two other genes (*plod1a*, *plod3*) encoding lysyl hydroxylases, Lh1 and Lh3 [118]. However, according to our scRNA-seq data, *plod1a* and *plod3* expression levels were much less affected by wounding than that of *plod2* (Fig 7C), suggesting that the wounding-induced increase of collagen DHLNL crosslinking (see below) is largely caused by LH2. Furthermore, Lh2 has two splice variants, with the longer variant containing an additional exon (13A) of 63 base pairs that codes for the telopeptidyl lysyl hydroxylase activity crucial for DHLNL crosslink formation [118]. Strong *plod2* expression in zebrafish wounds was confirmed via *plod2* in situ hybridization at 4 dpw (Fig 7F,7G), while semiquantitative and quantitative RT-PCR of wound biopsies with specific primers for the longer splice isoform revealed that the expression of this relevant *plod2* variant was approximately 50-fold upregulated at 4 dpw compared to unwounded skin (Fig 7D), and had decreased again at 6 dpw (Fig 7E).

Since lysine residues in the telopeptide domain of collagens hydroxylated by LH2 will in subsequent steps give rise to DHLNL collagen crosslinks (rather than HLNL crosslinks when only one residue remains non-hydroxylated) [36], we also biochemically analyzed DHLNL and HLNL crosslinks in wound and unwounded skin biopsy samples. Indeed, upregulation of *plod2* in zebrafish wounds coincided with a reduction in HLNL/collagen ratios and an increase in DHLNL/collagen ratios, yielding an almost 5-fold increase in DHLNL/HLNL ratios at 4 dpw (~0.15 to ~0.75; Fig 7H). These numbers were similar to, and the changes upon wounding even stronger than those reported for mouse wounds (~0.3 to ~1.0) [39], indicating that in cutaneous zebrafish wounds, collagen crosslinking is as strong as in mouse wounds. Analyses of the birefringent properties of collagens via the picrosirius-polarization method [119] further revealed that despite this severalfold increase in DHLNL/HLNL ratios and collagen crosslinking, collagen fibers in the granulation tissue of 4 dpw zebrafish wounds were not as compact as in unwounded dermis (Fig 7I,7J), consistent with results obtained via transmission electron microscopy (see below/ Fig 9H in comparison to Fig 4 of reference [120]), but possibly more matured (indicated by the more red color of the fibers; Fig 7I,7J). Yet, at 6 dpw, when granulation tissue is regressing, collagen fiber density had dropped even further and collagen maturation/ crosslinking appeared reduced again (indicated by the yellow, rather than red color of stained fibers; Fig 7K). Together, this suggests that during granulation tissue regression, collagen crosslinking might actually be at least partly removed before collagens are fully degraded.

### *plod2* expression is stimulated by TGFβ signaling

**Fig 8:**
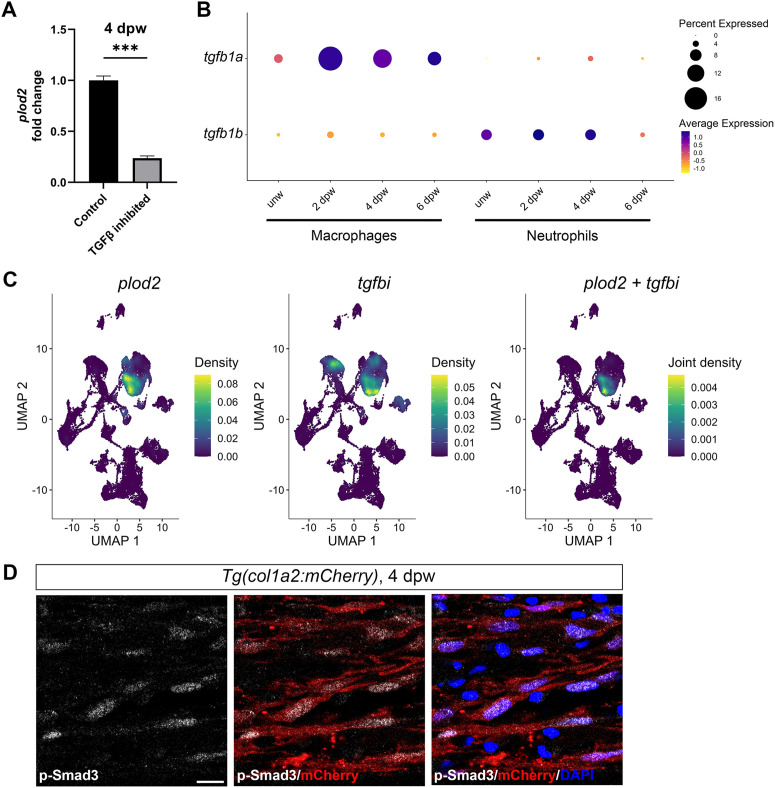
*plod2* expression is induced by TGFβ1 signaling. (A) qPCR results showing that *plod2* expression is downregulated at 4 dpw upon inhibition of TGFβ signaling via TGFβR inhibitor (SB431542). Data represent mean  ±  SD, n  =  3. Numerical values for panel A can be found in S2 Data. (B) Dot plot showing the expression of *tgfb1* paralogs in macrophages and neutrophils upon wounding. (C) UMAP representation of expression density for *plod2* and *tgfbi* and co-expression of both transcripts in scRNA-seq data. (D) Immunostaining of phospho-Smad3 and mCherry of the transgenic fibroblast reporter in sections of 4 dpw granulation tissue. Significances were determined with a two-tailed Student’s t test, *, P  <  0.05; **, P  <  0.01; ***, P  <  0.001. Scale bars: 10 μm in D.

Rather than by RELMα as in cutaneous wounds, *PLOD2* expression has in multiple other mammalian contexts been reported to be stimulated by TGFβ1/SMAD3 signaling [121–123]. Indeed, the promotor of the human *PLOD2* gene contains binding sites for the TGFβ1-activated transcription factor SMAD3, and binding of SMAD3 to this promotor element is required for TGFβ1-induced *PLOD2* expression in human fibroblasts, indicating that *PLOD2* is a direct TGFβ1/SMAD3 target gene [121]. To test whether in the absence of RELMα (see above), *plod2* expression in zebrafish cutaneous wounds might also be induced by TGFβ signaling, wounded fish were treated with the TGFβR inhibitor SB431542 between 3 dpw and 4 dpw. qPCR analysis revealed that at 4 dpw, *plod2* mRNA levels were 5-fold lower in samples of SB431542-treated fish than in the control group (Fig 8A), indicating that wounding-induced *plod2* expression depends on and occurs via TGFβ signaling. Consistently, according to our scRNA-seq data, both TGFβ1-encoding zebrafish genes, *tgfb1a* and *tgfb1b*, were upregulated upon wounding at 2 dpw, especially in neutrophils and macrophages (Fig 8B), pointing to these innate immune cells as a crucial source of TGFβ1 to stimulate fibroblasts. Yet, fibroblasts might in addition, and particular at later stages, also stimulate each other via *tgfb1a* expressed at increased levels in wound fibroblasts at 4 dpw (see above; S15 Fig). That wound fibroblasts receive and transmit such TGFβ signals is indicated by two lines of evidence. First, our scRNA-seq analysis revealed that expression of *tgfbi* (transforming growth factor beta induced), a known downstream target and marker for TGFβ signaling [124], was upregulated in fibroblasts (and in macrophages) at 4 dpw, and in the same fibroblast subclusters as *plod2* (Fig 8C). Second, immunostainings of phosphorylated Smad3 in a transgenic line with fluorescently labelled fibroblasts demonstrated the presence of activated Smad3 in the nuclei of wound fibroblasts at 4 dpw (Fig 8D). Taken together, the wound dermal fibroblasts in zebrafish also express *plod2* as they do in mammals, and this expression is dependent on TGFβ signaling, in contrast to mammalian dependency on RELMα.

### Gain or loss of Lh2 function does not alter regenerative capability

**Fig 9:**
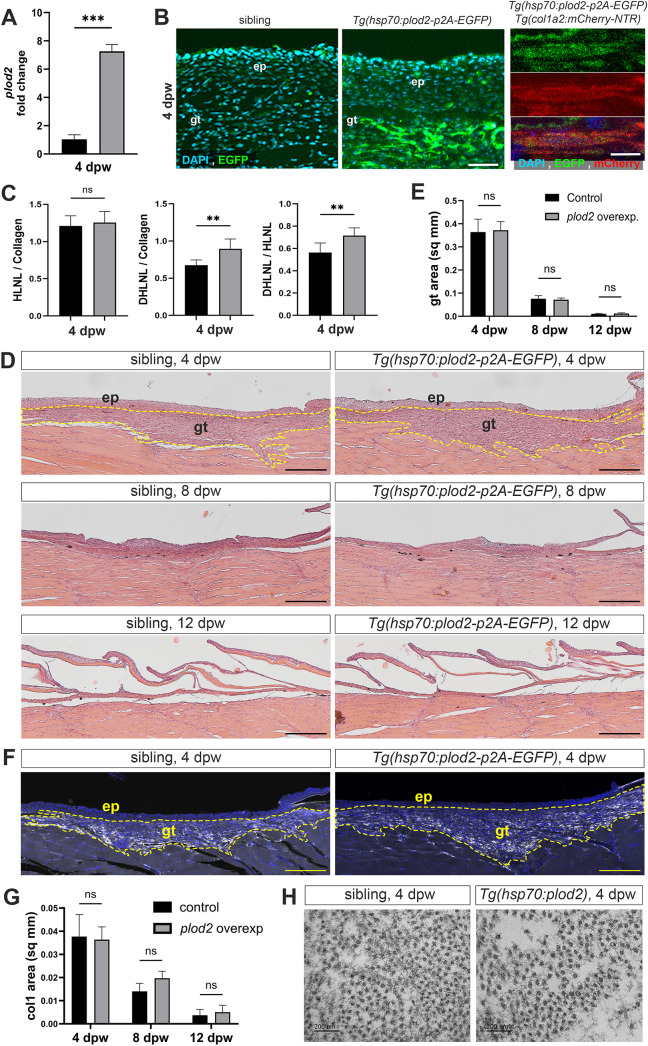
Gain of Lh2 function does not compromise granulation tissue resolution. (A) qPCR results showing that in from 0 – 4 dpw daily heat-shocked *Tg(hsp70l:plod2-p2A-EGFP)* fish, *plod2* expression is over sevenfold upregulated at 4 dpw compared to heat-shocked non-transgenic control siblings. Data represent mean ± SD, n = 3 biological replicates. **(B)** Anti-EGFP immunofluorescence, counterstained with DAPI, revealing *Tg(hsp70l:plod2-p2A-EGFP)*-encoded protein particularly in the granulation tissue of daily heat-shocked transgenic, but not of heat-shocked non-transgenic siblings, at 4 dpw. Right images show combined EGFP and mCherry fluorescences (single and merged channels) of a daily heat-shocked *Tg(hsp70l:plod2-p2A-EGFP)*, *Tg(col12a:mCherry-NTR)* double transgenic fish at 4 dpw and at higher magnification, directly revealing *Tg(hsp70l:plod2-p2A-EGFP)* expression (also) in fibroblasts of the granulation tissue labelled in red. **(C)** Ratios of HLNL to collagen, DHLNL to collagen and DHLNL to HLNL crosslinks in from 0-4 dpw daily heat-shocked transgenics and daily heat-shocked non-transgenic controls. Data represent mean ± SD; n = 6 for non-transgenics; n = 6 for transgenics. Compared to *plod2* transcript levels (~ 7-fold increase; **A)**, and DHLNL collagen crosslinking in wounded versus unwounded skin (~ 5-fold increase; Fig 5H), the increase of DHLNL collagen crosslinking in daily heat-shocked *Tg(hsp70l:plod2-p2A-EGFP)* transgenics compared to heat-shocked non-transgenic controls is rather moderate (~ 1.3-fold). This might point to an increasing contribution of crucial LH2 cofactors to limiting the rate of DHLNL formation under these particular conditions (increased *plod2* expression because of wounding AND transgene activation). One of such cofactors could be FKBP10/FKBP65, a chaperon colocalized with LH2 in the endoplasmic reticulum, which has been shown to potentiate the LH2-driven collagen HLNL-DHLNL crosslink switch [155], and mutations in which cause the same human disease as mutations in PLOD2 (osteogenesis imperfecta type XI, Bruck syndrome [156]). Of note, its zebrafish orthologs *fkbp10a* and *fkbp10b* are also expressed and upregulated in zebrafish skin fibroblasts (see S10 Fig.). (D) Representative H&E stainings on sections of from 0 – 4/8/12 dpw daily heat-shocked *Tg(hsp70l:plod2-p2A-EGFP)* fish and their control siblings at 4 dpw, 8 dpw and 12 dpw, respectively (yellow dashed lines mark the granulation tissue). (E) Quantification of granulation tissue sizes in sections as shown in (D), revealing that sizes were not changed upon forced *plod2* overexpression compared to wild-type siblings. Data represent mean ± SD, n = 3. (F) Representative images of immunostaining against collagen I protein (gray) at 4 dpw on paraffin sections, counterstained with DAPI (blue); for other stages, see S18 Fig. (G) Quantification of the collagen I-stained areas at 4 dpw, 8 dpw and 12 dpw, showing no changes upon forced *plod2* overexpression compared to wild-type siblings. Data represent mean ± SD, n = 3. Significances were determined with a two-tailed Student’s t test, *, P < 0.05; **, P < 0.01; ***, P < 0.001. (H) TEM analysis of *Tg(hsp70l:plod2-p2A-EGFP)* fish, demonstrating that the diameters and alignment of collagen fibers were not changed at 4 dpw compared to wild-type siblings. Numerical values for panels A, C, E and G can be found in S2 Data. Scale bars: 50 µm in left and medium images and 10 µm in right images of B; 200 μm in D, F; 200 nm in H. ep: epidermis, gt: granulation tissue.

Reduced *Plod2* expression levels caused by loss of RELMα in cutaneous mouse wounds coincides with reduced wound scarring. Similarly, direct siRNA-mediated knockdown of *Plod2* alleviates liver necrosis by making collagen fibers deposited by liver myofibroblast aligned in a more orderly manner and more easily degradable [125], pointing to a pro-fibrotic effect of Plod2. Yet, despite highly up-regulated *plod2* expression levels, zebrafish wounds eventually heal perfectly, with a full regression of the transiently formed granulation tissue. Given that in contrast to most other fibroblast markers, expression of *plod2* drops again during granulation tissue regression (see above; Fig 7A,7D), we investigated whether Plod2 might at least affect the dynamics or degree of granulation tissue formation and regression, performing gain- and loss-of-function experiments. For temporally controlled gain of function, a transgenic zebrafish line was generated carrying a heat shock inducible promoter driving the expression of *plod2*, *Tg(hsp70l:plod2-p2A-EGFP)*. For transgene activation, fish were daily heat-shocked, with the first heatshock directly before wounding at 0 dpw, and repeated heatshocks on every day until the fixation of fish for phenotypic analyses. Histology of sections of transgenic and non-transgenic control fish at 4 dpw, when granulation tissue is normally maximal, and at 8 dpw and 12 dpw, when granulation tissue has regressed, revealed that despite a several-fold increase of *plod2* expression in heat-shocked transgenic fish (Fig 9A), despite high levels of transgene-encoded protein particularly in wound fibroblasts of heat-shocked transgenics (Fig 9B) and despite an approximately 1.3 fold increase in the more stable collagen DHLNL crosslinking (Fig 9C), the sizes of granulation tissue were not changed upon forced *plod2* overexpression compared to heat-shocked non-transgenic siblings (Fig 9D,9E). Collagen I immunofluorescence and Transmission electron microscopy (TEM) further revealed that the amounts of fibrillar collagen type I (Figs 9F, 9G and S18), as well as the diameters and the alignment of collagen fibers (Fig 9H) within the granulation tissue were not affected by forced *plod2* overexpression compared to wild-type siblings.

For loss of function, we carried out the same type of analyses with a formerly isolated *plod2* mutant carrying a non-sense mutation that results in a C-terminally truncated (679 instead of 754 amino acid residues), non-functional protein [126]. Although displaying the described skeletal defects pointing to a requirement of LH2 for proper collagen I fiber organization in bones and for bone stability, and although displaying no compensatory expression of other *plod* genes in skin wounds (S19A Fig), but a reduction of collagen DHLNL crosslinks to approximately 50% of wild-type levels (S19B Fig), cutaneous wound healing was not affected in the mutants, with no significant difference in granulation tissue sizes compared to wild type siblings (S19C, S19D Fig), normal intensities and patterns of granulation tissue collagen I in immunostainings (S19E, S19F Fig), and no differences in the diameters and alignments of granulation tissue collagen fibers in TEM images (S19G Fig). Taken together, the overall regenerative ability was not altered during cutaneous wound healing in zebrafish upon forced gain or loss of Lh2 function and according changes in the crosslinking of granulation tissue collagens, indicating that even higher or lower levels of this pro-fibrotic factor and collagen crosslinking do not affect the dynamics of granulation tissue formation and regression.

## Discussion

Cutaneous wound healing represents a complex biological process which involves the interaction of diverse cell types and signaling pathways [5]. In contrast to fibrotic scarring observed in mammals, zebrafish possess the capability to perfectly restore lost or damaged tissue to its original state without scarring, although wounds also display “temporary fibrosis” in a granulation tissue that fully regresses during later stages of wound healing [4]. The same applies to other perfectly regenerating organs of adult zebrafish, for example the heart after cryoinjury, which also displays temporary formation of an excessive scar that later regresses [127]. In order to dissect the cellular and molecular processes underlying granulation tissue formation versus regression, we performed scRNA-seq for such different stages of cutaneous wound healing in adult zebrafish.

### Cellular and molecular dynamics and heterogeneities during zebrafish cutaneous wound healing

During mammalian skin wound healing, the wound microenvironment undergoes dynamic changes, with different cell types participating in tissue repair. Particularly, macrophages and fibroblasts are key players during this process [11,128], and this also seems to be so during zebrafish wound healing. Macrophages arrive in the wound before fibroblasts and later start to drop in numbers earlier than fibroblasts [4]. Yet, both cell types co-exist and cooperate not only during the formation, but also during the regression of granulation tissue.

As in mammals, macrophages and fibroblasts of zebrafish skin wounds exhibited a whole spectrum of activation states with unique gene expression profiles defining multiple subclusters – although most annotated subclusters still displayed substantial heterogeneity within themselves (S6, S13 Figs). Of note, only one of our annotated six fibroblast subclusters was wound-specific, compared to four of seven in cutaneous mouse wounds [24]. Macrophage subclusters with preferential pro-inflammatory, anti-inflammatory and/or pro-regenerative characteristics were identified. However, most of these subclusters co-existed during all stages of zebrafish wound healing, suggesting that pro- and anti-inflammatory processes largely overlap in time - although our calculated overall inflammation score clearly peaks at early stages of wound healing (Fig 2). A similar co-existence and temporal overlap during granulation tissue formation and regression was observed for most fibroblast subclusters, the main producers of the ECM (Fig 4). In addition to fibroblasts, innate immune cells also seem to directly contribute to ECM production - as also recently shown for mammalian wounds [67] - and ECM degradation. For example, macrophage subcluster 5 showed strong expression of genes encoding fibrillar collagens as well as basement membrane components (S5 Fig), while neutrophils showed strongest expression of *mmp13b*, together with *mmp13a* the only “professional” collagenase encoded by the zebrafish genome (S7 Fig). Of note, however, even when integrating fibroblasts, macrophages and neutrophils, our ECM build up/ ECM breakdown analyses yielded at first sight puzzling results, with formation/degradation ratios during granulation tissue formation not being higher than during granulation tissue regression (S14 Fig). Together, this points to a large degree of co-existence of ECM-forming and ECM-degrading processes during all stages of wound healing. In addition, it suggests that rather specific temporal or spatial changes in particular cell types and/or in the expression levels of rather few and particularly important factors might make the difference between net formation or net regression of granulation tissue.

Of note, in addition to changes in cells and potential regulatory genes, our scRNA-seq data also point to dynamic changes within the ECM itself. Thus, while Col I, the main fibrillar dermal collagen, was expressed at rather unaltered levels during all stages of wound healing, Col V, the other fibrillar dermal collagen, as well as Col II and Col X, fibrillar and network-forming collagens mainly present in (hypertrophic) cartilage, respectively, were downregulated during granulation tissue formation compared to unwounded skin, but strongly upregulated during granulation tissue regression. In contrast, Col XII, a FACIT associated with Col I-containing fibrils, Col XI, the other fibrillar collagen mainly present in cartilage, and Col XVIII were upregulated both during granulation tissue formation and regression (Figs 4G and S11). A strong upregulation particularly during granulation tissue regression was also observed for other ECM proteins, such as Fibronectin (*fn1b*), the Cartilage Intermediate Layer Protein 1 (*cilp1*) and the small leucine-rich proteoglycan Osteoglycin (*ogna*) (S12 Fig). Together, this points to crucial differences in the ECM composition of the growing versus the regressing granulation tissue, with the regressing ECM displaying striking similarities to cartilage ECM (Col II, Col X, Col XI, Cilp). Future studies have to address the mechanisms via which cartilage-like properties of the ECM might promote granulation tissue regression. However, our findings of *col10a1a*-positive fibroblasts being located close to (Fig 5), and *mrc1b*-positive macrophages being located within (Fig 3) forming lacunae that are eventually filled by adipose tissue point to the possible involvement of endochondral adipogenesis-like mechanisms, as occurring during zebrafish bone development [129], and as in line with the myofibroblast-adipocyte trans-differentiation reported for healing large skin wounds in mouse [130].

### Myofibroblast-like cells during zebrafish cutaneous wound healing

In mammals, myofibroblasts are considered as “the primary ECM-producing cell types executing fibrosis” [131], including scarring of skin wounds [23]. In addition, their contractile function contributes to the closure of mammalian skin wounds [132]. For the closure of zebrafish wounds, myofibroblasts are most likely dispensable, as the wounds close within hours and even before fibroblasts have entered the wound bed [4], purely driven by re-epithelialization movements of the epidermis [40]. Accordingly, we had thus far failed to detect myofibroblasts in zebrafish skin wounds with anti-smooth muscle actin antibodies, although myofibroblast-like cells have been described during zebrafish heart regeneration [127,133,134].

Interestingly, although not constituting a distinct fibroblast subcluster, we now identified skin wound-specific cells that display a transcriptional profile similar to mammalian myofibroblasts, with strong expression of genes most likely contributing to cellular contractility. However, compared to mammalian myofibroblasts and “regular” zebrafish fibroblast subtypes, they lack expression of collagen genes, suggesting that they might share at least some of the beneficial tissue-remodeling effects of mammalian myofibroblasts, while lacking their detrimental pro-fibrotic effects [22,23]. Future studies have to address the particular function of such myofibroblast-like cells in the zebrafish granulation tissue.

### Other pro-fibrotic pathways and Lh2 during zebrafish cutaneous wound healing

In mammalian wounds, other pro-fibrotic genes and cell types beyond myofibroblasts have been shown to contribute to persistent fibrosis and scarring. Puzzlingly, according to our scRNA-seq analysis, most of them are also present in zebrafish skin wounds, for instance En1, a transcription factor marking mammalian fibroblasts that upon ablation or blockage of En1 activation prevent scarring of injured skin [27,28], as well as Connective tissue growth factor (*cnn2a*, *cnn2b*; S10 Fig), a secreted growth factor cooperating with TGFβ1, inhibition of which can for instance reverse liver fibrosis [77], or Periostin (*postna*, *postnb*; S10 Fig), a secreted protein made by a specific fibroblast subcluster, ablation of which alleviates scarring in injured hearts [78]. Future cell ablation and genetic gain- and loss-of-function experiments have to reveal the roles of these cell types and genes during cutaneous wound healing in zebrafish.

Another described mammalian pro-fibrotic factor, for which we have already here performed functional studies, is Lysylhydroxylase 2 (Lh2), encoded by the *Plod2* gene. In mammalian wounds, *Plod2* expression is induced in dermal fibroblasts by RELMα released by macrophages, leading to more stable DHLNL-dependent crosslinking between collagen fibers and persistent fibrosis [39]. Given that *retln* genes encoding RELMα and related factors are absent from fish genomes, we had speculated that zebrafish wounds might lack *plod2* expression and collagen DHLNL crosslinks, and that this has a crucial impact on granulation tissue regression. Surprisingly, however, our results demonstrated that *plod2* expression and collagen DHLNL crosslinks are also induced in zebrafish wounds, in this case induced by TGFβ signaling, a well-known mediator of mammalian fibrosis in other pathways and contexts (Figs 7 and 8). Moreover, Lh2 gain or loss of function did not alter the regenerative ability of zebrafish (Figs 9 and S19), challenging this particular conception of fibrosis regulation [39], and pointing to the existence of alternative – or at least additional - mechanisms allowing granulation tissue regression, ECM breakdown and thereby scar-free wound healing even in the presence of those more stable collagen fibrils. Yet, our data do not fully exclude a general contribution of collagen cross-linking to the kinetics of granulation tissue formation and regression. Thus, collagens can also be crosslinked via other molecular, for instance transglutaminase-mediated, mechanisms [135]. In addition, even a contribution of lysylhydroxylase-mediated mechanisms cannot be ruled out completely, as the effect of Lh2 overexpression could be limited by rate-limiting concentrations of crucial co-factors like Fkbp10 (see legend of Fig 9), and the effect of loss of Lh2 could be limited by partial functional redundancies with low levels of Lh1 and Lh3 (Figs 7 and S19).

### Implications for wound healing therapies

The long-term goal of our work is the identification of specific cell types or genes making the difference between perfect healing with only temporary fibrosis in fish versus imperfect healing with persistent fibrosis and scarring in mammals, including human. In principle, we would expect that fish wounds contain anti-fibrotic factors absent in mouse wounds, and/or that mouse wounds contain pro-fibrotic factors absent in fish wounds. In this light, RELMα described above was a good candidate for the second category, which unfortunately turned out negative, as its absence in fish can be compensated by TGFβ. This makes it quite likely that similar compensatory mechanisms could also be activated when targeting RELMα in human skins to alleviate scarring. Thus, concomitant targeting of RELMα and TGFβ signaling might be a reasonable approach. Candidates for anti-fibrotic factors absent in mouse wounds were not so apparent in our analyses. They might be among the genes with no annotated function as yet that were strongly up-regulated in zebrafish wounds, for instance si:dkey-21e2.8, which is strongly expressed in fibroblast subcluster 3 of fish wounds; S8 Fig). Yet, also our own BLAST searches did not unravel any orthologs in vertebrate classes other than fish, thus it remains unclear whether it is a least part of a pathway shared between fish and mammals, and how to possibly pharmacologically promote this pathway in human wounds.

Alternatively, or in addition, rather than the presence versus complete absence of already known pro- and anti-fibrotic factors, more subtle differences in the relative abundances of such factors in both fish and mammalian skin wounds might underlie perfect versus imperfect wound healing in fish versus mammals. If so, it might on one hand be more challenging to identify such most likely multi-factorial differences in cross-species scRNA-seq analyses [136]. On the other hand, however, when successful, it might be instrumental for the rather straight-forward development of novel or modified therapeutic interventions in wound healing-related human disorders, possibly using a combination of already established pharmacological agonists and antagonists.

In conclusion, our findings set the base for further and more systematic cross-species scRNA-seq analyses to elucidate crucial differences in the cellular and molecular dynamics during the different phases of perfect cutaneous wound healing in fish versus fibrotic wound healing in mammals, with the ultimate goal to develop new preventative and reparative therapies for skin repair in human.

## Materials and methods

### Ethics statement

The zebrafish studies were approved by the animal welfare office of the state of Northrhine-Westfalia (LANUV), approval numbers: 81-02.04.2018.A097, 81-02.04.2022.A104, 81-02.04.40.2022.VG005.

### Zebrafish lines and wounding

Six to eight months of age adult zebrafish Tüpfel long fin (TL) and Ekkwill (EK) wild-type strains were used. Transgenic and mutant lines used were: *Tg(col1a2:mCherry-NTR)*^cn11Tg^ [137], *Tg(mpx:GFP)*^i114Tg^ [138], *Tg(lyz:EGFP)*^nz117Tg^ [139], *Tg(mpeg:EGFP)*^gl22Tg^ [140], *Tg(mpeg:mCherry)*^gl23Tg^ [140], *TgBAC(tnfa:GFP)*^pd1028Tg^ [62], *plod2*^sa1768^ [141]. Transgenic lines *Tg(hsp70l:plod2-p2A-egfp)*^fr62Tg^ and *Tgki(col1a2:GFP-Col1a2)*^fr64Tg^ were generated in this study. Full-thickness wounds of adult fish with a diameter of ∼3 mm were introduced with a clinical dermatology laser as described [4].

Zebrafish were raised at 28^o^C on 14 hours/10 hours light/dark cycle and fed with paramecia, dry flake food and live/frozen artemia daily. Feeding of larvae and adult fish and monitoring of the quality of the system water were done by the institute’s animal care takers daily.

The zebrafish wound healing experiments were approved by the local and federal animal care committees (City of Cologne: 8.87-50.10.31.08.129; LANUV Nordrhein-Westfalen: 81-02.04.2018.A097, 81-02.04.2022.A104, 81-02.04.40.2022.VG005).

### Genotyping of *plod2* mutants

*plod2*^sa1768^ mutants were genotyped using PCR amplification with the primers listed in Table 1. This yields a 264 bp amplification product which upon digestion with *Mbo*II enzyme (New England Biolabs) yields bands of 153 bp, 70 bp and 41 bp in wild type genomic DNA, but bands of 223 bp and 41 bp in mutant genomic DNA.

**Table 1:** Primer sequences used in this study.

Primer ID	Sequence	Comments
plod2_fw	5’- GAATCACAAGTAAATCAGACACA -3’	genotyping
plod2_rev	5’- AGTTAGGACATGGTGTAAGATG -3’	
plod2b_fw	5’- AAAAAGATATCCACCATGGAGCGGCGTCGG -3’	transgenesis
plod2b_rev	5’- AAAGCGGCCGCGGGATCTACGAAAGACACTGCTATG T -3’	
p2A-egfp_fw	5’- AAAGCGGCCGCAGCTACTAACTTCAGCCTGCTGAAG CAGGCTGGCGACGTGGAGGAGAACCCTGGACCTGGTA TGGTGAGCAAGGGCGAG -3’	
egfp_rev	5’- AAAAAAGCTTTTACTTGTACAGCTCGTCCATGCC -3’	
ef1alpha_fw	5’- TGGTACTTCTCAGGCTGACT -3’	qPCR
ef1alpha_rev	5’- CTCCAACGATCAGCTGTTTCAC -3’	
rps11a_fw	5’- GATGGCGGACACTCAGAAC -3’	
rps11a_rev	5’- CCAATCCAACGTTTCTGTGA -3’	
col1a2_fw	5’- CAAGGAGTCTGCATGTCGGT -3’	
col1a2_rev	5’- ATCAGGTCCCTTAGGACCCC -3’	
plod2b_fw	5’- CATTCCATATGCTCAGATCCCCA -3’	
plod2b_rev	5’- TCTCCAGTCCAGAGGATTTTCG -3’	
plod1a_fw	5‘TAAGACCACATCACGACGCC-3‘	
plod1a_rev	5‘-GCCTCCACCCTGAAAGTCAA-3‘	
plod3_fw	5‘-ACGCTGAAATTCCTGCCGTC-3‘	
plod3_rev	5‘-AGTTCAGTTTTCCCGTCGCT-3‘	
plod2-fw	5’- AGTGTGGAACATTCCTTTCCTGGC -3’	RT-PCR
plod2-rev	5’- GAGAAGTGTTGTAGTTGGCAGTGG -3’	
5’HA_fw	5’- GGTCTGTCCCAGTTGTTTGATTATATTGT -3’	knock-in
5’HA_rev	5’-CTGCTTCAGCAGGGAGAAGTTGGTGGCGAAGTTCTACAAAAAGC -3’	
p2A_fw	5’- GCCACCAACTTCTCCCTGCTGAAGCAGGCCGGCGACGTGGAGG AGAACCCCGGCCCCATGCTCAGCTTTGTGGATACCC -3’	
egfp_rev	5’- CCAGGATCGGGACCCTTGGCACCGTCGTATTGGGATCCCTTGTA CAGCTCGTCC -3’	
3’HA_fw	5’- CAATACGACGGTGCCAAGGGTCCCGATCCTGGCCCTGGACCTATG GTGAG -3’	
3’HA_rev	5’- GCCACGAGCACCAATAGCT -3’	
crRNA	5’- GGCGCTAAAGGACCTGACCC -3’	

### Plasmid construction and transgenesis

For cloning of the construct hsp70l:plod2-p2A-egfp, the hsp70l promoter region was digested with *Xba*I and *Eco*RV restriction enzymes from a pBSIISK-hsp70l plasmid. Zebrafish *plod2* ORF was amplified using primers listed in Table 1 and digested with *Eco*RV and *Not*I restriction enzymes. The egfp-polyA part was amplified from p3E-egfp-ployA plasmid (Tol2 kit; [142]), with the p2A part added via the primers during amplification, followed by digestion of the PCR product with *Not*I and *Hind*III restriction enzymes. Finally, pminiTol2 plasmid was digested with XbaI and HindIII restriction enzymes and ligation was done between linearized pminiTol2 plasmid backbone and all three inserts together. The final construct was checked with Sanger sequencing. The final plasmid construct was microinjected into fertilized 1-cell-stage embryos using micro-manipulator. The final concentrations of the constructs in the injection mix were 25 ng/μl together with 25 ng/μl Tol2 mRNA and 5 nl of the solution mix was injected inside of the cell. Embryos were raised and transgene expression was checked with fluorescent markers.

For the *Tgki(col1a2:GFP-col1a2)*^fr64Tg^ knock-in line, we constructed a DNA recombination template that upon recombination with the endogenous *col1a2* locus encodes a GFP-Col1a fusion protein with GFP located N-terminal of the collagen’s telopeptide domain, exactly as formerly reported for a zebrafish transgene with a keratinocyte-specific promoter [143]. The constructed DNA template consisted of a 5’ homology arm (633 bp), p2A self-cleaving peptide sequence, signal peptide sequence, EGFP coding sequence, and a 3’ homology arm (701 bp). The construct was cloned into the pGEM-T Easy vector and subsequently isolated as a linear DNA fragment via restriction digestion for microinjection. To facilitate site-specific integration, a two-component guide RNA system was used, consisting of a crRNA targeting the *col1a2* locus near the telopeptide domain and a universal tracrRNA. The crRNA and tracrRNA were annealed in equal amounts of 30 μM each in Nuclease-Free Duplex Buffer (IDT) by heating to 95°C for 5 minutes and subsequent cooling to room temperature to form a functional duplex. 9 μM of complexed RNA was injected together with 150 ng/μl of Cas9 mRNA and 20 ng/µl linear donor fragment into one-cell stage zebrafish embryos. Injected embryos were screened for mosaic EGFP expression to confirm successful delivery. Potential founders were raised to adulthood and screened for germline transmission. The isolated stable line fr64tg was checked for proper *col1a2* recombination via PCR of genomic DNA and Sanger sequencing. It is maintained in the heterozygous status. Phenotypically, heterozygous fish were wild-type by all means, and displayed completely normal cutaneous wound healing.

### Tissue labelling procedures

For paraffin embedding, samples were fixed in 4% paraformaldehyde/phosphate buffered saline (PBS) overnight at room temperature, decalcified in 0.5M EDTA (pH 8.0) for 5 days, dehydrated in a graded series of alcohols, cleared in Roti-Histol (Carl Roth, Karlsruhe, Germany), and embedded in paraffin. For cryosectioning, the trunk of the fish was cut in smaller pieces and fixed with 4% PFA/PBS overnight at 4^o^C. Fixed samples were washed with PBS, decalcified in 0.5M EDTA (pH 8.0) for 3 days at 4^o^C. Afterwards, the samples were incubated with 30% sucrose/PBS solution for two days at 4^o^C before embedding in OCT embedding matrix (Carl Roth, Germany). Both paraffin and OCT embedded blocks were sectioned at 10 μm thickness.

Picosirius Red stainings of cryosections were performed as previously described [144,145]. Briefly, 0.5 g Direct Red 80 (Sigma-Aldrich, Catalog # 365548) were mixed with 500 ml of picric acid (Sigma-Aldrich, Catalog # P6744). Sections were incubated in the Picrosirius Red solution for 60 minutes at room temperature and were afterwards washed with acidified H_2_O and were dehydrated with EtOH. Slides were mounted with Entellan (VWR International GmbH, Catalog # 1079600500). Stained tissue was imaged using linear polarized light on a Zeiss Axioplan 2 (Carl Zeiss AG, Oberkochen, Germany).

Immunofluorescence and histological analyses were performed using standard protocols. Antigen retrieval step was performed additionally for immunofluorescence analysis with 10 mM citrate buffer (pH 6.0) for 15 minutes. Stainings were performed with the primary antibodies mouse anti-mCherry (1:400, Abcam, ab125096), rabbit anti-collagen I (1:200, Abcam, ab23730), rabbit anti-phospho-Smad3 (1:400, Abcam, ab52903), chicken anti-GFP (1:500, Invitrogen, A-10262).

In situ hybridization on cryosections was performed as previously described [146]. Briefly, tissue sections were fixed again 5 min with 4% PFA, treated with proteinase K (5 μg/ml, 30 min) and PBS washes. Slides were incubated in triethanolamine-acetic anhydrate pH 8.0 5 min, then dehydrated in a series of EtOH. Sections were incubated for 3 h with hybridization solution (50% formamide, 5X SSC, 1X Denhardt, 10% dextran sulfate, 1 mg/ml tRNA). Digoxigenin-labeled *plod2* probe was generated by in vitro transcription [118]. Slides were blocked with 10% sheep serum in PBST at room temperature for 3 h, incubated overnight at 4°C with alkaline phosphatase-labeled anti-DIG antibody (Roche) and enzymatic activity was detected with BCIP and NBT.

Hybridization Chain Reaction (HCR) analyses on cryosections were performed as previously described [147]. Briefly, tissue was permeabilized with 10 μg/ml proteinase K in TBS at 37°C for 15 minutes. Slides were washed with TBS-Tween20 (0,1%) and further incubated with 0.2 N HCl for 20 minutes at room temperature. Slides were then incubated with triethanolamine and acetic acid for 5 minutes at room temperature. Afterwards the slides were washed, pre-hybridized with hybridization buffer at 65°C and then incubated with 1.6 pmol per probe in hybridization buffer over night at 45°C. On the next day the slides were washed with a dilution series of probe wash buffer and 5X SSC-Tween20 (0,1%) ending on 100% SSC-T. Wash steps were performed at 45°C. Slides were incubated with amplification buffer for 2 hours at room temperature and were afterwards incubated with hair pin solution (hair pin pair in amplification buffer) over night at room temperature. The slides were then washed with 5x SSC-T and mounted with MOWIOL containing DAPI (1:1000). HCR probes were ordered at IDT DNA Technologies (probe sequences available upon request)

### Drug treatments and heat shock induction

TGFβR inhibitor (SB431542, Sigma-Aldrich, S4317) was administered to adult zebrafish according to the indicated times in E2 medium with 50 μM final concentration. All drug treatments were done with a final concentration of 0.1% DMSO. Drug-containing E2 media were changed every 24 hours as necessary.

Heat induction for adult zebrafish was done by transferring them to pre-heated 38°C system water. Adult fish were kept in a 38°C incubator for 2 hours before transferring them back to the system.

### qPCR analysis

Adult zebrafish wound tissue samples were collected using a biopsy punch with a diameter of 4 mm. Collected tissues for total RNA extraction were snap frozen with dry ice before adding Trizol reagent. Tissue samples were homogenized and kept at -80^o^C for at least overnight until total RNA extraction was done according to the Monarch Total RNA Miniprep Kit protocol. cDNA synthesis was done with 1 μg of RNA with the iScript cDNA Synthesis Kit. The qPCR for gene expression analysis was performed with a 7500 Fast Real-Time PCR System (Applied Biosystems) using the SYBR Green PCR Master Mix and the primers listed in Table 1 (including *rps11a* and *ef1alpha* as “house-keeping” control genes). Primer efficiency was determined by the thermocycler software via the slope of the fluorescence curve in the logarithmic phase, and/ or by a dilution series of sibling control cDNA from 20 ng to 0.2 pg, and was always above 0.9. Specificity was determined by the thermocycler after the final amplification step via melting curve analysis (revealing always only one peak/ one amplification product). Fold change was calculated using the ΔΔC_T_ method.

### Transmission electron microscopy

For TEM analysis, the trunk of adult zebrafish was cut in smaller pieces and fixed overnight at 4^o^C with 2% PFA + 2% GA solution. Fixed samples were washed with PBS, decalcified in 0.5 M EDTA (pH 8.0) for 3 days at 4^o^C. Afterwards, samples of 1–2 mm thickness were incubated 0.1M cacodylic acid and re-fixed with 2% osmium tetroxide (Science Services) in 0.1M cacodylic acid, and washed 4 times for 15 min in 0.1M cacodylic acid. Through a series of ethanol 50–100%, a mixture ethanol/ propylene oxide and 100% propylene oxide, the tissue was then embedded in EPON (Science Services). After embedding, the tissue was cut in 70 nm sections with a ultramicrotome (UC6, Leica) on a grid. Grids with sections were contrasted with 1.5% uranyl acetate aqueous solution for 15 min at 37°C, incubated for 4 min in lead citrate solution (Leica Ultrastain II), washed again five times in water and dried on a filter paper.

Images were acquired with a transmission electron microscope (JEM 2100 Plus, JEOL), a OneView 4K camera (Gatan) with DigitalMicrograph software at 80 KV at room temperature.

### Crosslink analysis

Collagen crosslink analysis of uninjured skin and three pooled biopsies of cutaneous wounds per stage was performed as previously described [39,148]. Briefly, tissue specimens were reduced, denatured, and digested, and analysis was performed with an amino acid analyzer (Biochrom 20).

### Preparation of single cell suspensions

For single cell RNA sequencing, single cell suspensions were prepared. For this task, unwounded skin tissue from three adult zebrafish was collected by scraping off the skin and pooling in PBS (Mg^2+^ and Ca^2+^-free). In addition, the wound tissues for 2 dpw, 4 dpw and 6 dpw were collected using a biopsy punch with a diameter of 4 mm from three adult zebrafish each. PBS was replaced with 0.25 mg/ml Liberase (Roche) in PBS solution and the tissues were incubated at 30^o^C for an hour with slow agitation (~300 rpm). The solution was pipetted every 10–15 minutes. After successful digestion of the tissue, the solution was mixed with 5% FBS/PBS and filtered through a cell strainer with 70 μm nylon mesh and then a cell strainer with 40 μm nylon mesh in 50 ml Falcon tubes. The falcon tubes were centrifuged at 500 x g for 5 minutes and the pellet was resuspended with 2% FBS/PBS. The viability of the cells were more than ninety percent. The single cell suspensions were kept on ice until they were processed at the Cologne Center for Genomics, University of Cologne. 10.000 cells per condition was aimed for single cell RNA sequencing.

### Clustering of single cell data matrix

Single cell suspensions were processed, sequenced and single cell library construction was mapped to the zebrafish transcriptome by Cologne Center for Genomics, University of Cologne using 10x Genomics 3’scRNA seq application (Version 3’v.3.1) with Cell Ranger software used to generate feature-barcode matrices. Single cell RNA sequencing data was analyzed using RStudio program with Seurat (V4.3.0) software package for R using standard quality control [42,149–152], normalization, integration and analysis steps. Low quality cells were excluded from downstream analysis according to following criteria for retaining cells: 500 < nUMI < 30.000, 200 < nGene < 4500, mito < 25%. The top 30 most differentially expressed genes were identified for each cluster and the most likely cell type was annotated for each cluster according to expression patterns of the genes found in public databases.

### Gene ontology (GO) enrichment analysis

To analyze and visualize functional profiles of differentially expressed gene clusters across cell subtypes, we applied clusterProfiler R package [153] as indicated in accessible vignette (https://yulab-smu.github.io/clusterProfiler-book/). GO enrichment analysis for biological processes was performed using the list of significantly upregulated genes. GO terms with adjusted p-value < 0.05 were considered significantly enriched.

### STRING analysis

Overexpressed genes in the macrophage population of the 2 dpw sample (261 genes) were compared with the unwounded sample. The gene list was queried in STRING (version 11.5) using the Multiple Proteins by Names/Identifiers function. Functional enrichment analysis was performed under Biological Process (Gene Ontology) to identify pathways associated with inflammation. Among the enriched pathways, “GO:0006954 – Inflammatory response” was identified.

### Extracellular matrix (ECM) analysis

We used Matrisome AnalyzeR package to characterize ECM composition at single-cell resolution [106]. Differential gene expression analysis was first performed by comparing each sample to the unwounded sample. Genes significantly overexpressed in each sample were selected for downstream ECM-specific annotation following scRNA-seq analysis for Seurat objects provided workflow (https://github.com/Matrisome/MatrisomeAnalyzeR).

### Cell–cell communication analysis

CellChat R package [154] was employed to determine potential ligand-receptor communication in our scRNA-seq gene expression data by using CellChatDB.zebrafish, according to the available tutorial (https://github.com/sqjin/CellChat).

### Quantification and statistical analysis

For the measurement of the granulation tissue areas, FIJI-ImageJ software was used and the area was drawn manually. For the quantification of collagen-containing areas (Figs 7, S19), immunofluorescence images were acquired using an Apotome Imager.Z1 microscope. Image analysis was performed using ImageJ/Fiji software. To quantify collagen deposition, the granulation tissue was defined as the Region of Interest (ROI). Images were converted to 8-bit grayscale, and a threshold was manually established based on the background intensity of the negative control sections to identify collagen-positive pixels. This threshold was then applied uniformly across all images to create a binary mask. The total collagen-positive area was calculated within the ROI. Additionally, the total area of the granulation tissue was measured for each sample; as no significant differences in total granulation area were observed between wild-type controls, transgenics and mutants of corresponding stages, collagen-containing areas are reported with absolute values (in mm^2^). Statistical analysis was performed using GraphPad Prism software. A two-tailed Student’s t test was performed for comparison of two groups. Significances were: *, P < 0.05; **, P < 0.01; ***, P < 0.001.

## Supporting information

S1 Fig UMAP representation of single cell RNA sequencing data after integration of four datasets and cell clustering results across the time points. (PDF)

S2 Fig Identification of different cell clusters during zebrafish wound healing. (PDF)

S3 Fig Identification of macrophage subclusters. (PDF)

S4 Fig GO analysis of biological processes of macrophage subclusters in unwounded skin and at 4 dpw and 6 dpw. (PDF)

S5 Fig UMAP representations of selected macrophage-specific genes across the different stages of wound healing: mrc1b, cxcl19, col1a1a, col4a1, col4a2, lama4, f13a1a, esr2a, cpn1, tnfa, tgfb1a, cxcr4a, cxcr4b, il10. (PDF)

S6 Fig Double UMAP representations of selected pairs of macrophage marker genes. (PDF)

S7 Fig Potential other contributions of innate immune cells to ECM breakdown: mmp13b, timp2b. (PDF)

S8 Fig Identification of fibroblast subclusters. (PDF)

S9 Fig GO analysis of biological processes of fibroblast subclusters in unwounded skin and at 2 dpw and 6 dpw. (PDF)

S10 Fig UMAP representations of myofibroblast-like-specific genes and for other fibroblast genes implicated in fibrosis across different stages of wound healing: acta1a, ankrd1a, desma, cdh15, en1b, postna, postnb, cnn2a, cnn2b, fkbp10a, fkbp10b. (PDF)

S11 Fig UMAP representations of selected collagen-encoding genes across the different stages of wound healing: col1a1a, col1a2, col2a1b, col5a2b, col12a1b, col11a1b, col10a1a, col10a1b, col18a1b, col4a1. (PDF)

S12 Fig UMAP representations of selected fibroblast-specific genes across the different stages of wound healing: fn1b, cilp, ogna, plaub, mmp2, mmp10b, adam8a, hbba1. (PDF)

S13 Fig Double UMAP representations of selected pairs of fibroblast marker genes. (PDF)

S14 Fig Violin plots showing the expression levels of ECM build-up and ECM breakdown-related genes in different fibroblast subclusters as well as in all fibroblasts, macrophages and neutrophils together, in unwounded skin and across the different phases of cutaneous wound healing. (PDF)

S15 Fig Expression of PDGF and TGFβ1 ligands and receptors in macrophages, neutrophils and fibroblasts. (PDF)

S16 Fig Ligand – receptor analysis between macrophages and fibroblasts. (PDF)

S17 Fig Fibroblasts are spatially associated with neutrophils and macrophages. (PDF)

S18 Fig Gain of Lh2 function does not compromise granulation tissue resolution. (PDF)

S19 Fig Genetic loss of Lh2 function does not compromise granulation tissue formation and resolution. (PDF)

S1 Data Scripts for scRNAseq data evaluation. (ZIP)

S2 Data Numerical values for panels in Figs 1G, 2B, 3B, 5C, 5D, 5H, 5I, 5J, 6A, 7A, 7C, 7E, S19B and S19D. (XLSX)
