# Supplementary material for: Exploring mechanisms of scar-free skin wound healing in adult zebrafish in comparison to mouse
Source: PLoS Genet. 2026 Jun 24;22(6):e1012200. doi: 10.1371/journal.pgen.1012200 (PMC13322528; doi:10.1371/journal.pgen.1012200)

S1 Fig. Cellular composition of zebrafish cutaneous wounds. UMAP representation of single cell RNA sequencing data after integration of four datasets and cell clustering results across the time points.

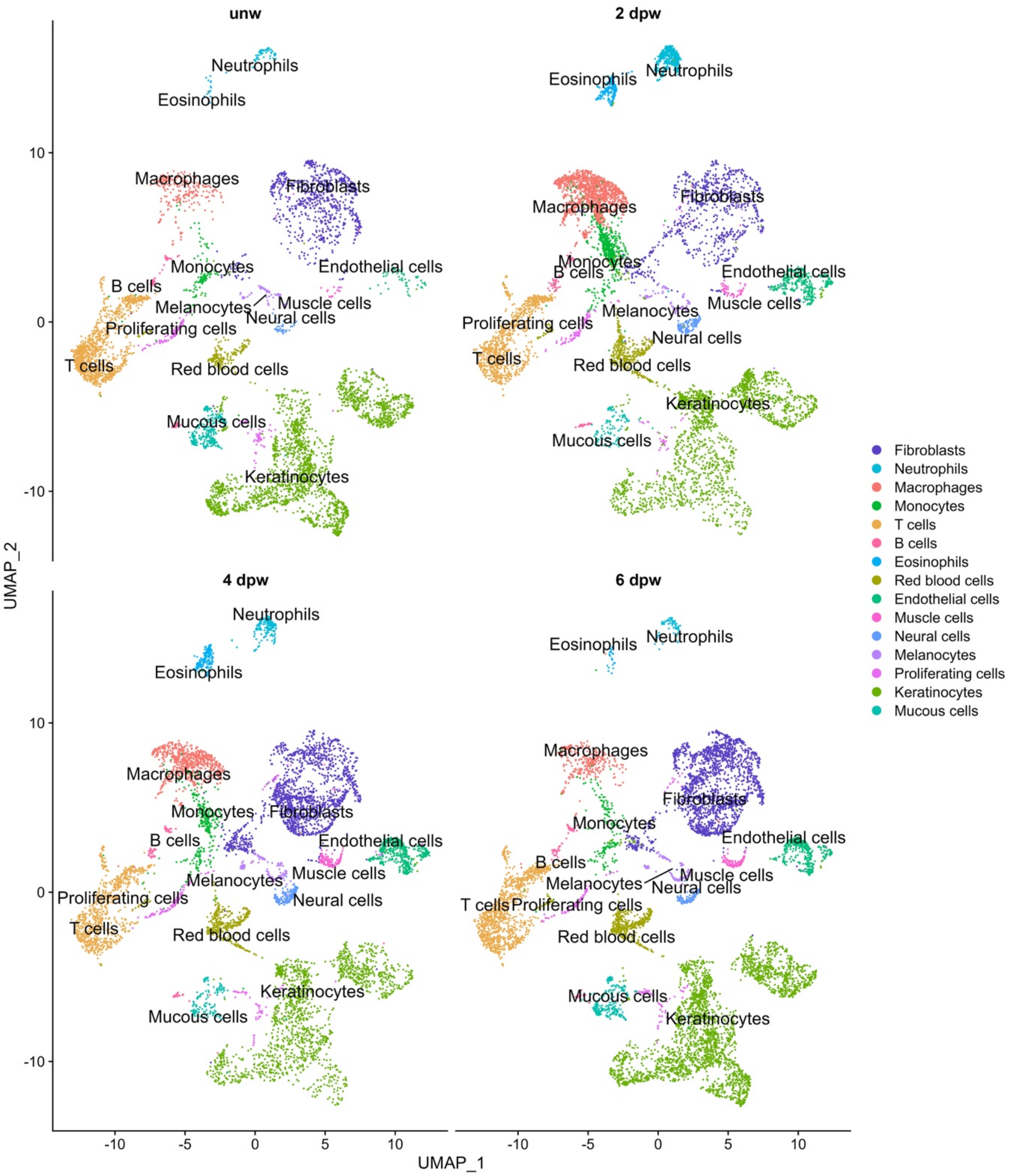

Supplement: S1 Fig — (PDF) [file pgen.1012200.s001.pdf]
