# Supplementary material for: Exploring mechanisms of scar-free skin wound healing in adult zebrafish in comparison to mouse
Source: PLoS Genet. 2026 Jun 24;22(6):e1012200. doi: 10.1371/journal.pgen.1012200 (PMC13322528; doi:10.1371/journal.pgen.1012200)

S2 Fig. Identification of different cell clusters during zebrafish wound healing.

Heat map of the top 10 differentially enriched genes in each cell cluster.

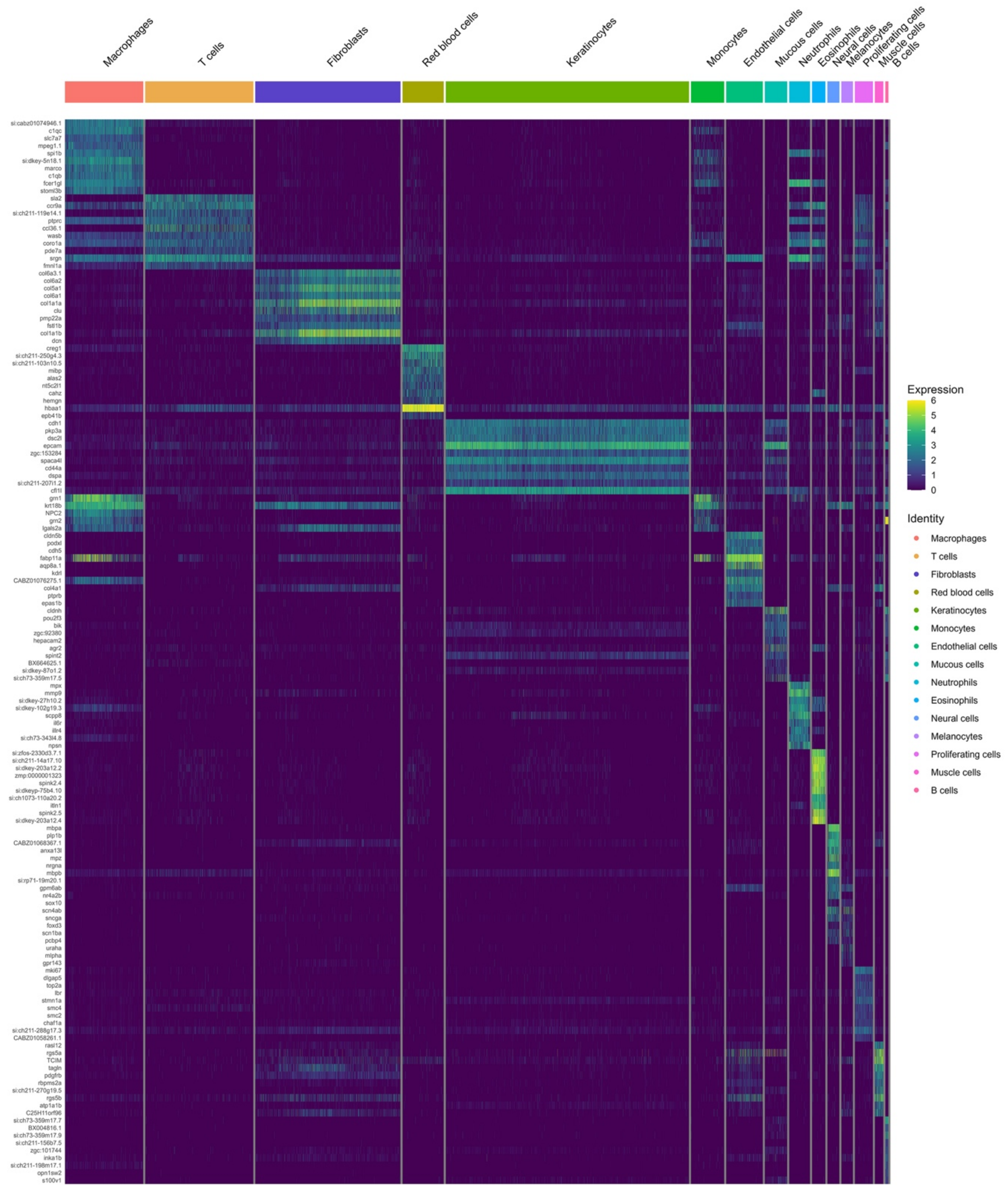

Supplement: S2 Fig — (PDF) [file pgen.1012200.s002.pdf]
