# Supplementary material for: Exploring mechanisms of scar-free skin wound healing in adult zebrafish in comparison to mouse
Source: PLoS Genet. 2026 Jun 24;22(6):e1012200. doi: 10.1371/journal.pgen.1012200 (PMC13322528; doi:10.1371/journal.pgen.1012200)

S4 Fig. The roles of macrophages during cutaneous wound healing. GO analysis of macrophage subclusters in unwounded skin and at 4 dpw and 6 dpw.

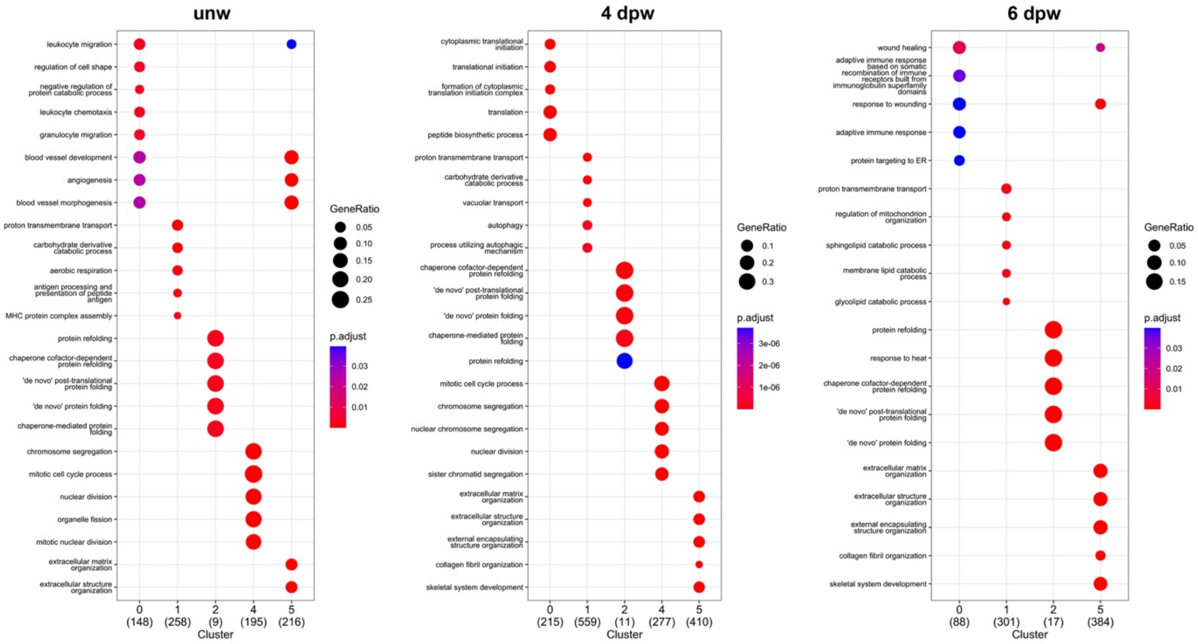

Supplement: S4 Fig — (PDF) [file pgen.1012200.s004.pdf]
