# Supplementary material for: Exploring mechanisms of scar-free skin wound healing in adult zebrafish in comparison to mouse
Source: PLoS Genet. 2026 Jun 24;22(6):e1012200. doi: 10.1371/journal.pgen.1012200 (PMC13322528; doi:10.1371/journal.pgen.1012200)
Supplement: S5 Fig — (PDF) [file pgen.1012200.s005.pdf]

S5 Fig: UMAP representations of selected macrophage-specific genes across the different stages of wound healing

(A) all clusters in unwounded skin (unw) and at 2 dpw, 4dpw, 6 dpw

(B) macrophage cluster at 2 dpw

mrc1b, cxcl19, col1a1a, col4a1, col4a2, lama4, f13a1a, esr2a, cpn1, tnfa, tgfb1a, cxcr4a, cxcr4b, il10

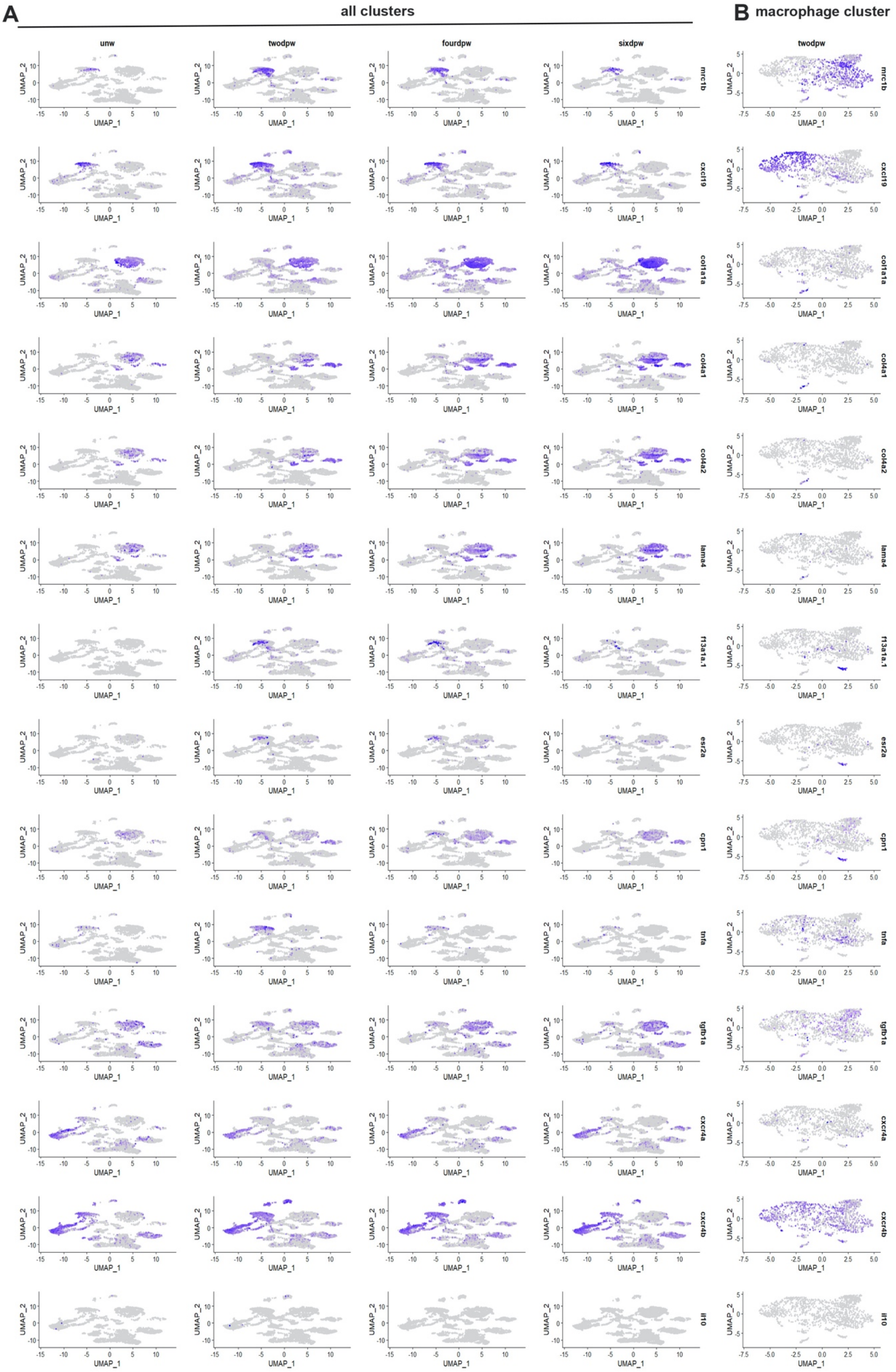

A
all clusters
B macrophage cluster
unw
twodpw
fourdpw
sixdpw
twodpw
UMAP_1
UMAP_2
mrc1b
cxcl19
col1a1a
col4a1
col4a2
lama4
f13a1a.1
esr2a
cpn1
tnfa
tgfb1a
cxcr4a
cxcr4b
il10
