# Supplementary figures and images for: Exploring mechanisms of scar-free skin wound healing in adult zebrafish in comparison to mouse
Source: PLoS Genet. 2026 Jun 24;22(6):e1012200. doi: 10.1371/journal.pgen.1012200 (PMC13322528; doi:10.1371/journal.pgen.1012200)

**S6 Fig. Double UMAP representations of selected pairs of macrophage marker genes**

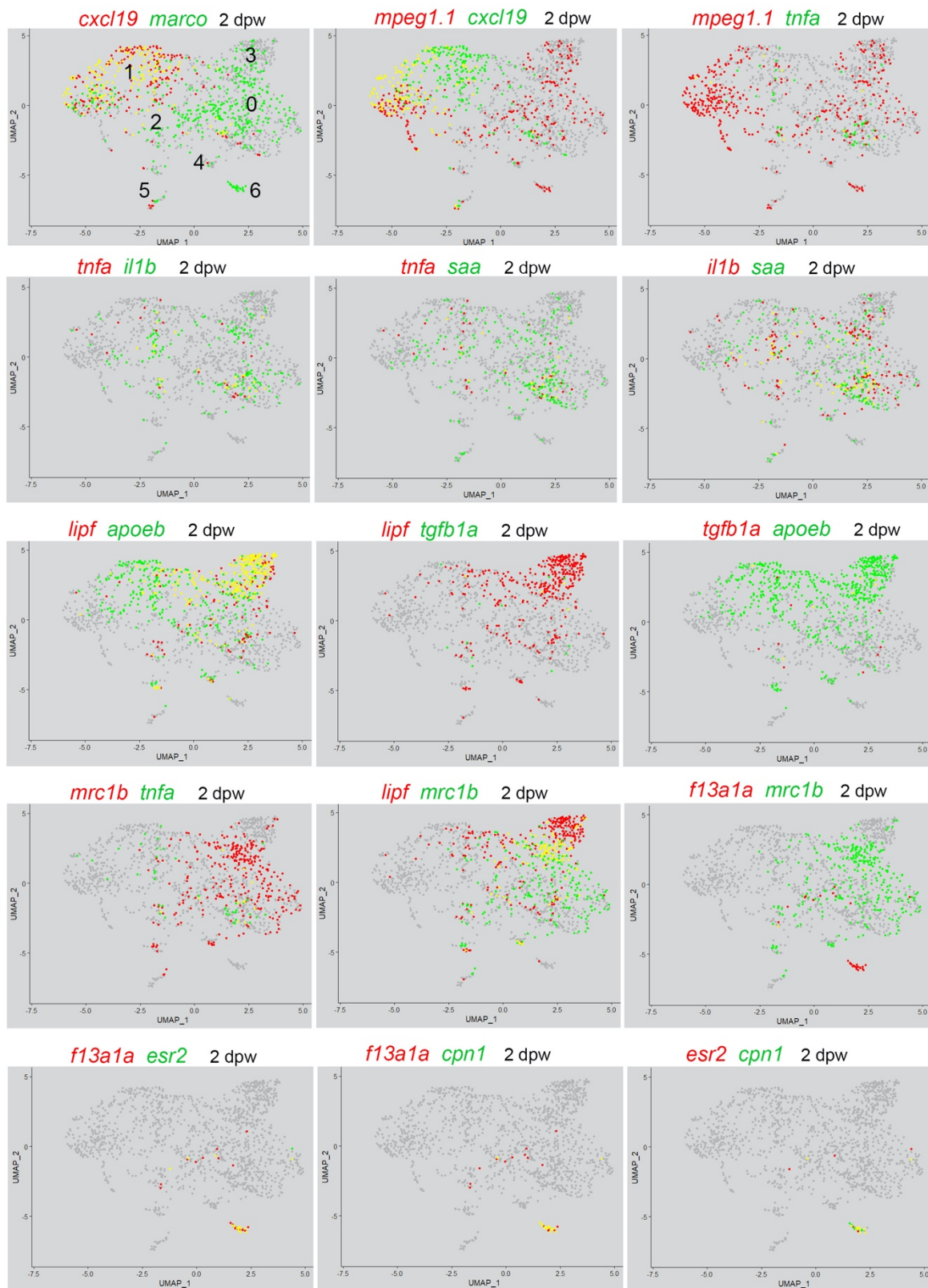

Supplement: S6 Fig — (PDF) [file pgen.1012200.s006.pdf]

**S13 Fig. Double UMAP representations of selected pairs of fibroblast marker genes**

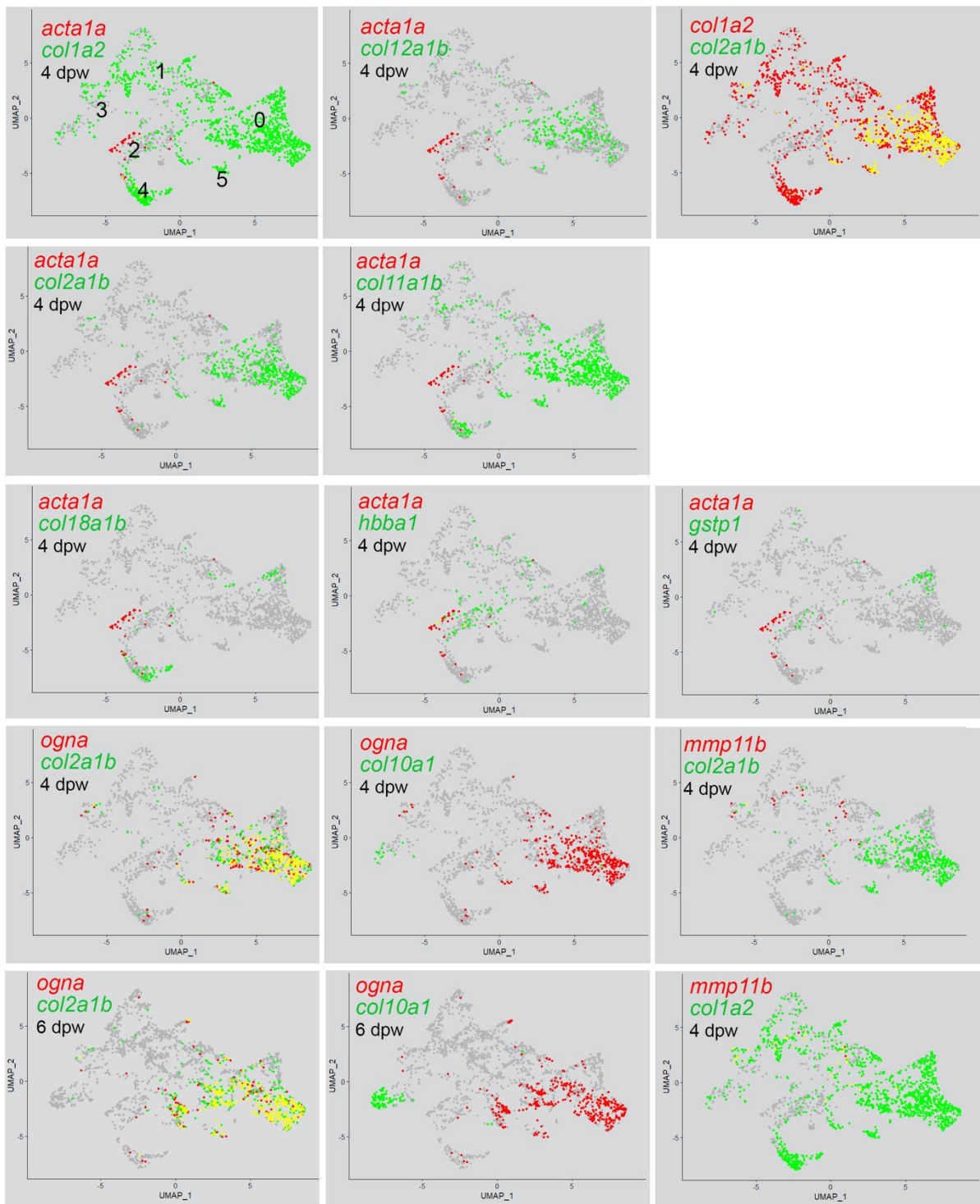

Supplement: S13 Fig — (PDF) [file pgen.1012200.s013.pdf]
