# Supplementary material for: Exploring mechanisms of scar-free skin wound healing in adult zebrafish in comparison to mouse
Source: PLoS Genet. 2026 Jun 24;22(6):e1012200. doi: 10.1371/journal.pgen.1012200 (PMC13322528; doi:10.1371/journal.pgen.1012200)

S7 Fig. Potential other contributions of innate immune cells to ECM breakdown

UMAP representations of *mmp13a* and *timp2b* (all clusters) in unwounded skin (unw) and at 2 dpw, 4 dpw and 6 dpw

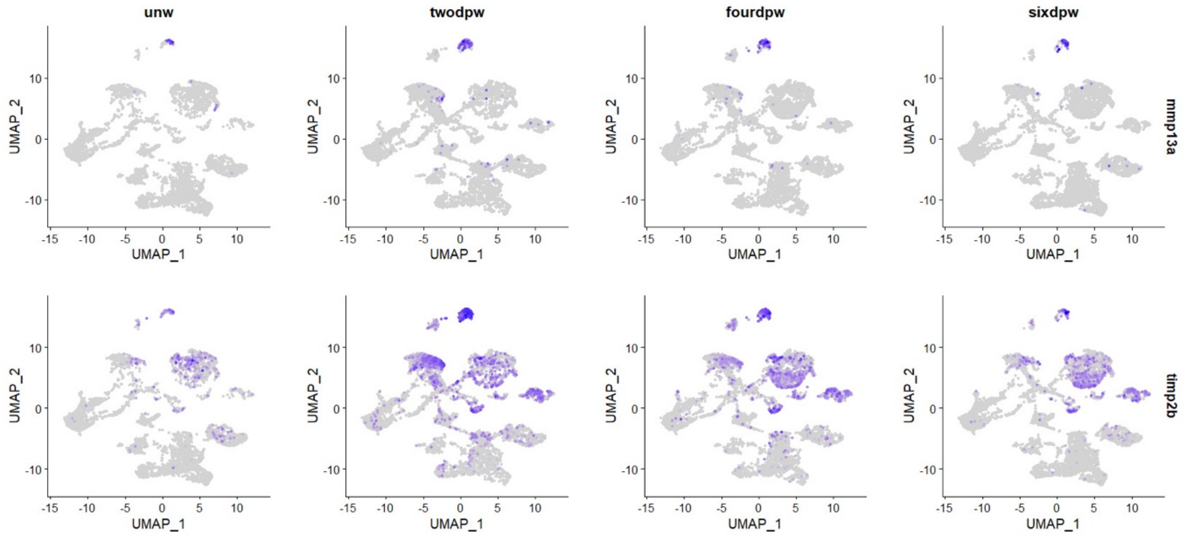

Supplement: S7 Fig — (PDF) [file pgen.1012200.s007.pdf]
