# Supplementary material for: Exploring mechanisms of scar-free skin wound healing in adult zebrafish in comparison to mouse
Source: PLoS Genet. 2026 Jun 24;22(6):e1012200. doi: 10.1371/journal.pgen.1012200 (PMC13322528; doi:10.1371/journal.pgen.1012200)

S8 Fig. Identification of fibroblast subclusters. Heat map of the top 10 differentially enriched genes in fibroblast subclusters.

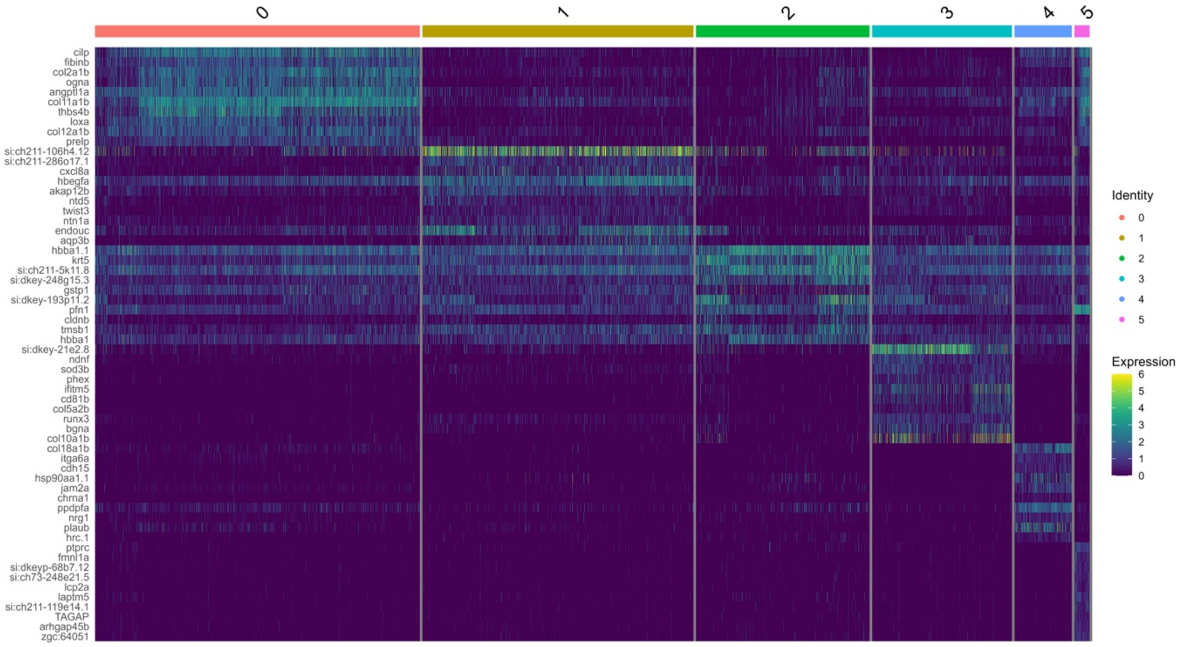

Supplement: S8 Fig — (PDF) [file pgen.1012200.s008.pdf]
