# Supplementary material for: Exploring mechanisms of scar-free skin wound healing in adult zebrafish in comparison to mouse
Source: PLoS Genet. 2026 Jun 24;22(6):e1012200. doi: 10.1371/journal.pgen.1012200 (PMC13322528; doi:10.1371/journal.pgen.1012200)
Supplement: S9 Fig — (PDF) [file pgen.1012200.s009.pdf]

S9 Fig. GO analysis of biological processes of fibroblast subclusters in unwounded skin and at 2 dpw and 6 dpw.

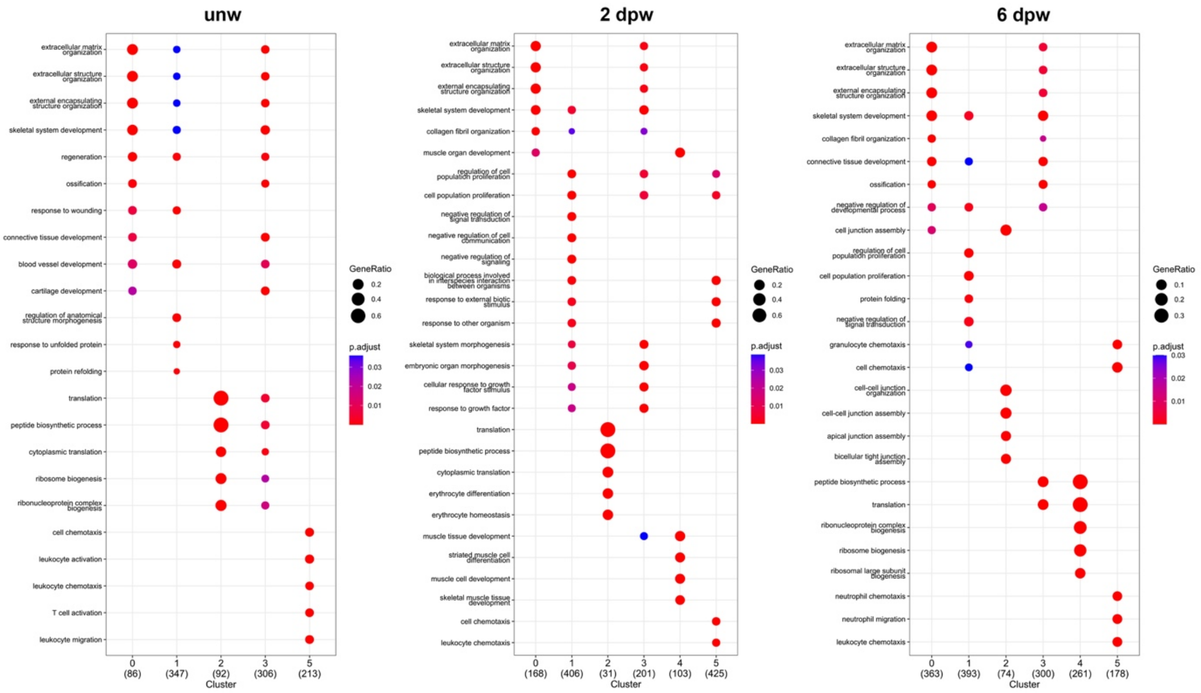

.
