# Supplementary material for: Exploring mechanisms of scar-free skin wound healing in adult zebrafish in comparison to mouse
Source: PLoS Genet. 2026 Jun 24;22(6):e1012200. doi: 10.1371/journal.pgen.1012200 (PMC13322528; doi:10.1371/journal.pgen.1012200)
Supplement: S10 Fig — (PDF) [file pgen.1012200.s010.pdf]

S10 Fig. UMAP representations of myofibroblast-specific genes and other marker fibroblast genes implicated in fibrosis across different stages of wound healing

(A) all clusters in unwounded skin (unw) and at 2 dpw, 4dpw, 6 dpw

(B) fibroblast cluster at 4 dpw

acta1a, ankrd1a, desma, cdh15, en1b, postna, postnb, cnn2a, cnn2b, fkbp10a, fkbp18b

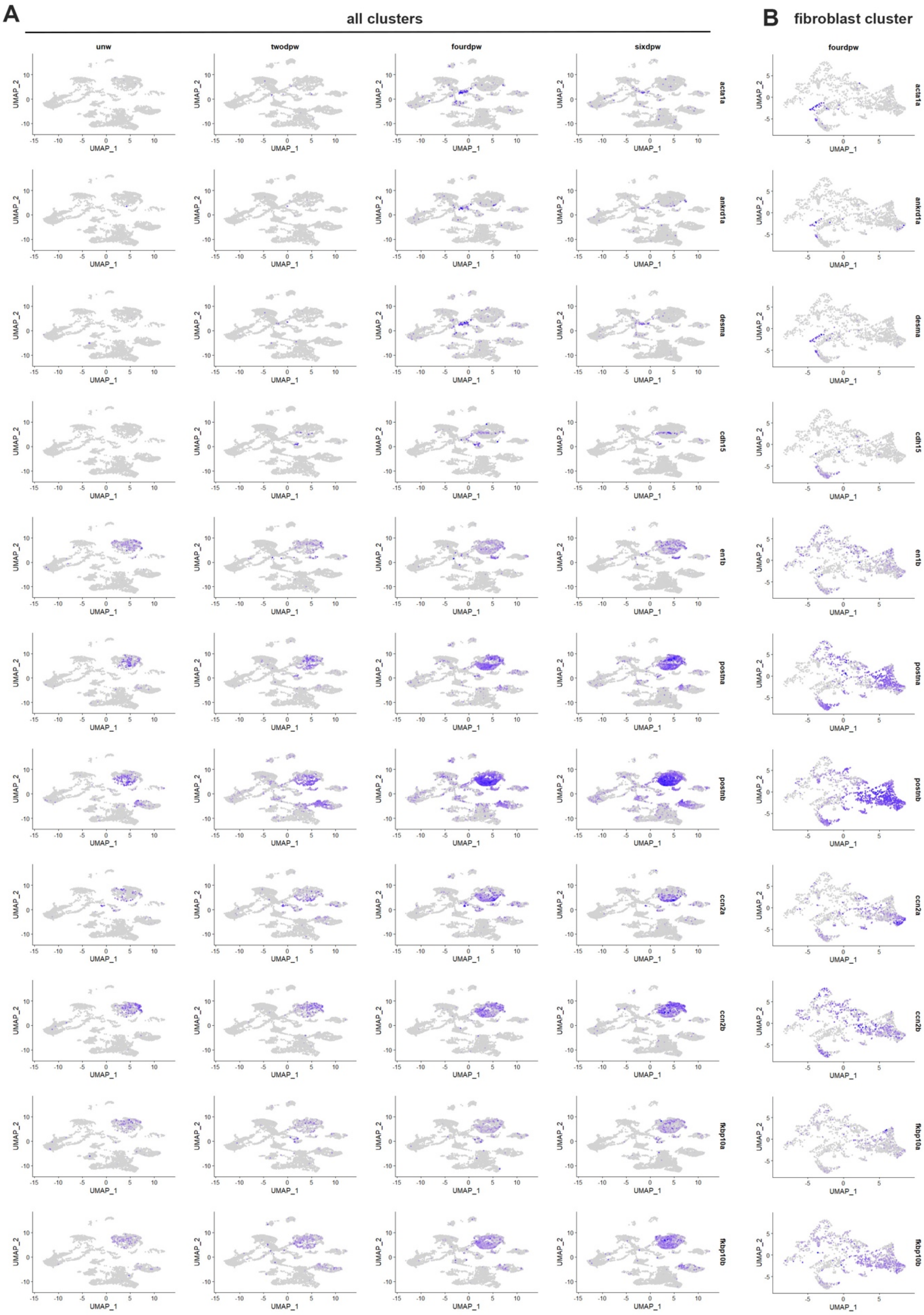
A
all clusters
unw
twodpw
fourdpw
sixdpw
B
fibroblast cluster
fourdpw
acta1a
ankrd1a
desma
cdh15
en1b
postna
postnb
ccn2a
ccn2b
fkbp10a
fkbp10b
UMAP_1
UMAP_2
