# Supplementary material for: Exploring mechanisms of scar-free skin wound healing in adult zebrafish in comparison to mouse
Source: PLoS Genet. 2026 Jun 24;22(6):e1012200. doi: 10.1371/journal.pgen.1012200 (PMC13322528; doi:10.1371/journal.pgen.1012200)
Supplement: S11 Fig — (PDF) [file pgen.1012200.s011.pdf]

S11 Fig. UMAP representations of selected collagen-encoding genes across the different stages of wound healing

(A) all clusters in unwounded skin (unw) and at 2 dpw, 4 dpw, 6 dpw

(B) fibroblast cluster at 4 dpw

col1a1a, col1a2, *col2a1b*, *col5a2b*, *col12a1b*, *col11a1b*, *col10a1a, col10a1b*, *col18a1b*, *col4a1*

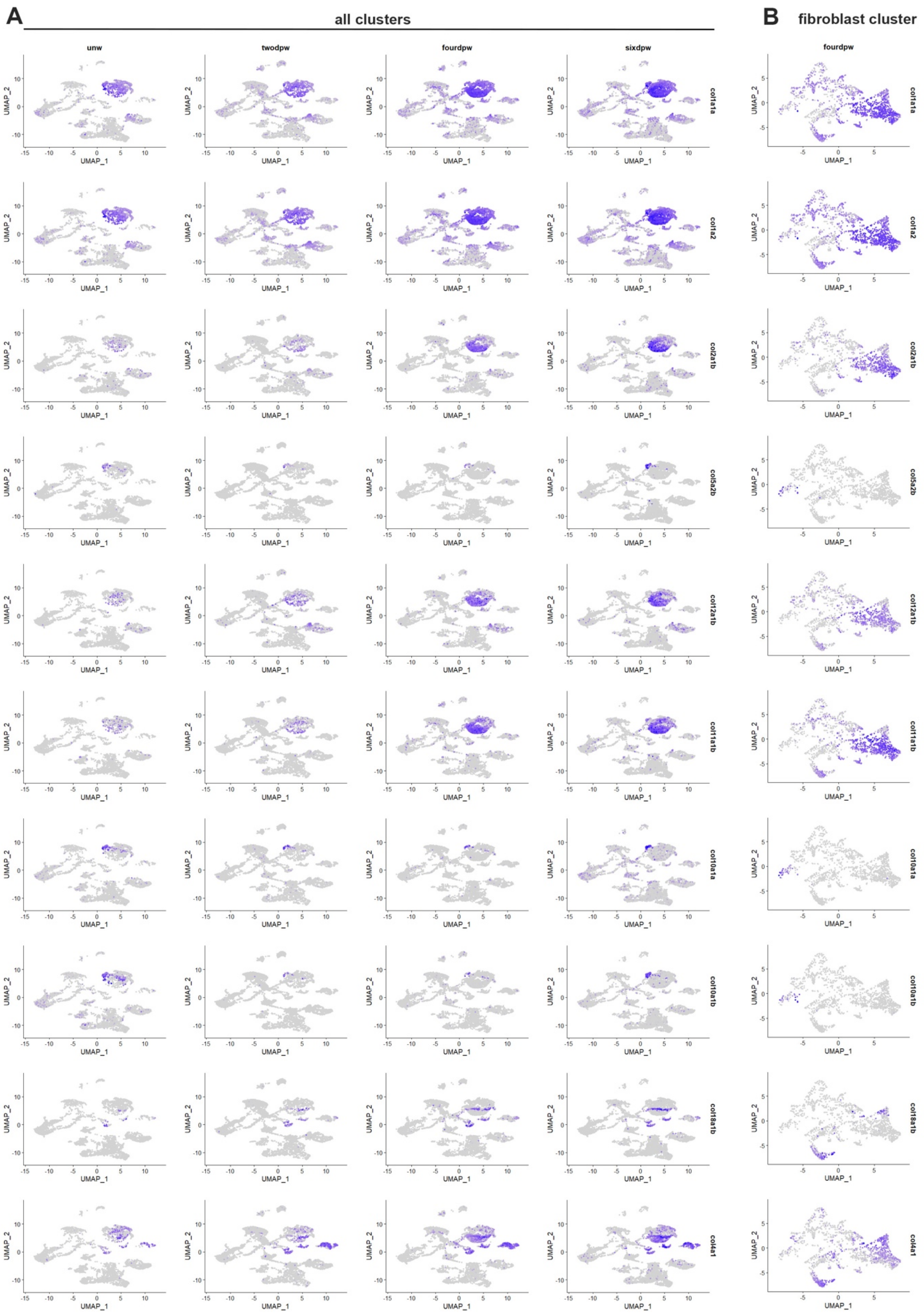
A
all clusters
B
fibroblast cluster
unw
twodpw
fourdpw
sixdpw
fourdpw
col1a1a
col1a2
col2a1b
col5a2b
col12a1b
col11a1b
col10a1a
col10a1b
col18a1b
col4a1
UMAP_1
UMAP_2
