# Supplementary material for: Exploring mechanisms of scar-free skin wound healing in adult zebrafish in comparison to mouse
Source: PLoS Genet. 2026 Jun 24;22(6):e1012200. doi: 10.1371/journal.pgen.1012200 (PMC13322528; doi:10.1371/journal.pgen.1012200)

S12 Fig. UMAP representations of selected fibroblast-specific genes across the different stages of wound healing

(A) all clusters in unwounded skin (unw) and at 2 dpw, 4 dpw, 6 dpw

(B) fibroblast at 4 dpw

fn1b, *cilp*, *ogna*, *plaub*, *mmp2, adam8a, hbba1*

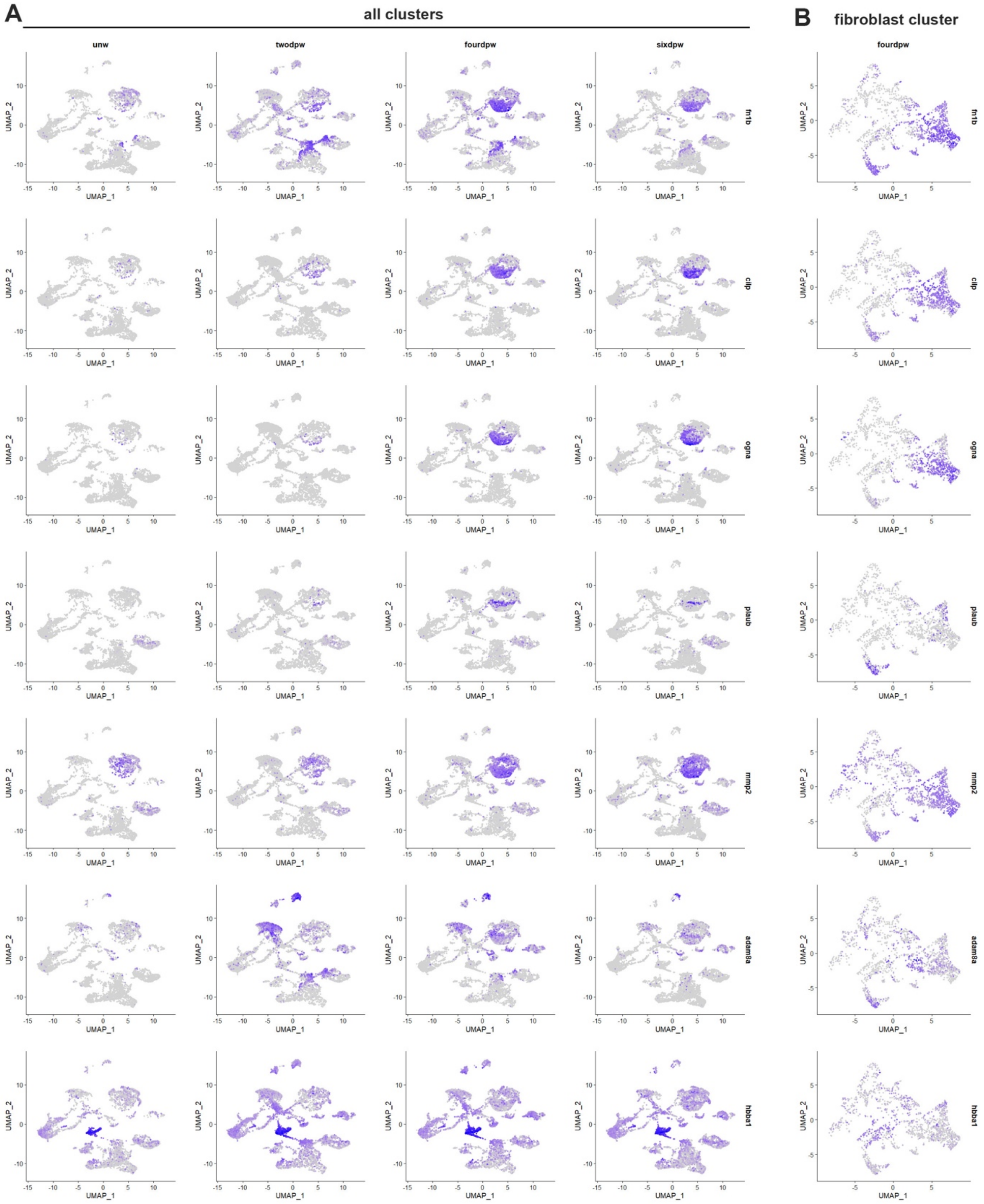

Supplement: S12 Fig — (PDF) [file pgen.1012200.s012.pdf]
