# Supplementary material for: Exploring mechanisms of scar-free skin wound healing in adult zebrafish in comparison to mouse
Source: PLoS Genet. 2026 Jun 24;22(6):e1012200. doi: 10.1371/journal.pgen.1012200 (PMC13322528; doi:10.1371/journal.pgen.1012200)

S14 Fig. Violin plots showing the expression levels of ECM build-up and ECM breakdown-related genes in different fibroblast subclusters as well as in all fibroblasts, macrophages and neutrophils together in unwounded skin and across the different phases of cutaneous wound healing.

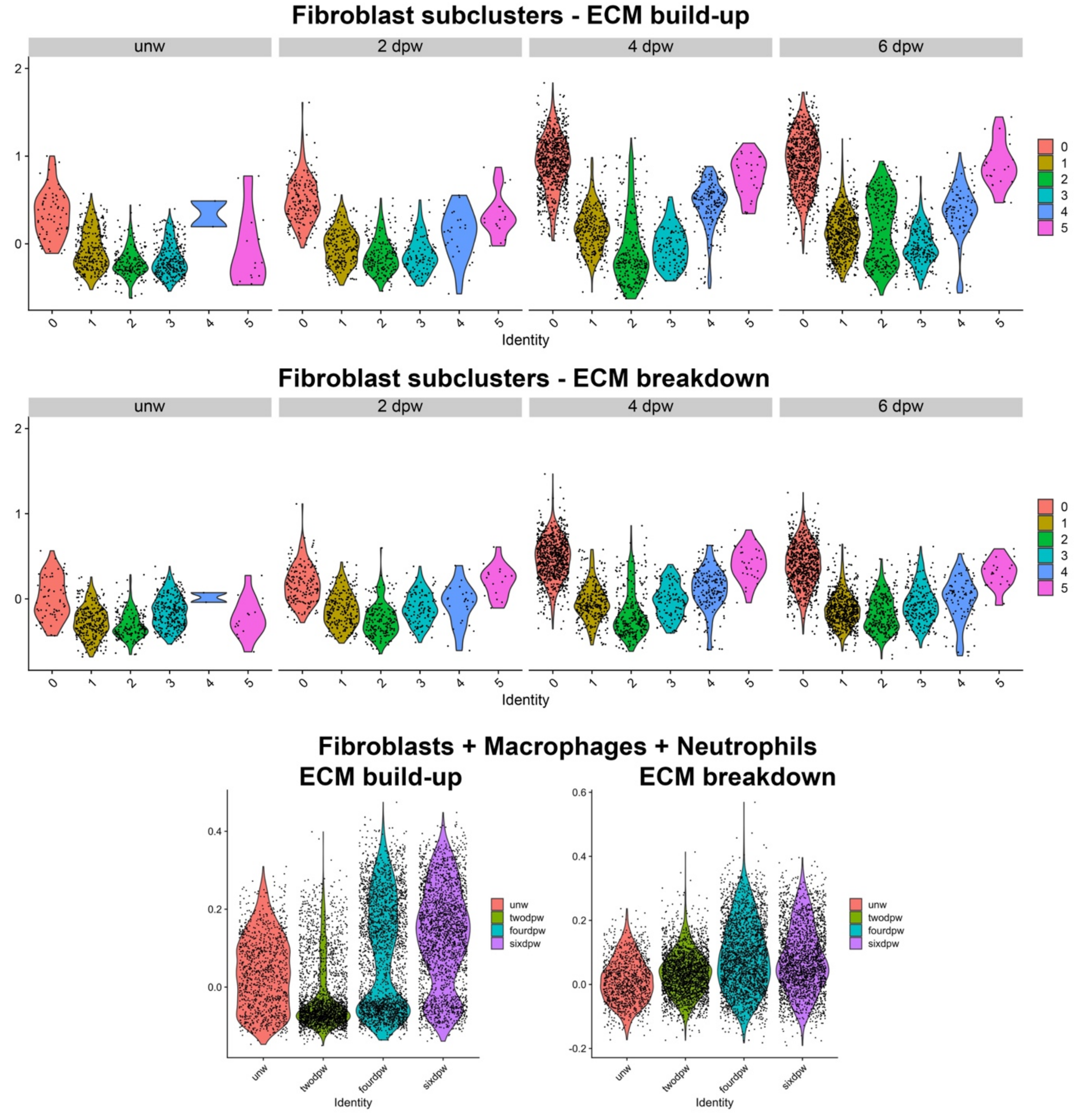

Supplement: S14 Fig — (PDF) [file pgen.1012200.s014.pdf]
