# Supplementary material for: Exploring mechanisms of scar-free skin wound healing in adult zebrafish in comparison to mouse
Source: PLoS Genet. 2026 Jun 24;22(6):e1012200. doi: 10.1371/journal.pgen.1012200 (PMC13322528; doi:10.1371/journal.pgen.1012200)
Supplement: S15 Fig — (PDF) [file pgen.1012200.s015.pdf]

S15 Fig. Expression of PDGF and TGFβ1 ligands and receptors in macrophages, neutrophils and fibroblasts

UMAP representations (all clusters) of *pdgfaa*, *pdgfab*, *pdgfba*, pdgfbb, *pdgfra*, *pdgfrb* and of *tgfb1a*, *tgfb1b*. *tgfbr1a*, *tgfbr1b*, *tgfbr2a*, *tgfbr2b* in unwounded skin (unw) and at 2 dpw, 4 dpw and 8 dpw.

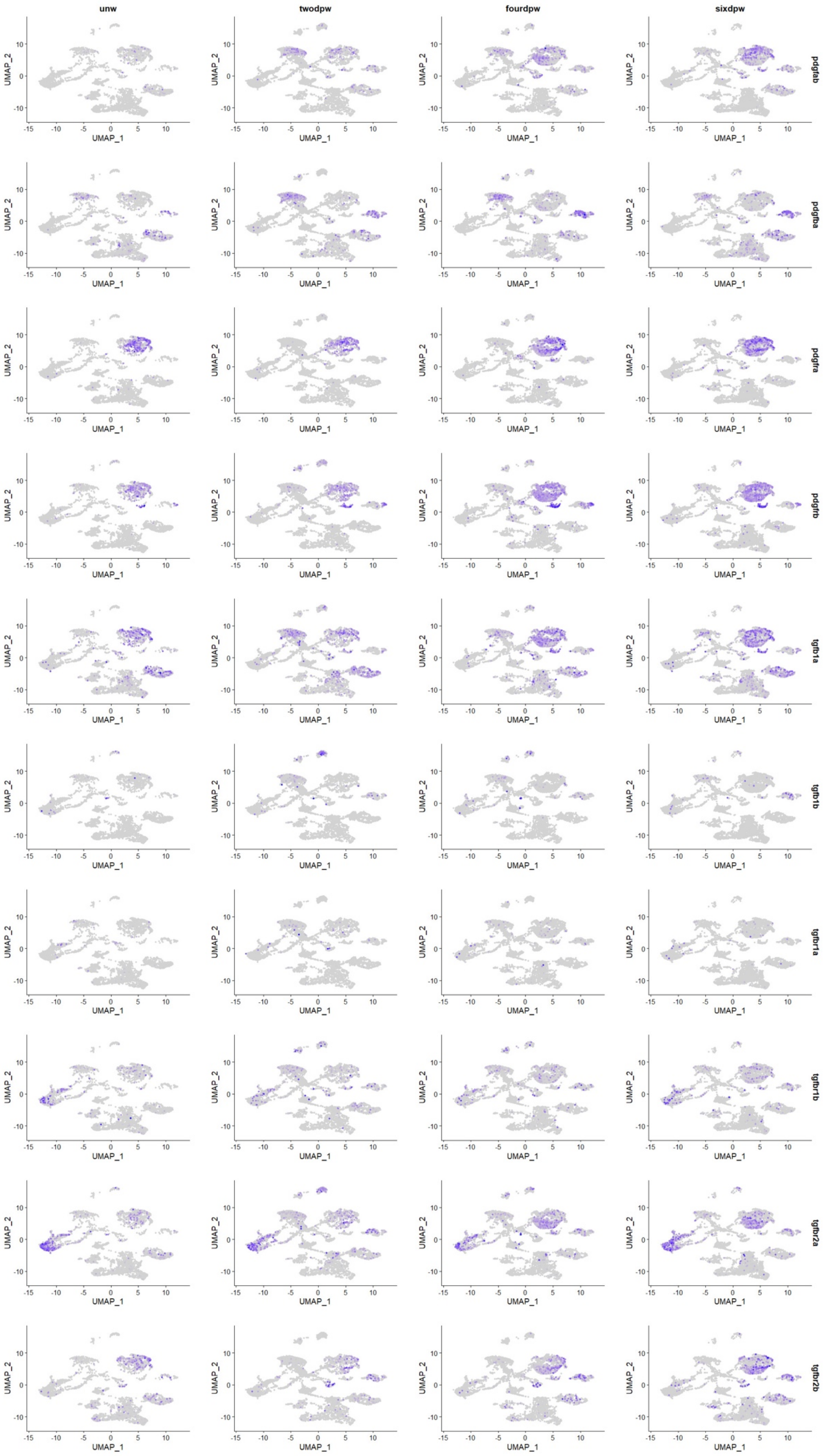
unw
twodpw
fourdpw
sixdpw
pdgfab
pdgfba
pdgfra
pdgfrb
tgfb1a
tgfb1b
tgfbr1a
tgfbr1b
tgfbr2a
tgfbr2b
UMAP_1
UMAP_2
