# Supplementary material for: Exploring mechanisms of scar-free skin wound healing in adult zebrafish in comparison to mouse
Source: PLoS Genet. 2026 Jun 24;22(6):e1012200. doi: 10.1371/journal.pgen.1012200 (PMC13322528; doi:10.1371/journal.pgen.1012200)

S16 Fig. Ligand-receptor analysis between macrophages and fibroblasts. (A) Circos plots showing the secreted signaling pathways (A), the ECM – receptor signaling pathways (B) and the cell-cell contact signaling pathways (C) from all cell clusters to fibroblasts (upper rows) and from macrophages to all cell clusters (lower rows) across the indicated time points.

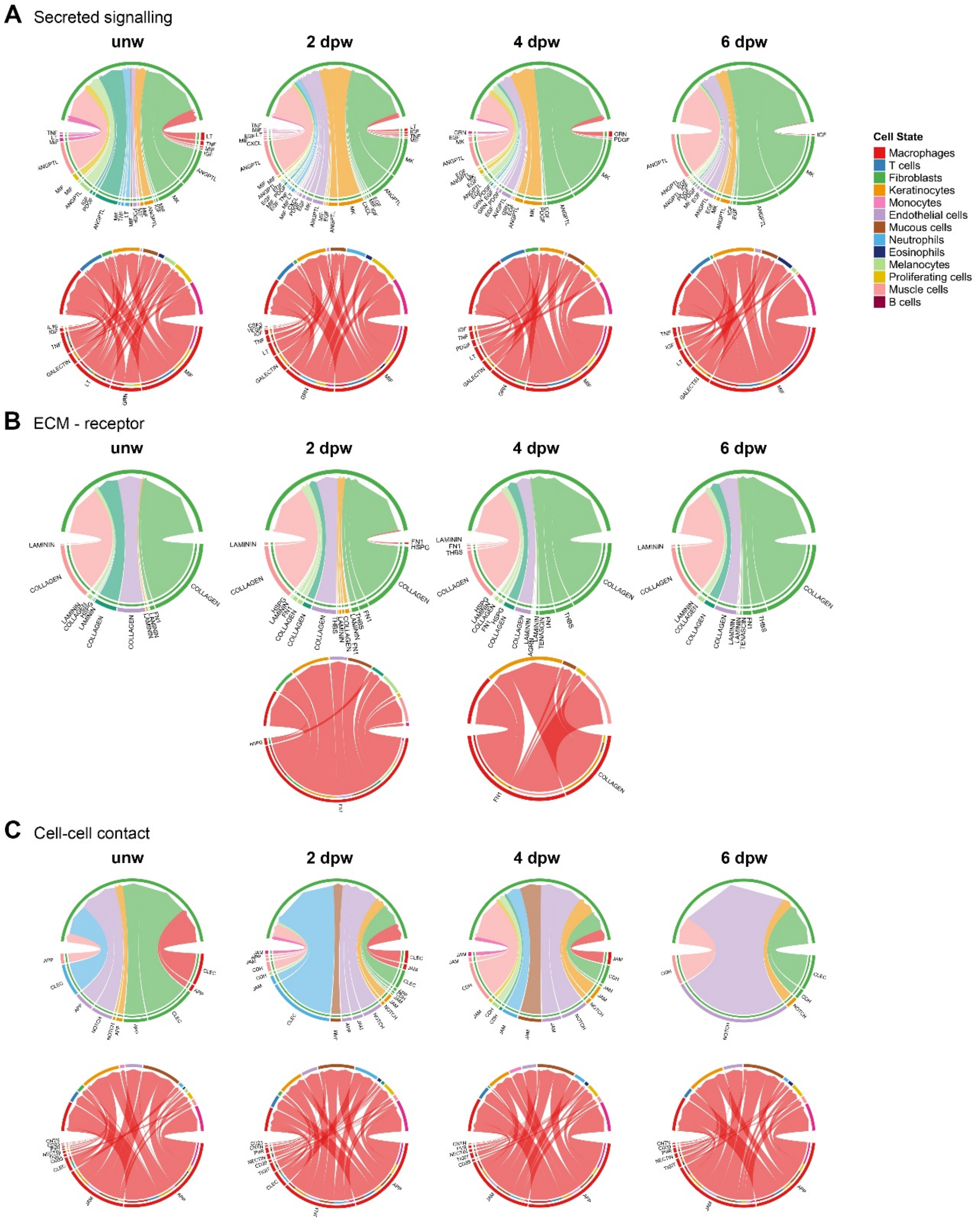

Supplement: S16 Fig — (PDF) [file pgen.1012200.s016.pdf]
