# Supplementary material for: Exploring mechanisms of scar-free skin wound healing in adult zebrafish in comparison to mouse
Source: PLoS Genet. 2026 Jun 24;22(6):e1012200. doi: 10.1371/journal.pgen.1012200 (PMC13322528; doi:10.1371/journal.pgen.1012200)

S17 Fig. Fibroblasts are spatially associated with neutrophils and macrophages

(A-D) Sections through granulation tissue at 4 dpw. (A-C) Transgenic labelling of fibroblasts (*tg(col2a1:mCherry-NTR)*) together with neutrophils (*tg(mpx:GFP)* in panel A or (*tg(lyz:GFP)* in panel B), or together with macrophages (*tg(mpeg1.1:GFP)* in panel C), counterstained with DAPI for nuclear DNA. (D) Double HCR stainings of fibroblasts (*col1a2*) and macrophages (*marco*), counterstained with DAPI for nuclear DNA.

(A'-D') UMAP presentations of different neutrophil markers (*mpx, lyz*) and macrophage markers (*mpeg1.1*, *marco*) used in (A-D) in wounds at 4dpw.

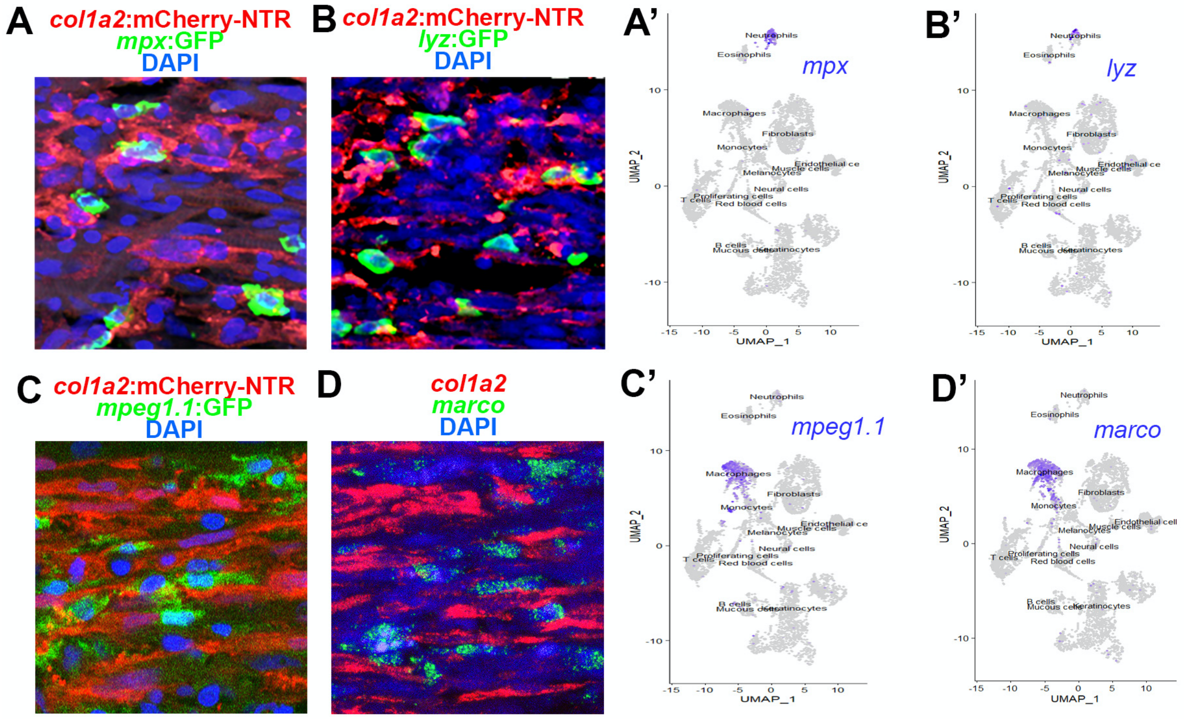

Supplement: S17 Fig — (PDF) [file pgen.1012200.s017.pdf]
