# Supplementary material for: Exploring mechanisms of scar-free skin wound healing in adult zebrafish in comparison to mouse
Source: PLoS Genet. 2026 Jun 24;22(6):e1012200. doi: 10.1371/journal.pgen.1012200 (PMC13322528; doi:10.1371/journal.pgen.1012200)

S18 Fig. Gain of Lh2 function does not compromise granulation tissue resolution. (A, B) Representative images of immunostaining against collagen I protein (gray) at 8 dpw and 12 dpw on paraffin wound sections of *Tg(hsp70l:plod2-p2A-EGFP)* fish and their control, counterstained with DAPI (blue). Scale bars: 200 µm.

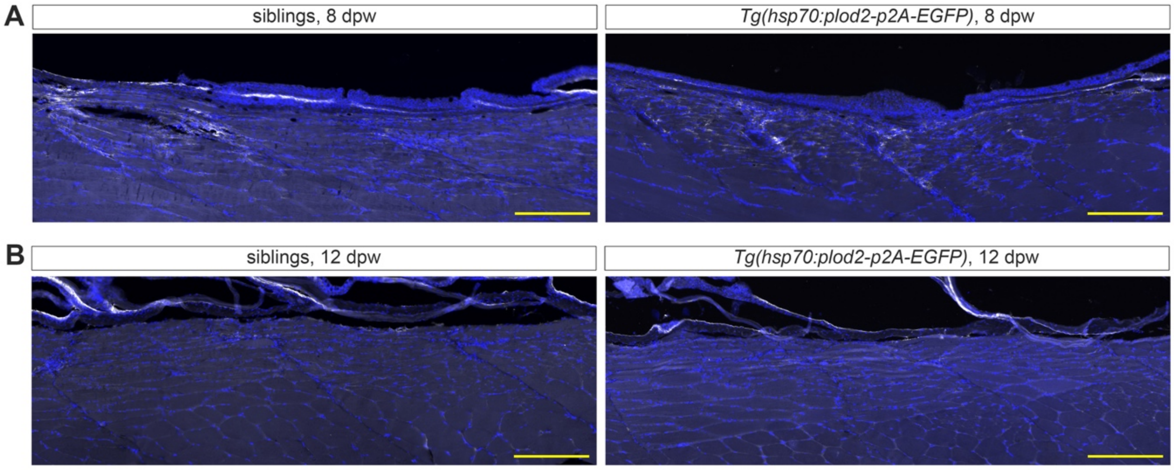

Supplement: S18 Fig — (PDF) [file pgen.1012200.s018.pdf]
