# Supplementary material for: Exploring mechanisms of scar-free skin wound healing in adult zebrafish in comparison to mouse
Source: PLoS Genet. 2026 Jun 24;22(6):e1012200. doi: 10.1371/journal.pgen.1012200 (PMC13322528; doi:10.1371/journal.pgen.1012200)
Supplement: S19 Fig — (PDF) [file pgen.1012200.s019.pdf]

S19 Fig. Genetic loss of Lh2 function does not compromise granulation tissue formation and resolution.

(A) qRT-PCR analyses of *plod1a*, *plod2* and *plod3* transcript levels in wound biopsies of *plod2* mutants compared to wild-type siblings at 4 dpw. Data are presented as fold change relative to the sibling control and columns represent the mean of three independent biological replicates (each with three technical replicates). Standard deviations (SD) were determined via two tailed, Student's *t*-test with $p<0.05$ considered statistically significant; *** $p=0.0002$, **** $p<0.0001$. (B) Ratios of HLNL to collagen, DHLNL to collagen and DHLNL to HLNL crosslinks in wound biopsies of *plod2* mutants and wild-type siblings at 4 dpw. Data represent mean ± SD; n = 6 for siblings; n=6 for mutants. (C) H&E staining on sections of *plod2* mutant fish and their control wound sections at 4 dpw, 8 dpw and 12 dpw, respectively (yellow dashed lines mark the granulation tissue). (D) Quantification of the granulation tissue sizes in sections as shown in (C), revealing that sizes were not changed upon loss of *plod2* compared to wild type siblings. Data represent mean ± SD, *n* = 3. (E) Representative images of immunostaining against collagen I protein (gray) at 4 dpw, 8 dpw and 12 dpw on paraffin sections, counterstained with DAPI (blue). (F) Quantification of the collagen I stained areas at 4 dpw, 8 dpw and 12 dpw showing no changes upon loss of *plod2* compared to wild-type siblings. Data represent mean ± SD, *n* = 3. Significances were determined with a two-tailed Student's t test. (G) TEM analysis of *plod2* mutant fish demonstrated that the diameters and alignment of collagen fibers was not changed at 4 dpw compared to wild-type siblings. Numerical values for panels B and D can be found in S2 Data. Scale bars: 200 µm in A and C; 200 nm in E. ep: epidermis, gt: granulation tissue.

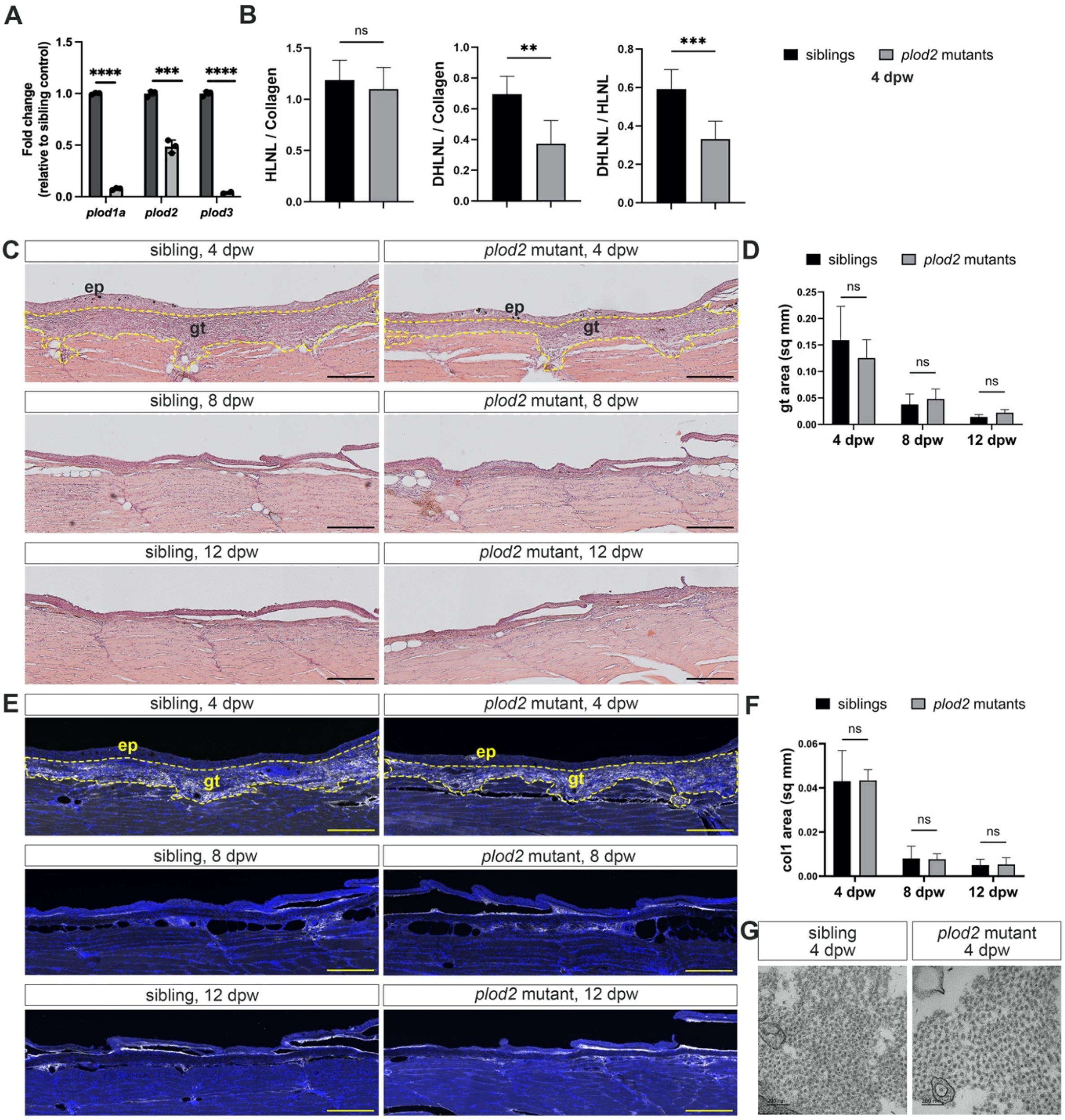

A
B
C
D
E
F
G
Fold change (relative to sibling control)
plod1a
plod2
plod3
HLNL / Collagen
DHLNL / Collagen
DHLNL / HLNL
siblings
plod2 mutants
4 dpw
sibling, 4 dpw
plod2 mutant, 4 dpw
sibling, 8 dpw
plod2 mutant, 8 dpw
sibling, 12 dpw
plod2 mutant, 12 dpw
ep
gt
gt area (sq mm)
col1 area (sq mm)
8 dpw
12 dpw
sibling
plod2 mutant
