# Supplementary material for: Exploring mechanisms of scar-free skin wound healing in adult zebrafish in comparison to mouse
Source: PLoS Genet. 2026 Jun 24;22(6):e1012200. doi: 10.1371/journal.pgen.1012200 (PMC13322528; doi:10.1371/journal.pgen.1012200)

S3 Fig. Identification of macrophage subclusters. Heat map of the top 10 differentially enriched genes in macrophage subclusters.

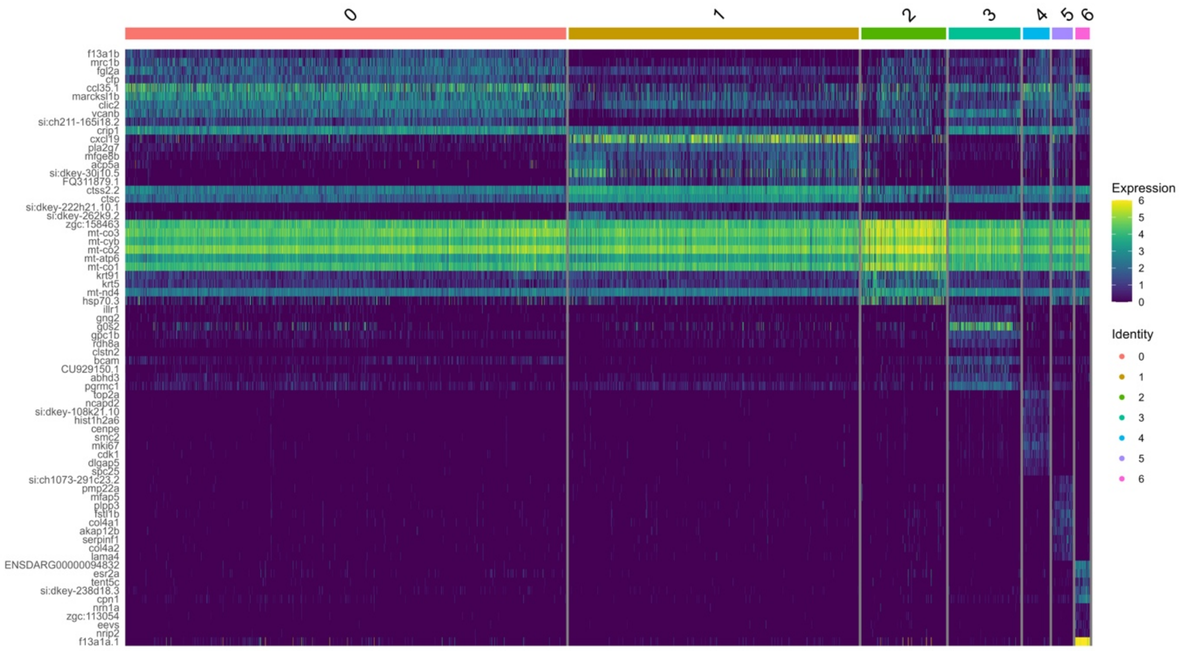

Supplement: S3 Fig — (PDF) [file pgen.1012200.s003.pdf]
