# Supplementary material for: Exploring mechanisms of scar-free skin wound healing in adult zebrafish in comparison to mouse
Source: PLoS Genet. 2026 Jun 24;22(6):e1012200. doi: 10.1371/journal.pgen.1012200 (PMC13322528; doi:10.1371/journal.pgen.1012200)
Supplement: Attachment — Submitted filename: Point-by-point account of changes.pdf [file pgen.1012200.s022.pdf]

Point-by-point account of the changes

Our manuscript PBIOLOGY-D-25-03160 entitled "Exploring mechanisms of scar-free skin wound healing in adult zebrafish in comparison to mouse" that was transferred from *PLOS Biology* to *PLOS Genetics* had not been sent out for external review.

Therefore, we did not change anything at this stage apart from adding an “Author Summary” directly behind the Abstract.
