# Supplementary material for: Exploring mechanisms of scar-free skin wound healing in adult zebrafish in comparison to mouse
Source: PLoS Genet. 2026 Jun 24;22(6):e1012200. doi: 10.1371/journal.pgen.1012200 (PMC13322528; doi:10.1371/journal.pgen.1012200)
Supplement: Attachment — Submitted filename: Point-to-Point Responses_2.pdf [file pgen.1012200.s023.pdf]

Point-to-Point Responses to Reviewers

We thank the two reviewers for their insightful comments on the first version of our manuscript, and for their very helpful suggestions for further experiments and clarifications. As specified below (in blue and italic text), we have in our revisions addressed of all their comments.

Reviewer #1:

Zebrafish have the capacity to regenerate most tissues and organs. During skin wounding, rather than forming a scar, the skin heals completely. The authors here characterize macrophage and fibroblast populations during the healing process and focus on the role of collagen crosslinking during skin regeneration. Using scRNAseq they describe different macrophage and fibroblast clusters that are to some extent dynamically overrepresented at different times of the healing process. The overall conclusion is that there is a co-existence of inflammatory and anti-inflammatory macrophage populations, confirming the emerging view that a clear distinction between M1 and M2 macrophages is rather old-fashioned and does not reflect a pathophysiological situation. Regarding fibroblasts, a main finding is that the phases of expression of ECM building genes and ECM degrading genes does not reflect the fibrotic tissue dynamics seen using histological stainings or biochemical analysis of collagen crosslinking. The authors argue that this might be because other cell types (e.g. macrophages) also contribute to fibrotic tissue remodeling.
In a second part of the project, the authors focus the attention on plod2, enconding lysyl hydroxylase 2. They pick this gene because they had studied it in the context of wound healing in the mouse. They hypothesize that alterations in cross-linking dynamics might be the basis of regeneration (differences in collagen stiffness might make collagen more easy to degrade). In the zebrafish, plod2 expression is downstream of tgf-b signaling and peaks in expression at 4 days post wounding (dpw). This was the first observation that surprised the authors, they expected lower levels, less mature collagen, easier to degrade. To study the role of plod2, the authors use a heat shock inducible gain of function line as well as a plod2 null mutant line. To their surprise, they were not able to observe alterations in the dynamics of granulation tissue regression upon gain and loss of function of plod2. They conclude that alterations in crosslinking are not the basis of fibrosis regression in zebrafish.

General comment: I find both parts of the manuscript quite disconnected. The authors should make the rational of joining both parts more clear to the reader.
Actually, we initially had thought about publishing the two parts of the manuscript separately, but finally decided to put them together for two main reasons. First, the Lh2/Plod2 story strongly benefits from the plod/fkbp10 scRNAseq data unambiguously showing the dynamics and cell type-specificity of their expression. And secondly, the purely descriptive scRNAseq analyses benefit from functional studies of a first strong candidate that could have made the difference between fibrotic healing in mammals

and scar-free healing in zebrafish. Clearly, Lh2/Plod2 was such a strong candidate, as Relmα/retlna is – at least to our knowledge – the only described pro-fibrotic mammalian factor that is even genomically absent from fish. Surprisingly, this factor turned out negative, as evolution has found an alternative way to activate plod2 even in the absence of Relmα and to remove the granulation tissue even in the presence of mammalian-like crosslinking of collagens - which feeds back to the first part of the manuscript, pointing to other genes and cell types that should be targeted next to better understand fibrotic versus perfect wound healing. We have now added a sentence describing our reasoning to the beginning of the Plod2 part of the Results section (lines 528-530).

Major comments:

1) It would be relevant to show the spatial localization of the macrophages from the different clusters during wound regeneration. The same applies for the fibroblasts datasets. The authors could do RNAScope for at least the clusters they found more interesting.

Instead of the suggested RNAScope, we have performed exhaustive single or double Hybridization Chain Reaction (HCR) studies as well as imaging with available transgenic marker lines. In addition, to correlate the thereby revealed distribution of the different cell (sub)types with the extracellular matrix of the granulation tissue (e.g. denser versus looser organization, collagen-free lacunae, and collagen uptake by cells), we have carried out the marker gene expression analyses in the background of a newly generated knock-in line, generating and allowing us to image type I collagen (Col1a2) fused with GFP. These new results are now presented as new Fig 3 for the different macrophage subclusters and Fig 6 for the different fibroblast subclusters. Results shown for double stainings are further complemented by newly generated double UMAPs, with cells expressing one marker gene in green, cells expressing the other marker gene in red, and cells co-expressing both marker genes in yellow. These new UMAPs are now presented as new Supplementary Figures S6 Fig. for markers of different macrophage subclusters / neutrophils, and S13 Fig. for markers of different fibroblast subclusters. Interestingly, we observed striking differences in the distribution of different subclusters, with some displaying rather uniform, but others very restricted localizations. For instance, macrophages of subcluster 1 are only present in the neo-epidermis, fibroblasts of subcluster 1 (which we had not further described at all in the last version of our manuscripts) are lining the base of regenerating scales, most likely representing scale-forming fibroblasts, and myofibroblast-like cells are largely restricted to marginal regions of the granulation tissue, close to nearby skeletal muscle fibers.

Performing double HCR analyses in combination with imaging GFP-Col1a2, we also made several interesting observations that extend, support or even modify several formerly drawn conclusions. For example, we found that col10a1a-positive fibroblasts

are strictly restricted to regions with looser type collagen fibers, possibly supporting granulation tissue regression, that mrc1b-positive macrophages and mmp13a-positive neutrophils often occur in pairs, and that mrc1b-positive macrophages are often found in lacuna-like domains devoid of type I collagen fibers and often contain intracellular (vesicular?) type I collagen, supporting the notion that these macrophage subtypes phagocytose MMP-predigested collagen fibers (in line with former findings in mouse; Ref. 63). In addition, we realized that the myofibroblast-like cells, in contrast to myofibroblasts in mammalian wounds, actually lack col1a2 expression, as well as the expression of all other genes encoding fibrillar or FACIT collagens (as evident from the new double UMAPs).
In the main text, these new spatial data are now inserted at different places of the Results section, directly after the former description of each subcluster (lines 176-185, 198-201, 209-215, 220-226, 242-247, 271-279, 315-323, 352-355, 368-370, 378-382, 395/396, 408-416). The col10a1a and mrc1b data are picked up again in the Discussion, as they might point to an endochondral adipogenesis-like mechanism driving the regression of the granulation tissue, or better to say, the conversion of parts of it to subcutaneous adipose tissue (lines 704-710). The finding that myofibroblast-like cells lack collagen gene expression is now also more clearly pointed out in the Discussion (lines 724-728), and even in the Abstract of the revised manuscript (lines 12-14), describing that it actually suggests that the zebrafish myofibroblast-like cells might only share the beneficial, but not the detrimental / pro-fibrotic properties of their mammalian counterparts.

2) Line 370-371: "From our histology studies, we know that between 2 dpw and 4 dpw, the granulation tissue has in net grown, whereas between 4 dpw and 6 dpw, it has in net shrunk again (Fig 1B-D)." I do not think this statement can be made without showing a quantification of granulation tissue. This has to be included in Fig 1. Same applies to the sentence on line 379 "However, in contrast to the actual increase of granulation tissue sizes from 2 dpw to 4 dpw, and its decrease from 4 dpw to 6 dpw, expression".
We have now quantified the sizes of the granulation tissues at 2, 4, 6 dpw from sections of three fish per condition (representatives of which are shown in Fig 1A-D), and present the according graph as new Fig 1F. In addition, we in the Figure legend now refer to Fig. 2j of Richardson et al., JID 2013, where we have presented a similar graph of granulation tissue sizes at 2, 4, 6, 8 dpw.

3) Figure 5: show HLNL DHLNL also at 6 dpw. Why not using in addition Sirius Red imaged using polarized light? This would allow to see difference collagen maturation (crosslinking) on the histological section gaining on top some spatial information. I encourage the authors to do this.
Luckily, we had already performed the requested additional 4 dpw HLNL/DHLNL analysis after gain- and loss of Plod2 function (see below; major point 6 of Reviewer 1 and major points 7 and 8 of Reviewer 2) a kind of pro-actively before we had received the decision letter from PLoS Genetics. Actually, the analyses were among the very

last performed by our co-author Jürgen Brinckmann before his retirement and the closure of his biochemistry facility. However, the request to also perform additional HLNL/DHLNL analysis of wild-type fish at 6 dpw came too late for Jürgen Brinckmann, and unfortunately, a commercial entity (FILK Freiberg Institute gGmbH), to which Jürgen Brinckmann had directed us, despite promising initial results failed to generate reproducible HLNL/DHLNL data. Therefore, we instead performed the Sirius Red analysis also proposed for the same purpose by Reviewer 1. Results are presented as new panels I-K of Fig 7 (formerly Fig 5). Indeed, we find that compared to 4 hpw wounds, collagen fibers in the granulation tissue of 6 dpw wounds are less dense and less mature (crosslinked), appearing yellow rather red under polarized light). The latter suggests that during granulation tissue regression, collagen crosslinking might actually be at least partly removed before collagens are fully degraded. These new findings are now described in the text in 564-575.

4) Heat shock experiments. It is unclear to me when the heat shock was performed. At 4 dpw? That would not make much sense at it is the stage of maximal expression of plod2 anyhow. It should be before and or after.
We now specify more clearly both in the text (lines 616-618) and in the legend of Fig 9 (formerly Fig 7) (lines 1634,1638,1644,1648) that heatshocks were applied DAILY, the first applied directly before wounding, and then repeated every day until fish for fixed for histology (4 dpw, 8 dpw or 12 dpw).

5) Plod2 loss of function does not alter regeneration. There are no other lysyl hydroxylases expressed that could compensate. But could there be compensation in the mutant background? I would strongly recommend to use a chemical inhibition of lysyl hydroxylase function.
We agree with the reviewer that the unaltered dynamics of granulation tissue formation and regression in plod2 mutants could be at least partly due to partial functional redundancy with plod1a and plod3 - and actually point this now out in a new paragraph added to the Discussion; lines 760-764. Therefore, ideally, one would need to do the experiment in plod1/2/3 triple mutants (if they existed and survived to adulthood). Yet, we did not follow the reviewer's recommendation to perform chemical inhibition of lysyl hydroxylase function, first because of the general reservations in the field about the specificity and feasibility of such pharmacological approaches, and second, and most importantly, because we, despite extensive literature searches, could not find any commercially available pharmacological inhibitors with proven pan-lysyl hydroxylase specificity, as required for such long-term treatments. For instance, a long-known inhibitor even applied to zebrafish, minoxidil, has been recently shown to also affect hematopoetic stem cells, making it likely that treatments for multiple days as required here would also affect wound healing via completely other means, for instance by reducing the numbers of macrophages, neutrophils and other immune cells present in the wounds.
Therefore, we instead directly looked into the mechanism proposed by the reviewer that could underlie such partial functional redundancy in plod2 mutants even though

plod1a and plod3 are not up-regulated in wounds of wild-type fish: transcriptional compensation. To do so, we performed comparative qRT-PCR of 4 dpw wound biopsies of plod2 mutants and wild-type siblings. Our data show that in mutant wounds, plod2 transcripts are decreased to approximately 50% of wild-type levels, most likely due to nonsense-mediated decay. However, also plod1a and plod3 transcript levels were decreased in wounds of plod2 mutants (even more strongly than plod2 itself), suggesting that plod2 normally has a positive effect on the expression of its two homologs. We actually had not expected this, in particular as mutants only show a halving of DHLNL cross-linking (see below for our response to major point 6), but anyway, these qPCR data clearly rule out compensatory mechanisms. The data are now shown as new panel A of S19 Fig. (formerly S16 Fig.) and mentioned in the main text in lines 634/635.

6) Both Gain and loss of function experiments of plod2, the authors don't address if there is or not an effect on crosslinking. Is now regeneration not affected because there is no crosslinking defects or does regeneration proceed normally despite effects on crosslinking? In my view this is the most important experiment to perform during the review process.

We have now added the requested DHLNL / HLNL analyses for both the gain- and the loss of plod2 function experiments (new panel C of Fig 9 (formerly Fig 7) and new panel B of S19.Fig (formerly S16 Fig)), demonstrating a 1.3-fold increase of DHLNL / HLNL ratios upon plod2 overexpression and a decrease of DHLNL / HLNL ratios to 50% of their wild-type levels in skin wounds of plod2 mutants.

We have to admit that for the plod2 gain-of-function experiment, compared to the 7-fold increase of plod2 transcript levels (and the strong staining for GFP also encoded by the transgene; new panel B of S19.Fig; see major point 7 of Reviewer 2), the obtained 1.3-fold increase in collagen crosslinking is rather low. We also point this out in the legend of Fig 9 (formerly Fig 7) (lines 1646-1657), proposing that under these particular conditions (increased plod2 expression because of wounding AND transgene activation), crucial LH2 cofactors, such as (possibly) FKBP10/FKBP65, might also become rate-limiting. Human FKBP10 has been formerly shown to potentiate the LH2-driven collagen DHLNL-HLNL crosslink switch, and its two zebrafish orthologs are also expressed in skin fibroblasts, but more moderately up-regulated upon wound healing than plod2 (UMAPs now added to S10.Fig).

Anyway, with this additional data, we can conclude that even with a further moderate increase of collagen crosslinking at stages of maximal granulation tissue dimension, granulation tissue regression occurs with normal kinetics. The same applies for the loss-of-function approach in plod2 mutants, where granulation tissue formation and regression occurs with normal kinetics although collagen crosslinking is halved. This is now described and concluded more clearly in the Results section (lines 623/624, 634/635, 640-644). In addition, we have added at paragraph to the Discussion, putting these findings into perspective with other molecular mechanisms of collagen crosslinking, possible limitations of Lh2 gain-of-function by unchanged levels of crucial

cofactors like Fkbp10, and possible limitations of Lh2 loss-of-function because of partial functional redundancy with Lh1 and Lh3 (lines 757-765).

Minor comments

1) Figure 1: is this a new experiment or a recap of experiments done previously? The way it is written it looks as if this is part of the introduction, rather than results. It is also irritating that the first panel cited is Fig 1B and not Fig 1A.

The reviewer is right that the former first paragraph of the Results section describes previous results this work is based on. We therefore have now moved it to the Introduction (lines 106-118), and have removed the references to Figure 1 (and their irritating order).

2) Line 123: “untreated” what is meant by “untreated”? At the start of the results section this irritates a bit, the part starts rather abrupt and when reading untreated I thought there would be a treatment but there isn’t one. I guess the author mean uninjured? Or it the treatment the laser injury? Treatment to me sounds more like a chemical treatment with a compound.

We apologize for this mistake and have now removed “in untreated fish,” from this sentence (which now is part of the Introduction; see our response to minor point 1).

3) Figure 2: The genes selected are not really representing always the best markers of the cluster, are they? It seems some clusters don’t have any good markers. Could the authors explain why they show these in particular in the main figure a bit more?

We have now added a sentence to the (new) paragraph introducing our spatial transcriptomics studies, stating that “Subcluster-specific markers were selected based on their subcluster specificity, their enrichment upon wounding, their GO annotation and their mentioning in recently published characterizations of different macrophage subtypes” and refer to the corresponding publications (lines 172-175).

4) Line 710 “Periostin (postna, postnb; S8 Fig.), a secreted protein made by fibroblasts that upon ablation alleviate scarring in injured hearts [71]” The article not fully correctly cited as it reports ablation of Postn-positive cells but not the role of Postn per se.

We apologize for the misunderstanding wording. In the Results section (former line 303; now 339), wording had been clear (“deletion of periostin-positive myofibroblasts reduced collagen production and scar formation”), while in the Discussion one could have referred the “that” after the “secreted protein made by fibroblasts” to “secreted protein”, rather than to “fibroblasts”. We have changed the wording to avoid this misunderstanding, now saying “Periostin, a secreted protein made by a specific fibroblasts subcluster, ablation of which alleviates scarring in injured hearts” (lines 739-741).

5) In the discussion, the authors should mention that collagens might be more stable than the RNA and therefore caution should be taken when interpreting results solely based on mRNA expression data.

We fully agree with the Reviewer about the limitations of RNA-based Matrisome AnalyzeR analyses, and have now added a paragraph right underneath the description of our Matrisome AnalyzeR data, stating "However, we also want to point out that the results of our matrisome analyses need to be taken with some caution, as they have been acquired with mRNA data, and since in contrast to their mRNAs, the different ECM proteins themselves most likely have very different stabilities, with usually very high half-lives of collagens." (lines 462-465)

Reviewer #2:

This is a very nice, solid paper that provides novel resources and mechanistic insight into the very interesting question why skin wounds heal in a scar-free manner in zebrafish. While the mechanisms underlying the elevated regenerative abilities of zebrafish in other organs are being quite heavily studied, the difference in skin wounding between mammals and more highly regenerative species like zebrafish is largely unexplored. Thus, studies dealing with this important topic are highly welcome. The manuscript presents single-cell sequencing data on zebrafish skin wounds, which are high quality and represent undoubtedly and important resource for future studies into the ability of zebrafish to heal wounds in a scar-free manner. The authors use their in-depth analysis to find that the enzyme responsible for producing collagen-crosslinks that are associated with scarring is surprisingly strongly expressed in zebrafish wounds. In a series of high-quality experiments, they – again surprisingly – show that neither gain- nor loss-of-function affects skin wound healing / scarring. While the paper thus does not provide answers for the intriguing question how scar-free healing is achieved in the zebrafish, it raises many additional intriguing questions, and strongly points towards significantly more complex, and thus much more interesting underlying mechanisms than expected. The manuscript is well written and the data generally nicely support the conclusions drawn, with the exceptions listed below. I do recommend publication if the authors can address these.

Major:

1) Fig. 1G: it's unclear whether this only shows expression in cluster 0. Results text indicates this, but legend does not mention cluster 0. Please clarify.

We assume that the Reviewer meant Fig 2G. If so, no, this dot plot shows expression levels of the indicated genes in all macrophages and selected genes representing different macrophage subclusters (now specified in the legend, line 1546). In addition, the main text has been modified to avoid this misunderstanding, now saying "Pro-inflammatory cluster 0 (Fig 2B) and expression of selected pro-inflammatory cluster 0

marker genes, alongside with anti-inflammatory marker genes of other subclusters (Fig 2G), was also present at 4 dpw and 6 dpw" (lines 234-236).

2) Fig. 4E, G: why are some FGF ligands missing from E? For a full view of which genes are expressed in which cell type, E and G both should contain the full panel of ligands.

We now mention at the end of the legend of Fig 6 (formerly Fig 4) that transcripts of Fgf ligands not shown in the plots were not found in the corresponding cell types (macrophages and/or fibroblasts at any analyzed stage (0 dpw, 2 dpw, 4 dpw, 6 dpw) (lines 1601-1603).

3) Fig. S8: according to the results text, ccn2b expression is supposed to be shown, but it missing.

The UMAP of ccn2b has been added directly underneath that of ccn2a to Fig. S8 (now S10 Fig).

4) Fig. 4, line 454. What is the basis for the statement "The majority of the interactions between macrophages and fibroblasts were via secreted signaling"? According to Fig. 4A there is NO secreted signaling interaction between fibroblasts and macrophages (legend indicates that macrophages are dark red, which is not present at all in the "secreted signaling" and "ECM-receptor" interaction plots of fibroblasts with other cells). The same applies to the statement "the Circos plots for most annotated signaling pathways also indicated signaling between neutrophils and fibroblasts". Again, in the plot in A no light blue color for neutrophils is seen, so it looks like there is NO secreted signaling interaction between fibroblasts and neutrophils.

The Reviewer is absolutely right, and we are very grateful to them for noticing this embarrassing mistake. Right now, we even cannot reconstruct ourselves how this had happened. Anyway, we have changed / corrected the text, now pointing out that there is very little interaction of fibroblasts with neutrophils as well as with macrophages (Fig 6A; formerly Fig 4A) and vice versa via secreted signaling (Fig 6A,B, formerly Fig 4A,B) (lines 507-510), but that there are relatively more interactions of macrophages with fibroblasts via ECM-receptor contacts (Fig 6B; formerly Fig 4B), and more interactions of fibroblasts with macrophages as well as with neutrophils via potential cell-cell contacts (Fig 6A; formerly Fig 4A) (lines 518-520). In addition, to further support the predicted interaction of fibroblasts with macrophages and neutrophils via cell-cell contacts, we now provide a new Supplementary Figure with co-labelings of the fibroblast-marker col1a2 with macrophage or neutrophil markers, revealing close spatial associations between these cell types on sections through granulation tissues of 4 dpw wounds (S17 Fig., text in lines 523-525).

5) qPCRs: Methods and figure legends do not indicate 1) which "housekeeping" genes were used to normalize expression levels to 2) how primer specificity and efficiency

was determined 3) what the error bars in the figures are based on (should be based on results from at least 3 biological, not technical replicates). Please clarify.

The requested information has been added to the legends of Fig 7 and Fig 9 (formerly Fig 5 and Fig 7; 3 biological replicates, lines 1611, 1636), and to the qPCR chapter of the Methods section (lines 920-927). The "house-keeping" genes rps11a and ef1alpha had formerly only been mentioned in the primer Table, but are now also named in the qPCR chapter of the Methods section.

6) Fig. 7E: Methods do not describe how the quantification was performed (e.g. cut-offs for positive pixels, whether the area was normalized to the total granulation tissue area). Please clarify.

How areas with Col1-positive signals were quantified is now described in detail in the "Quantification and statistical analysis" chapter of the Materials section (lines 1007-1015). Of note (and as also described there), the total granulation tissue area WAS also measured for each sample. However, as no significant differences in total granulation areas were observed between wild types, transgenics and mutants of the same stage (see Fig 9E / formerly 7C for average values), areas with collagen expression are reported in absolute (in mm2), rather than normalized (in %), values.

7) plod2 overexpression: While the authors show that their plod2 expression is increased in their transgenic, additional controls should be performed to support the conclusion that plod2 overexpression indeed does not affect wound healing. First, the transgene is tagged with a fluorophore, so the authors should be able to show where it is expressed. Albeit the heat-shock promoter should be active in all cells, it might not, and/or expression levels might differ massively between cell types. Since presumably only expression in fibroblasts could affect scar formation, authors should show that their transgene is expressed in fibroblasts. Second, does the overexpression affect the collagen cross-linking using the assays used in Fig. 5?

First, we have performed GFP imaging and anti-GFP immunofluorescence on 4 dpw sections of heat-shocked tg(hsp70:plod2-p2A-GFP) transgenics and non-transgenic siblings, representative examples of which are now shown as new panel B of Fig 9. As suspected by the Reviewer, expression levels in heat-shocked transgenics differed massively between cell types. Thus, there was almost no GFP expression in the keratinocytes of the neo-epidermis, but, luckily for us and the purpose of the experiment, very strong expression in cells of the granulation tissue, which according to their shape and distribution should be fibroblasts. To confirm the latter, we have also imaged GFP of heat-shocked tg(hsp70:plod2-p2A-GFP), tg(col1a2:mcherry-NTR) double transgenics, unambiguously demonstrating transgene activation in granulation tissue fibroblasts. These new data are now presented as new panel B of Fig 9 and described in the figure legend of Fig 9 (lines 1636-1643) and in the main text on lines 621/622.

Second, we now provide DHLNL/HLNL crosslink analyses as formerly shown in Fig 5, revealing a moderate (1.3-fold), but significant increase of DHLNL crosslinks and DHLNL/HLNL ratios in heat-shocked transgenics compared to heat-shocked sibling (new panel C of Fig 9). For more details, please see our comments to major point 6 of Reviewer 1.

8) plod2 loss-of-function: also here it would be very informative for interpretation of the lack of a phenotype to analyze whether collagen cross-links are affected in the mutant.

We now provide DHLNL/HLNL crosslink analyses as formerly shown in Fig 7, revealing a halving of DHLNL crosslinks and DHLNL/HLNL ratios in plod2 mutants compared to wild-type siblings. These new data have been added as new panel B to Supplementary Figure S19 (formerly S16). For more details, please see our comments to major point 6 of Reviewer 1.

Minor

1) Fig 1G: it is conceivable that the total number of cells that could be isolated and successfully sequenced is not constant between time points. Thus, showing these data as fractions of total # of cells might make more sense.

As suggested, we now show percentages of total cell numbers, rather than total cell numbers per se (now Fig 1H).

2) some figures would benefit from additional labels. E.g. the fact that 4A shows interactions with fibroblasts and B with macrophages is only found in the legend... Or by adding cartoons of treatment / heat-shock regimes.

We have added the suggested labels to Fig 6 (formerly Fig 4), as well as some arrows pointing to fibroblasts, macrophages and neutrophils in the Circos plots of panels A and B to facilitate the orientation of the reader.

3) Suggestions for edits related to language:
a) Line 11: „in addition to expressing anti-fibrotic genes”
b) line 126 “of” missing
c) line 131 “which” missing
d) line 148 probably it’s not the 10 most highly expressed genes, but the top 10 differentially enriched genes
e) line 459. delete “of”
f) line 570 delete “leads”

Language has been edited and corrected as suggested.
