# Supplementary material for: Exploring mechanisms of scar-free skin wound healing in adult zebrafish in comparison to mouse
Source: PLoS Genet. 2026 Jun 24;22(6):e1012200. doi: 10.1371/journal.pgen.1012200 (PMC13322528; doi:10.1371/journal.pgen.1012200)
Supplement: Peer review history

# Author comment: Author response to Decision Letter 0

**DOI:** 10.1371/journal.pgen.1012200.r001

**Transfer Alert**

This paper was transferred from another journal. As a result, its full editorial history (including decision letters, peer reviews and author responses) may not be present.

14 Oct 2025

Attachment Submitted filename: Point-by-point account of changes.pdf

# Peer review: Decision Letter 0

**DOI:** 10.1371/journal.pgen.1012200.r002

**Authors:** Mary Mullins, Pablo Wappner

19 Nov 2025

PGENETICS-D-25-01109

Exploring mechanisms of scar-free skin wound healing in adult zebrafish in comparison to mouse

PLOS Genetics

Dear Matthias,

Thank you for submitting your manuscript to PLOS Genetics. As you will see the reviewers have written very thoughtful and constructive reviews about your study, and each has excellent suggestions for strengthening this work. Therefore, we invite you to submit a revised version of the manuscript that addresses the points raised during the review process.

Please submit your revised manuscript by Jan 18 2026 11:59PM. If you will need more time than this to complete your revisions, please reply to this message or contact the journal office at plosgenetics@plos.org. When you're ready to submit your revision, log on to https://www.editorialmanager.com/pgenetics/ and select the 'Submissions Needing Revision' folder to locate your manuscript file.

Please include the following items when submitting your revised manuscript:

* A rebuttal letter that responds to each point raised by the editor and reviewer(s). You should upload this letter as a separate file labeled 'Response to Reviewers'. This file does not need to include responses to any formatting updates and technical items listed in the 'Journal Requirements' section below.

* A marked-up copy of your manuscript that highlights changes made to the original version. You should upload this as a separate file labeled 'Revised Manuscript with Track Changes'.

* An unmarked version of your revised paper without tracked changes. You should upload this as a separate file labeled 'Manuscript'.

If you would like to make changes to your financial disclosure, competing interests statement, or data availability statement, please make these updates within the submission form at the time of resubmission. Guidelines for resubmitting your figure files are available below the reviewer comments at the end of this letter.

We look forward to receiving your revised manuscript.

All the best,

Mary

Mary C. Mullins

Academic Editor

PLOS Genetics

Pablo Wappner

Section Editor

PLOS Genetics

Aimée Dudley

Editor-in-Chief

PLOS Genetics

Anne Goriely

Editor-in-Chief

PLOS Genetics

**Journal Requirements:**

If the reviewer comments include a recommendation to cite specific previously published works, please review and evaluate these publications to determine whether they are relevant and should be cited. There is no requirement to cite these works unless the editor has indicated otherwise.

1) Please ensure that the CRediT author contributions listed for every co-author are completed accurately and in full.

At this stage, the following Authors/Authors require contributions: İsmail Küçükaylak, Francisco Javier Martínez-Morcillo, Kai Halwas, Nils Reiche, Manuel Metzger, Petra Comelli, Jürgen Brinckmann, and Sabine Eming. Please ensure that the full contributions of each author are acknowledged in the "Add/Edit/Remove Authors" section of our submission form.

The list of CRediT author contributions may be found here: https://journals.plos.org/plosgenetics/s/authorship#loc-author-contributions

2) We ask that a manuscript source file is provided at Revision. Please upload your manuscript file as a .doc, .docx, .rtf or .tex. If you are providing a .tex file, please upload it under the item type u2018LaTeX Source Fileu2019 and leave your .pdf version as the item type u2018Manuscriptu2019.

3) Please upload all main figures as separate Figure files in .tif or .eps format. For more information about how to convert and format your figure files please see our guidelines:

https://journals.plos.org/plosgenetics/s/figures

4) Some material included in your submission may be copyrighted. According to PLOSu2019s copyright policy, authors who use figures or other material (e.g., graphics, clipart, maps) from another author or copyright holder must demonstrate or obtain permission to publish this material under the Creative Commons Attribution 4.0 International (CC BY 4.0) License used by PLOS journals. Please closely review the details of PLOSu2019s copyright requirements here: PLOS Licenses and Copyright. If you need to request permissions from a copyright holder, you may use PLOS's Copyright Content Permission form.

Please respond directly to this email and provide any known details concerning your material's license terms and permissions required for reuse, even if you have not yet obtained copyright permissions or are unsure of your material's copyright compatibility. Once you have responded and addressed all other outstanding technical requirements, you may resubmit your manuscript within Editorial Manager.

Potential Copyright Issues:

i) Figure 1E. Please confirm whether you drew the images / clip-art within the figure panels by hand. If you did not draw the images, please provide (a) a link to the source of the images or icons and their license / terms of use; or (b) written permission from the copyright holder to publish the images or icons under our CC BY 4.0 license. Alternatively, you may replace the images with open source alternatives. See these open source resources you may use to replace images / clip-art:

- https://commons.wikimedia.org

- https://openclipart.org/.

5) Please note that your Data Availability Statement is currently missing the DOI/accession number of each dataset OR a direct link to access each dataset. If your manuscript is accepted for publication, you will be asked to provide these details on a very short timeline. We therefore suggest that you provide this information now, though we will not hold up the peer review process if you are unable.

6) Kindly revise your competing statement in the online submission form to align with the journal's style guidelines: 'The authors declare that there are no competing interests.'

**Reviewers' comments:**

Reviewer's Responses to Questions

**Comments to the Authors:**

**Please note here if the review is uploaded as an attachment.**

Reviewer #1: Zebrafish have the capacity to regenerate most tissues and organs. During skin wounding, rather than forming a scar, the skin heals completely. The authors here characterize macrophage and fibroblast populations during the healing process and focus on the role of collagen crosslinking during skin regeneration. Using scRNAseq they describe different macrophage and fibroblast clusters that are to some extent dynamically overrepresented at different times of the healing process. The overall conclusion is that there is a co-existence of inflammatory and anti-inflammatory macrophage populations, confirming the emerging view that a clear distinction between M1 and M2 macrophages is rather old-fashioned and does not reflect a pathophysiological situation. Regarding fibroblasts, a main finding is that the phases of expression of ECM building genes and ECM degrading genes does not reflect the fibrotic tissue dynamics seen using histological stainings or biochemical analysis of collagen crosslinking. The authors argue that this might be because other cell types (e.g. macrophages) also contribute to fibrotic tissue remodeling.

In a second part of the project, the authors focus the attention on plod2, enconding lysyl hydroxylase 2. They pick this gene because they had studied it in the context of wound healing in the mouse. They hypothesize that alterations in cross-linking dynamics might be the basis of regeneration (differences in collagen stiffness might make collagen more easy to degrade). In the zebrafish, plod2 expression is downstream of tgf-b signaling and peaks in expression at 4 days post wounding (dpw). This was the first observation that surprised the authors, they expected lower levels, less mature collagen, easier to degrade. To study the role of plod2, the authors use a heat shock inducible gain of function line as well as a plod2 null mutant line. To their surprise, they were not able to observe alterations in the dynamics of granulation tissue regression upon gain and loss of function of plod2. They conclude that alterations in crosslinking are not the basis of fibrosis regression in zebrafish.

General comment: I find both parts of the manuscript quite disconnected. The authors should make the rational of joining both parts more clear to the reader.

Major comments:

1) It would be relevant to show the spatial localization of the macrophages from the different clusters during wound regeneration. The same applies for the fibroblasts datasets. The authors could do RNAScope for at least the clusters they found more interesting.

2) Line 370-371: “From our histology studies, we know that between 2 dpw and 4 dpw, the granulation tissue has in net grown, whereas between 4 dpw and 6 dpw, it has in net shrunk again (Fig 1B-D).” I do not think this statement can be made without showing a quantification of granulation tissue. This has to be included in Fig 1. Same applies to the sentence on line 379 “However, in contrast to the actual increase of granulation tissue sizes from 2 dpw to 4 dpw, and its decrease from 4 dpw to 6 dpw, expression»

3) Figure 5: show HLNL DHLNL and also at 6 dpw. Why not using in addition Sirius Red imaged using polarized light? This would allow to see difference collagen maturation (crosslinking) on the histological section gaining on top some spatial information. I encourage the authors to to this.

4) Heat shock experiments. It is unclear to me when the heat shock was performed. At 4 dpw? That would not make much sense at it is the stage of maximal expression of plod2 anyhow. It should be before and or after.

5) Plod2 loss of function does not alter regeneration. There are no other lysyl hydroxylases expressed that could compensate. But could there be compensation in the mutant background? I would strongly recommend to use a chemical inhibition of lysyl hydroxylase function.

6) Both Gain of loss of function experiments of plod2, the authors don’t address if there is or not an effect on crosslinking. It now regeneration not affected because there is no crosslinking defects or does regeneration proceed normally despite effects on crosslinking? In my view this is the most important experiment to perform during the review process.

Minor comments:

1) Figure 1: is this a new experiment or a recap of experiments done previously? The why it is written it looks as if this is part of the introduction, rather than results. It is also irritating that the first panel cited is Fig 1B and not Fig 1A.

2) Line 123: “untreated” what is meant by “untreated”? At the start of the results section this irritates a bit, the part starts rather abrupt and when reading untreated I thought there would be a treatment but there isn’t one. I guess the author mean uninjured ? Or it the treatment the laser injury? Treatment to me sounds more like a chemical treatment with a compound.

3) Figure 2: The genes selected are not really representing always the best markers of the cluster, are they? It seems some clusters don’t have any good markers. Could the authors explain why they show these in particular in the main figure a bit more?

4) Line 710 “Periostin (postna, postnb; S8 Fig.), a secreted protein made by fibroblasts that upon ablation alleviate scarring in injured hearts [71]” The article not fully correctly cited as it reports ablation of Postn-positive cells but not the role of Postn per se.

5) In the discussion, the authors should mention that collagens might be more stable than the RNA and therefore caution should be taken when interpreting results solely based on mRNA expression data.

Reviewer #2: This is a very nice, solid paper that provides novel resources and mechanistic insight into the very interesting question why skin wounds heal in a scar-free manner in zebrafish. While the mechanisms underlying the elevated regenerative abilities of zebrafish in other organs are being quite heavily studied, the difference in skin wounding between mammals and more highly regenerative species like zebrafish is largely unexplored. Thus, studies dealing with this important topic are highly welcome. The manuscript presents single-cell sequencing data on zebrafish skin wounds, which are high quality and represent undoubtedly and important resource for future studies into the ability of zebrafish to heal wounds in a scar-free manner. The authors use their in-depth analysis to find that the enzyme responsible for producing collagen-crosslinks that are associated with scarring is surprisingly strongly expressed in zebrafish wounds. In a series of high-quality experiments, they – again surprisingly – show that neither gain- nor loss-of-function affects skin wound healing / scarring. While the paper thus does not provide answers for the intriguing question how scar-free healing is achieved in the zebrafish, it raises many additional intriguing questions, and strongly points towards significantly more complex, and thus much more interesting underlying mechanisms than expected. The manuscript is well written and the data generally nicely support the conclusions drawn, whith the exceptions listed below. I do recommend publications if the authors can address these.

Major:

1) Fig. 1G: it’s unclear whether this only shows expression in cluster 0. Results text indicates this, but legend does not mention cluster 0. Please clarify.

2) Fig. 4E, G: why are some FGF ligands missing from E? For a full view of which genes are expressed in which cell type, E and G both should contain the full panel of ligands.

3) Fig. S8: according to the results text, ccn2b expression is supposed to be shown, but it missing.

4) Fig. 4, line 454. What is the basis for the statement “The majority of the interactions between macrophages and fibroblasts were via secreted signaling”? According to Fig. 4A there is NO secreted signaling interaction between fibroblasts and macrophages (legend indicates that macrophages are dark red, which is not present at all in the “secreted signaling” and “ECM-receptor” interaction plots of fibroblasts with other cells). The same applies to the statement “the Circos plots for most annotated signaling pathways also indicated signaling between neutrophils and fibroblasts”. Again, in the plot in A no light blue color for neutrophils is seen, so it looks like there is NO secreted signaling interaction between fibroblasts and neutrophils.

5) qPCRs: Methods and figure legends do not indicate 1) which “housekeeping” genes were used to normalize expression levels to 2) how primer specificity and efficiency was determined 3) what the error bars in the figures are based on (should be based on results from at least 3 biological, not technical replicates). Please clarify.

6) Fig. 7E: Methods do not describe how the quantification was performed (e.g. cut-offs for positive pixels, whether the area was normalized to the total granulation tissue area). Please clarify.

7) plod2 overexpression: While the authors show that their plod2 expression is increased in their transgenic, additional controls should be performed to support the conclusion that plod2 overexpression indeed does not affect wound healing. First, the transgene is tagged with a fluorophore, so the authors should be able to show where it is expressed. Albeit the heat-shock promoter should be active in all cells, it might not, and/or expression levels might differ massively between cell types. Since presumably only expression in fibroblasts could effect scar formation, authors should show that their transgene is expressed in fibroblasts. Second, does the overexpression affect the collagen cross-linking using the assays used in Fig. 5?

8) plod2 loss-of-function: also here it would be very informative for interpretation of the lack of a phenotype to analyze whether collagen cross-links are affected in the mutant.

Minor

1) Fig 1G: it is conceivable that the total number of cells that could be isolated and sucessfully sequenced is not constant between time points. Thus showing these data as fractions of total # of cells might make more sense.

2) some figures would benefit from additional labels. E.g. the fact that 4A shows interactions with fibroblasts and B with macrophages is only found in the legend... Or by adding cartoons of treatment / heat-shock regimes.

3) Suggestions for edits related to language:

a) Line 11: “in addition to expressing anti-fibrotic genes”

b) line 126 “of” missing

c) line 131 “which” missing

d) line 148 probably it’s not the 10 most highly expressed genes, but the top 10 differentially enriched genes

e) line 459. delete “of”

f) line 570 delete “leads”

**********

**Have all data underlying the figures and results presented in the manuscript been provided?**

Large-scale datasets should be made available via a public repository as described in the *PLOS Genetics*  data availability policy, and numerical data that underlies graphs or summary statistics should be provided in spreadsheet form as supporting information.

Reviewer #1: Yes

Reviewer #2: Yes

**********

PLOS authors have the option to publish the peer review history of their article (what does this mean?). If published, this will include your full peer review and any attached files.

If you choose “no”, your identity will remain anonymous but your review may still be made public.

**Do you want your identity to be public for this peer review?**  For information about this choice, including consent withdrawal, please see our Privacy Policy.

Reviewer #1: No

Reviewer #2: No

**Figure resubmission:**

-->While revising your submission, we strongly recommend that you use PLOS’s NAAS tool (https://ngplosjournals.pagemajik.ai/artanalysis) to test your figure files. NAAS can convert your figure files to the TIFF file type and meet basic requirements (such as print size, resolution), or provide you with a report on issues that do not meet our requirements and that NAAS cannot fix.

After uploading your figures to PLOS’s NAAS tool - https://ngplosjournals.pagemajik.ai/artanalysis, NAAS will process the files provided and display the results in the "Uploaded Files" section of the page as the processing is complete. If the uploaded figures meet our requirements (or NAAS is able to fix the files to meet our requirements), the figure will be marked as "fixed" above. If NAAS is unable to fix the files, a red "failed" label will appear above. When NAAS has confirmed that the figure files meet our requirements, please download the file via the download option, and include these NAAS processed figure files when submitting your revised manuscript.

**Reproducibility:**

To enhance the reproducibility of your results, we recommend that authors of applicable studies deposit laboratory protocols in protocols.io, where a protocol can be assigned its own identifier (DOI) such that it can be cited independently in the future. Additionally, PLOS ONE offers an option to publish peer-reviewed clinical study protocols. Read more information on sharing protocols at https://plos.org/protocols?utm_medium=editorial-email&utm_source=authorletters&utm_campaign=protocols

# Author comment: Author response to Decision Letter 1

**DOI:** 10.1371/journal.pgen.1012200.r003

21 May 2026

Attachment Submitted filename: Point-to-Point Responses_2.pdf

# Peer review: Decision Letter 1

**DOI:** 10.1371/journal.pgen.1012200.r004

**Authors:** Mary Mullins, Pablo Wappner, Mary Mullins, Pablo Wappner

2 Jun 2026

Dear Matthias,

We are pleased to inform you that your manuscript entitled "Exploring mechanisms of scar-free skin wound healing in adult zebrafish in comparison to mouse" has been editorially accepted for publication in PLOS Genetics. Congratulations!

Before your submission can be formally accepted and sent to production you will need to complete our formatting changes, which you will receive in a follow up email. Please be aware that it may take several days for you to receive this email; during this time no action is required by you. Please note: the accept date on your published article will reflect the date of this provisional acceptance, but your manuscript will not be scheduled for publication until the required changes have been made.

Once your paper is formally accepted, an uncorrected proof of your manuscript will be published online ahead of the final version, unless you’ve already opted out via the online submission form. If, for any reason, you do not want an earlier version of your manuscript published online or are unsure if you have already indicated as such, please let the journal staff know immediately at plosgenetics@plos.org.

In the meantime, please log into Editorial Manager at https://www.editorialmanager.com/pgenetics/, click the "Update My Information" link at the top of the page, and update your user information to ensure an efficient production and billing process. Note that PLOS requires an ORCID iD for all corresponding authors. Therefore, please ensure that you have an ORCID iD and that it is validated in Editorial Manager. To do this, go to ‘Update my Information’ (in the upper left-hand corner of the main menu), and click on the Fetch/Validate link next to the ORCID field.  This will take you to the ORCID site and allow you to create a new iD or authenticate a pre-existing iD in Editorial Manager.

If you have a press-related query, or would like to know about making your underlying data available (as you will be aware, this is required for publication), please see the end of this email. If your institution or institutions have a press office, please notify them about your upcoming article at this point, to enable them to help maximise its impact. Inform journal staff as soon as possible if you are preparing a press release for your article and need a publication date.

Thank you again for supporting open-access publishing; we are looking forward to publishing your work in PLOS Genetics!

Yours sincerely,

Mary

Mary C. Mullins

Academic Editor

PLOS Genetics

Pablo Wappner

Section Editor

PLOS Genetics

Aimée Dudley

Editor-in-Chief

PLOS Genetics

Anne Goriely

Editor-in-Chief

PLOS Genetics

www.plosgenetics.org

BlueSky: @plos.bsky.social

----------------------------------------------------

Comments from the reviewers (if applicable):

Reviewer's Responses to Questions

**Comments to the Authors:**

**Please note here if the review is uploaded as an attachment.**

Reviewer #2: The authors have addressed all issue I raised in a satisfactory manner. They have provided new data that further strengthen an already strong manuscript. I strongly recommend publication in PLOS Genetics.

**********

**Have all data underlying the figures and results presented in the manuscript been provided?**

Large-scale datasets should be made available via a public repository as described in the *PLOS Genetics*  data availability policy, and numerical data that underlies graphs or summary statistics should be provided in spreadsheet form as supporting information.

Reviewer #2: None

**********

PLOS authors have the option to publish the peer review history of their article (what does this mean?). If published, this will include your full peer review and any attached files.

If you choose “no”, your identity will remain anonymous but your review may still be made public.

**Do you want your identity to be public for this peer review?**  For information about this choice, including consent withdrawal, please see our Privacy Policy.

Reviewer #2: No

----------------------------------------------------

**Data Deposition**

If you have submitted a Research Article or Front Matter that has associated data that are not suitable for deposition in a subject-specific public repository (such as GenBank or ArrayExpress), one way to make that data available is to deposit it in the Dryad Digital Repository. As you may recall, we ask all authors to agree to make data available; this is one way to achieve that. A full list of recommended repositories can be found on our website.

The following link will take you to the Dryad record for your article, so you won't have to re‐enter its bibliographic information, and can upload your files directly:

http://datadryad.org/submit?journalID=pgenetics&manu=PGENETICS-D-25-01109R1

More information about depositing data in Dryad is available at http://www.datadryad.org/depositing. If you experience any difficulties in submitting your data, please contact help@datadryad.org for support.

Additionally, please be aware that our data availability policy requires that all numerical data underlying display items are included with the submission, and you will need to provide this before we can formally accept your manuscript, if not already present.

----------------------------------------------------

**Press Queries**

If you or your institution will be preparing press materials for this manuscript, or if you need to know your paper's publication date for media purposes, please inform the journal staff as soon as possible so that your submission can be scheduled accordingly. Your manuscript will remain under a strict press embargo until the publication date and time. This means an early version of your manuscript will not be published ahead of your final version. PLOS Genetics may also choose to issue a press release for your article. If there's anything the journal should know or you'd like more information, please get in touch via plosgenetics@plos.org.

# Editor report: Acceptance letter

**DOI:** 10.1371/journal.pgen.1012200.r005

**Authors:** Mary Mullins, Pablo Wappner, Mary Mullins, Pablo Wappner

PGENETICS-D-25-01109R1

Exploring mechanisms of scar-free skin wound healing in adult zebrafish in comparison to mouse

Dear Dr Hammerschmidt,

We are pleased to inform you that your manuscript entitled "

Exploring mechanisms of scar-free skin wound healing in adult zebrafish in comparison to mouse" has been formally accepted for publication in PLOS Genetics! Your manuscript is now with our production department and you will be notified of the publication date in due course.

The corresponding author will soon be receiving a typeset proof for review, to ensure errors have not been introduced during production. Please review the PDF proof of your manuscript carefully, as this is the last chance to correct any errors. Please note that major changes, or those which affect the scientific understanding of the work, will likely cause delays to the publication date of your manuscript.

Soon after your final files are uploaded, unless you have opted out or your manuscript is a front-matter piece, the early version of your manuscript will be published online. The date of the early version will be your article's publication date. The final article will be published to the same URL, and all versions of the paper will be accessible to readers.

For Research Articles, you will receive an invoice from PLOS for your publication fee after your manuscript has reached the completed accept phase. If you receive an email requesting payment before acceptance or for any other service, this may be a phishing scheme. Learn how to identify phishing emails and protect your accounts at https://explore.plos.org/phishing.

Thank you again for supporting PLOS Genetics and open-access publishing. We are looking forward to publishing your work!

With kind regards,

Zsofia Freund

PLOS Genetics

On behalf of:

The PLOS Genetics Team

Carlyle House, Carlyle Road, Cambridge CB4 3DN | United Kingdom

plosgenetics@plos.org | +44 (0) 1223-442823

plosgenetics.org | Twitter: @PLOSGenetics
